# Structural and functional characterization of a new thermophilic-like OYE from *Aspergillus flavus*

**DOI:** 10.1007/s00253-023-12963-w

**Published:** 2024-01-13

**Authors:** Na Li, Yuan Wang, Yinyin Meng, Yangyong Lv, Shuaibing Zhang, Shan Wei, Pingan Ma, Yuansen Hu, Hui Lin

**Affiliations:** 1 College of Biological Engineering, Henan Unsssiversity of Technology, 100 Lianhua Street, Zhengzhou, 450001 Henan China; 2 Henan International Joint Laboratory of Biocatalysis and Bio-Based Products, College of Life Sciences, Henan Agricultural University, 95 Wenhua Road, Zhengzhou, 450002 China

**Keywords:** Ene-reductase, *Aspergillus flavus*, Thermostability, Asymmetric reduction, Biocatalysis

## Abstract

**Abstract:**

Old yellow enzymes (OYEs) have been proven as powerful biocatalysts for the asymmetric reduction of activated alkenes. Fungi appear to be valuable sources of OYEs, but most of the fungal OYEs are unexplored. To expand the OYEs toolbox, a new thermophilic-like OYE (*Af*OYE1) was identified from *Aspergillus flavus* strain NRRL3357. The thermal stability analysis showed that the *T*_1/2_ of *Af*OYE1 was 60 °C, and it had the optimal temperature at 45 °C. Moreover, *Af*OYE1 exhibited high reduction activity in a wide pH range (pH 5.5–8.0). *Af*OYE1 could accept cyclic enones, acrylamide, nitroalkenes, and α, β-unsaturated aldehydes as substrates and had excellent enantioselectivity toward prochiral alkenes (> 99% ee). Interestingly, an unexpected (*S*)-stereoselectivity bioreduction toward 2-methylcyclohexenone was observed. The further crystal structure of *Af*OYE1 revealed that the “cap” region from Ala132 to Thr182, the loop of Ser316 to Gly325, α short helix of Arg371 to Gln375, and the C-terminal “finger” structure endow the catalytic cavity of *Af*OYE1 quite deep and narrow, and flavin mononucleotide (FMN) heavily buried at the bottom of the active site tunnel. Furthermore, the catalytic mechanism of *Af*OYE1 was also investigated, and the results confirmed that the residues His211, His214, and Tyr216 compose its catalytic triad. This newly identified thermophilic-like OYE would thus be valuable for asymmetric alkene hydrogenation in industrial processes.

**Key points:**

- *A new thermophilic-like OYE AfOYE1 was identified from Aspergillus flavus, and the T*_1/2_ *of AfOYE1 was 60 °C*
- *AfOYE1 catalyzed the reduction of 2-methylcyclohexenone with (S)-stereoselectivity*
- *The crystal structure of AfOYE1 was revealedv*

**Supplementary Information:**

The online version contains supplementary material available at 10.1007/s00253-023-12963-w.

## Introduction

The asymmetric reduction of C = C double bonds is widely applied in the pharmaceutical, fine chemical, and agrochemical industries, as up to two stereogenic centers can be created in those processes. Unfortunately, the chemo-catalyzed reduction of olefins using hydrogen gas and transition metal catalysts with expensive chiral ligands suffers from poor environmental sustainability, narrow substrate range, or low enantioselectivity (Hollmann et al. 2021). The biocatalyzed reduction provides an alternative green synthetic route with a broad substrate range and excellent enantioselectivity. Ene-reductases (ERs), NAD(P)H-dependent flavoproteins from the old yellow enzyme (OYEs) family, are powerful biocatalysts for the asymmetric reduction of activated alkenes, generating the products up to two new stereogenic centers with excellent enantioselectivities (Hall and Bommarius 2011; Toogood and Scrutton 2018).

Since the first OYE was isolated from yeast, many OYEs have been identified from bacteria, yeasts, fungi, plants, and algae (Parmeggiani et al. 2022). The OYEs could be used in the reduction of C = C double bonds bearing an electron-withdrawing group, such as nitro, aldehyde, ketone, carboxylic acid, ester, imide, or nitrile moiety. The enzymes of the OYE family were proposed to be divided into five subclasses based on the phylogenetic and biochemical analysis, called class I (classical OYEs), class II, class III (thermophilic-like OYEs), class IV, and class IV (fungal OYEs). Most of the known OYEs belong to the classical OYE subgroup, which contains OYE1-like OYEs from fungi, OYE3 from *Saccharomyces* sp*.*, PETNR-like OYEs from bacteria, and OPR-like OYEs from plants (Peters et al. 2019). These enzymes have been used in the synthesis of various industrial interested building blocks, such as (*R*)-citronellal (Zheng et al. 2018), amino acid derivatives (Winkler et al. 2012), and (*R*)-3-Hydroxy-2-methylpropanoate (Stueckler et al. 2010).

Thermophilic enzymes have great potential for industrial use as they have high resistance to temperature and organic solvents. Since the identification of the first thermophilic-like OYE YqjM from *Bacillus subtilis* (Kitzing et al. 2005), another 10 thermophilic-like OYEs belonging to class III have been isolated and characterized, including XenA from *Pseudomonas putida* (Griese et al. 2006), *Ts*OYE from *Thermus scotoductus* SA-01 (Opperman et al. 2008), TOYE from *Thermoanaerobacter pseudethanolicus* (Adalbjornsson et al. 2010), *Gk*OYE from *Geobacillus kaustophilus* (Schittmayer et al. 2011), *Chr*-OYE3 from *Chryseobacterium* sp. CA49 (Xu et al. 2014), *Geo*ER from *Geobacillus* sp. 30 (Tsuji et al. 2014), OYERo2 from *Rhodococcus opacus* 1CP (Riedel et al. 2015), *F*OYE-1 from *Ferrovum* sp. JA12 (Scholtissek et al. 2017), *Ca*OYE from *Chloroflexus aggregans* (Robescu et al. 2021), and *Pf*ER2 from *Pseudomonas fluorescens* (Shi et al. 2021). Moreover, four thermal-tolerant OYEs were also identified from other subclasses, including *Bc*OYE (class IV) from *Bacillus coagulans* (Zhou et al. 2014), YqiG (class IV) from *B. subtilis* (Sheng et al. 2016), *Ct*OYE (class I) from *Chroococcidiopsis thermalis*, and *Gs*OYE (class I) from *Galdieria sulphuraria* (Robescu et al. 2020). All the thermophilic-like OYEs showed activity optima around 40–55 °C exhibited high thermostability and high resistance toward organic solvents, so they have huge potential for industrial application.

Several thermophilic-like OYE structures have been revealed, including YqjM (PDB: 1Z41) (Kitzing et al. 2005), XenA (PDB: 2H8X) (Griese et al. 2006), CrS (PDB: 3HF3) (Opperman et al. 2010), TOYE (PDB: 3KRU) (Adalbjornsson et al. 2010), *Gk*OYE (PDB: 3GR7) (Schittmayer et al. 2011), *Rm*ER (PDB: 5OCS) (Opperman 2017), and *Ca*OYE (PDB: 7O0T) (Robescu et al. 2021), which showed that OYE homologs are highly conserved. The general catalytic mechanism of OYE-mediated reduction is well understood. The electron-withdrawing group moiety forms an H-bond interaction with two donor residues, usually histidine and asparagine, and then the addition of hydride from the reduced flavin to the Cβ of the activated alkene, followed by proton transfer to the Cα of the substrate from a tyrosine or cysteine residue.

Although many OYEs have been identified over the last years, the demand for new OYEs keeps growing to overcome application limitations, such as low catalytic activity and poor stability in harsh reaction conditions. Fungi appear to be valuable sources of OYEs, but most of the fungal OYEs are unexplored. *Aspergillus* sp*.* produces many secondary metabolites, and it has been proposed that ene-reductases participate in the synthetic pathways. For example, the *Aspergillus fumigatus* old yellow enzyme EasA reduces chanoclavine-I aldehyde to dihydrochanoclavine aldehyde in the ergot alkaloid pathways (Cheng et al. 2010). More recently, four OYEs were identified from *Aspergillus niger* and *Botryotinia fuckeliana* (Robescu et al. 2022a, 2022b). Herein, we mined the OYEs in the filamentous fungus *Aspergillus flavus* to expand the ene-reductase toolbox. A new thermophilic-like OYE (*Af*OYE1) was identified and carefully characterized, which exhibited broad substrate scope and excellent enantioselectivity.

## Materials and methods

### Reagents

Alkene substrates and racemic reduction products were purchased from MilliporeSigma (MA, USA), Aladdin (Shanghai, China), or TCI (Shanghai, China). Glucose dehydrogenase from *Bacillus* was purchased from Aladdin (G139687, Shanghai, China), and NADH disodium salt hydrate was purchased from TCI (Shanghai, China). All protein commercial crystallization kits were purchased from Hampton Research (CA, USA), MiTeGen LLC (NY, USA), Qiagen (Hilden, Germany), or Molecular Dimensions (OH, USA). All other reagents and solvents were commercially available and were used without further purification.

### General analysis methods

^1^H NMR spectra were acquired on a Bruker-400 spectrometer in CDCl_3_, and all signals were reported in parts per million (ppm) downfield relative to tetramethylsilane. Optical rotations were measured with a Perkin Elmer 341 polarimeter. Gas chromatographic analyses were performed on a Thermo Scientific TRACE 1300 gas chromatograph equipped with a CHIRASIL-DEX CB column (Agilent Technologies, USA), and using a flame ionization detector, nitrogen was used as the carrier gas at 5 mL/min, the split ratio was 1:20 (v/v), and the column temperature was programmed as being kept at 50 °C for 1 min and then upgraded to 150 °C at the rate of 3 °C/min. The protein purities were analyzed using SDS-PAGE using 4–12% polyacrylamide gradient gel (SurePAGE, Genscript, Nanjing, China) running in 50 mM MOPS, 50 mM Tris base, 0.1% SDS, and 1 mM EDTA, at pH 7.7, and stained with Coomassie Blue. Protein concentrations were measured using the Bradford assay (Coomassie Brilliant Blue G-250) and bovine serum albumin as the protein standard.

### Mining OYE from Aspergillus flavus and plasmid construction

The thermophilic-like old yellow enzyme YqjM was submitted to a BLASTp program against the *A. flavus* strain NRRL3357 proteins in UniProtKB/Swiss-Prot database, which returned an NADPH dehydrogenase afvA (UniProt: B8N8Q9, GenBank: XP_041144250.1) with 41.7% identities and an NADP-dependent oxidoreductase lnbE (UniProt: B8NWW6) with 34% identities. Here, the NADPH dehydrogenase afvA was selected, and its gene sequence with an N-terminal His6-tag followed by a tobacco etch virus (TEV) protease cleavage site was codon-optimized by OPTIMIZER (http://genomes.urv.es/OPTIMIZER/, See Supplementary Information for nucleotide sequence) (Puigbò et al. 2007) for the expression in *E. coli* and synthesized by GENEWIZ (Nanjing, China). The synthesized gene was inserted into the pET28b vector between the *Nco*I and *Xho*I enzyme sites, creating the plasmid pET28b-*afvA*, and the sequence was confirmed via sequencing (Shenggong, Shanghai, China).

### Protein production and purification

The plasmid pET28b-*afvA* was transferred into *E. coli* BL21 (DE3) for protein production. LB medium containing kanamycin (50 μg/mL) was inoculated with *E. coli* BL21 (pET28b-*afvA*), and the culture was incubated at 37 °C and 220 rpm. Then, isopropyl-β-D-thio-galactoside (IPTG) was added into the culture at the final concentration of 1 mM when the OD_600_ reached about 0.5. The culture was further cultured at 16 °C for 18 h for protein expression, and then the cells were harvested by centrifugation (5000 g, 4 °C) for 6 min.

The cell pellets were re-suspended in HEPES buffer (25 mM, pH 7.5, 10 M NaOH solution was used to adjust pH) containing 300 mM NaCl, 20 mM imidazole, and 1 mM tris(2-carboxyethyl)phosphine (TCEP), and then the cells were lysed by low-temperature ultra-high‐pressure homogenizer (JN-mini, JNBio, China) at 1100 bar and 4 °C. The lysis was centrifuged at 18,000 g and 4 °C for 1 h to remove the cell debris. The clear lysate was filtered through a surfactant-free cellulose acetate membrane with a 0.45 μm pore size and loaded on the His Trap™ HP column, which was pre-equilibrated with HEPES buffer (25 mM, pH 7.5) containing 300 mM NaCl, 20 mM imidazole, and 1 mM TCEP. Proteins were eluted by HEPES buffer (25 mM, pH 7.5) containing 300 mM NaCl and 1 mM TCEP with a linear imidazole concentration gradient from 20 to 500 mM. The peak fractions were collected and followed by His6-TEV protease cleavage and dialysis against HEPES buffer (25 mM, pH 7.5) containing 300 mM NaCl and 1 mM TCEP overnight. The dialysate was loaded onto a His Trap™ HP column again, which was pre-equilibrated with HEPES buffer (25 mM, pH 7.5) containing 300 mM NaCl and 1 mM TCEP. Flow through and wash fractions that contain the desired purified protein were collected and checked by SDS-PAGE. The fractions containing desired purified protein were pooled and concentrated by Amicon® Ultra-15 Centrifugal Filter Unit (10 K, Merck Millipore) and used as the catalyst.

To prepare protein samples for crystallization, the purified protein was further purified using gel filtration (HiLoad 16/60 Superdex pg 200, GE Healthcare) with HEPES buffer (25 mM, pH 7.5) containing 100 mM NaCl and 1 mM TCEP. The peak fractions were concentrated and used for crystallization.

### General procedures of AfOYE1-catalyzed reductions

The purified *Af*OYE1 was used in all the measurements. All the reaction mixtures contained 5 mL potassium phosphate buffer (100 mM, pH 7.0), 0.1 μM purified *Af*OYE1, 5 U glucose dehydrogenase from *Bacillus*, 50 mM glucose, 20 mM NADH, and 8 mM substrate, and then the reactions were conducted at 45 °C for 10 min in triplicates. Then, the mixtures were extracted with ethyl acetate (1 mL), and then the yields were determined by GC with an external standard calibration curve. One unit of enzyme activity was defined as the amount of enzyme producing 1 μmol product per minute under the assay condition.

The irreversible thermal inactivation (*T*_1/2_) of *Af*OYE1 was analyzed by heating the purified *Af*OYE1 in potassium phosphate buffer (pH 7.0) at different temperatures (30–70 °C) for 5 min, chilled on ice. The residual activities on 2-cyclopentenone were then measured under standard assay conditions.

To determine the optimal reaction conditions, 8 mM 2-cyclopentenone was used as a substrate, and the reactions were initiated by the addition of purified *Af*OYE1. The reaction mixtures in 5 mL potassium phosphate buffer (100 mM, pH 7.0), were conducted at 25–60 °C for 10 min, or in 5 mL potassium phosphate buffer (100 mM, pH 5.5–8.0) were conducted at 45 °C for 10 min. Then, the mixtures were extracted with ethyl acetate (1 mL), and the yield of cyclopentanone was determined by GC with an external standard calibration curve.

Preparative biotransformation was carried out in 50 mL potassium phosphate buffer (100 mM, pH 7.0), 0.5 μM purified *Af*OYE1, 20 U glucose dehydrogenase from *Bacillus*, 200 mM glucose, 20 mM NADH, and 50 mM substrate, and then the reactions were conducted at 45 °C for 10 h. Then, the mixtures were extracted with ethyl acetate (3 × 50 mL), the organic phase was combined and dried by anhydrous NaSO_4_, the solvents were removed under certain reduced pressure, and then the final product was purified with column chromatography and subjected to NMR and optical rotation analysis.

The kinetic parameters *K*_*m*_ and *k*_cat_ of *Af*OYE1 for 2-cyclopentenone were determined by measuring the production of cyclopentanone. The reaction mixture containing varying concentrations of 2-cyclopentenone in 5 mL potassium phosphate buffer (100 mM, pH 7.0) was incubated under the optimal reaction conditions (pH 7.0, and 45 °C) at 220 rpm for 2.0 min. The mixtures were extracted with ethyl acetate (1 mL), and then the yield of cyclopentanone was determined by GC. The parameters were calculated using the Prism program (GraphPad, San Diego, CA, USA).

### Determination of the flavin cofactor

The flavin content of *Af*OYE1 was determined spectrophotometrically from absorption scans (350–750 nm) using a SpectraMax® M2e (Molecular Devices, USA) microplate reader. Free flavin was released from *Af*OYE1 by the protein denaturation through incubating in a boiling water bath for 10 min; then, the samples were centrifuged at 15,000 g for 10 min. The supernatant, *Af*OYE1 solution, and standard flavin mononucleotide (FMN) solution were analyzed by microplate readers to determine flavin content.

### Crystallization

Initial crystallization screening of *Af*OYE1 (20 mg/mL) was carried out at 18 °C with high-throughput sparse matrix crystallization trails using a Gryphon crystallization robot (Art Robbins Instruments). MRC 96-well two-drop standard plates were adopted with the sitting-drop vapor diffusion method by mixing 0.5 μL protein and 0.5 μL reservoir solution in the initial screening. A 24-well plate was used with the hanging-drop vapor diffusion method by mixing 1 μL protein and 1 μL reservoir solution in manual optimization steps. Well-diffracted crystals were achieved in the condition containing 0.1 M Bis–Tris propane, pH 6.5, 15% (w/v) PEG 3350, and 0.2 M sodium nitrate, after seeding with smaller crystals initially obtained in the same condition. Crystals were cryoprotected in mother liquor supplemented with 25% (v/v) ethylene glycerol and snap-frozen in liquid nitrogen for data collection.

### Data collection and structural determination

The X-ray diffraction data were collected at 100 K Crystallography Beamline 18U1 (BL18U1) using a Pilatus 3 S 6 M detector at Shanghai Synchrotron Radiation Facility (SSRF, Shanghai, China) and at the wavelength of 0.979 Å. Datasets were processed using XDS (Kabsch 2010). The structure was solved by molecular replacement using a modified monomer model of ene-reductase (PDB code: 5OCS, sharing 42% identity with *Af*OYE1) (Opperman 2017) with all amino acids mutating to alanine in coot as a search model. An *Af*OYE1 dimer was positioned in the asymmetric unit using PHENIX Phaser-MR (McCoy et al. 2007). All refinement and model-building procedures were carried out by PHENIX.refine (Adams et al. 2010) and COOT (Emsley and Cowtan 2004). FMN was automatically imported from the Coot dictionary. The final parameters obtained for the best models reached a Rfactor/Rfree of 0.144/0.167 at 1.55 Å (Table S1). All the structure figures were prepared by PyMOL.

### Phylogenetic analysis

The sequence alignment of 83 known OYEs (Table S2) and *Af*OYE1 was done by ClustalW, and then phylogenetic analysis was conducted using the Maximum Likelihood statistical method based on the Jones-Taylar-Thornton (JTT) model in MEGAX. The initial tree for the heuristic search was obtained by applying NJ/BioNJ algorithms to a matrix of pairwise distance that was estimated by a *p*-distance and then selecting the topology with superior log likelihood value (− 28,266.79). The visualizing phylogenetic tree was produced by an online tree viewer iTOL Version 6.5.8, and the assignment of the subclasses was based on a previous evolutionary study.

### Construction of point mutations

The mutants were constructed by site-directed mutagenesis using primers listed in Table S3 and confirmed by sequencing (Shenggong, Shanghai, China). The correct plasmid was transformed into *E. coli* BL21 for protein production.

## Results

### Mining OYEs from A. flavus and sequence analysis

To identify new OYEs from *A. flavus*, the thermophilic-like OYE YqjM from *Bacillus subtilis* was used as a reference to search the *A. flavus* proteins in UniProtKB/Swiss-Prot database. The returned results showed that *A. flavus* contains two OYEs, including a putative NADPH dehydrogenase afvA (UniProt: B8N8Q9) and an NADP-dependent oxidoreductase lnbE (UniProt: B8NWW6). The afvA and lnbE have 41.7% and 34% identities with YqjM, respectively. Here, the afvA was selected since it has higher identities with the known YqjM, and then it was overexpressed in *E. coli* for further analysis. Functional analysis showed that afvA has the ability to reduce 2-cyclopentenone into 2-cyclopentanone. The *Af*OYE1 was previously named afvA, which was annotated as an NADPH dehydrogenase. afvA was proposed to catalyze the hydroxylation of siderin and 7-demethyl siderin to the corresponding hydroxylsiderin and hydroxydemethylsiderin (Uka et al. 2020). However, we found that afvA could not hydroxylate the siderin analogs, such as 6-hydroxy-4-methylcoumarin, 6-methylcoumarin, and 7-methylcoumarin, which indicated that afvA might not be able to catalyze the hydroxylation. Here, we annotated afvA as *Af*OYE1 based on the functional analysis.

**Fig. 1:**
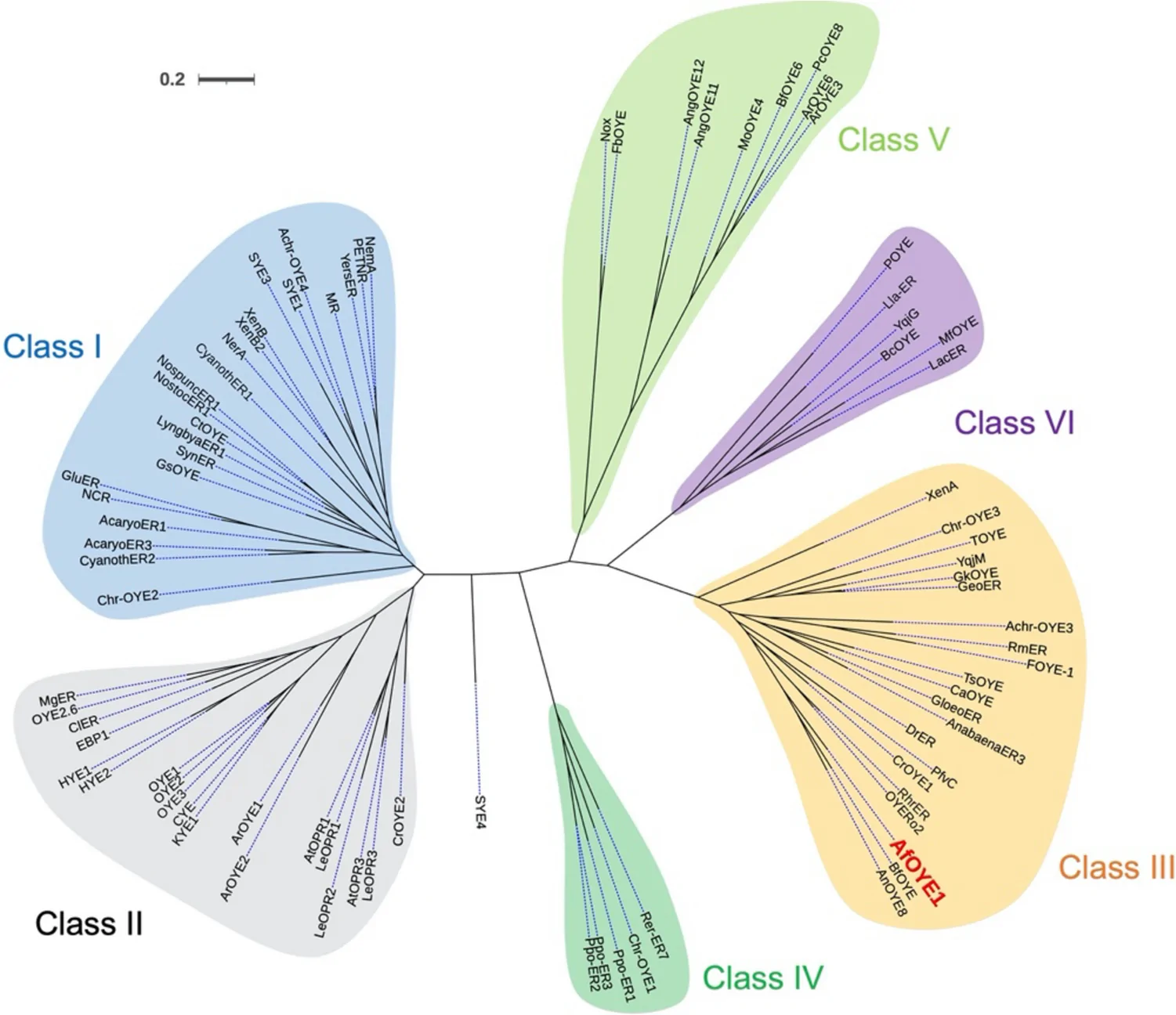
Phylogenetic analysis of *Af*OYE1 and previously described OYEs. The phylogenetic analysis was conducted using the Maximum Likelihood statistical method based on the Jones-Taylar-Thornton (JTT) model in MEGAX. The corresponding alignment was done by ClustalW. The accession numbers of the previously described OYEs are listed in Table S2

The phylogenetic analysis of *Af*OYE1 and 83 known OYEs was conducted, and the results showed that *Af*OYE1 belongs to the group of Class III OYEs (previous thermophilic-like OYEs group) (Fig. 1) (Peters et al. 2019). The sequence alignments showed that *Af*OYE1 conserved the two fingerprint motifs of the thermophilic-like OYE homologs and the catalytic residues of OYEs, including His211, His214, and Tyr216 (Fig. S1) (Kitzing et al. 2005). *Af*OYE1 has the highest similarities to the OYEs from *Botryotinia fuckeliana* (*Bf*OYE4) and *Aspergillus niger* (*An*OYE8), with 53.6% and 51.7% identities, respectively (Robescu et al. 2022a). The phylogenetic analysis and sequence properties also indicated that *Af*OYE1 is an ene-reductase other than a hydroxylase.

### Expression and characterization of the recombinant

The heterologous expression of *Af*OYE1 with an N-terminal His6-tag was conducted in the host *E. coli* BL21 (DE3) at 16 °C. SDS-PAGE analysis showed that *Af*OYE1 was produced with a molecular mass of around 43 kDa (**Fig. S2**), which was in accordance with the predicted molecular mass derived from the amino acid sequence.

**Fig. 2:**
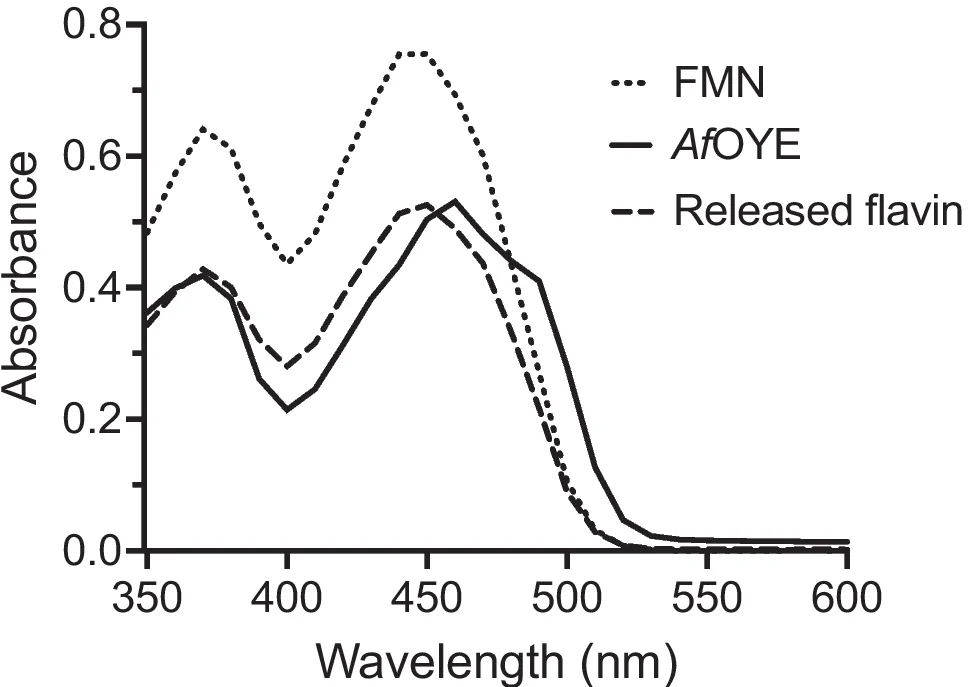
UV–visible absorption spectra of purified *Af*OYE1 (solid line), released flavin after thermal denaturation (100 °C for 10 min, dotted line), and standard FMN (dash line)

The UV–VIS absorption spectra of the purified *Af*OYE1, released flavin, and standard FMN suggested that *Af*OYE1 contained an FMN molecule as its prosthetic group and the FMN was non-covalently bound to the protein (Fig. 2). The supernatant after boiling of *Af*OYE1 and centrifugation turned into bright yellow, and both the released flavin and standard FMN had identical spectra, which had maxima absorbance at about 370 and 450 nm. The flavin contained *Af*OYE1 exhibited maxima absorbance at 370 and 460 nm.

### Evaluation of the thermostability of AfOYE1 and optimal reaction conditions

**Fig. 3:**
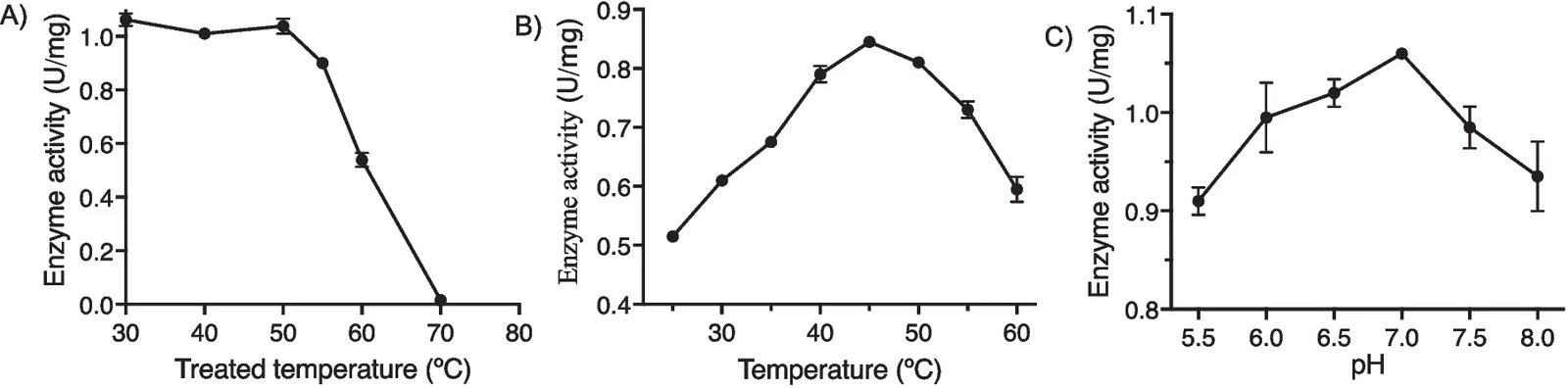
Thermostability **(A)**, temperature **(B)**, and pH **(C)** optima of *Af*OYE1. The standard deviations of triplicates are represented by error bars

The *Af*OYE1 thermal stability was evaluated by detecting the midpoint for thermal inactivation (*T*_1/2_). The irreversible thermal deactivation curve showed that the *T*_1/2_ of *Af*OYE1 was about 60 °C (Fig. 3A). Moreover, the catalytic activity of *Af*OYE1 decreased slowly after 5 min heat treatment at the temperature below 55 °C, and then a drastic decline was observed at the temperature above 55 °C.

To further characterize *Af*OYE1, the effects of temperature and pH on its catalytic activity with 2-cyclopentenone were investigated carefully. *Af*OYE1 exhibited high catalytic activities at temperatures 40–50 °C and observed maximum activity (0.85 U/mg) at 45 °C (Fig. 3B). The data showed that *Af*OYE1 still conserved 70% of the maximal activity at 60 °C (0.6 U/mg) and over 60% of the maximal activity when the temperature was down to 20 °C (0.52 U/mg). Moreover, *Af*OYE1 had high catalytic activity in a wide range of pH (pH 5.5–8.0) (Fig. 3C). The highest catalytic activity (1.06 U/mg) of *Af*OYE1 was observed at pH 7.0, and it displayed over 85% of the maximal activity at pH 5.5 (0.91 U/mg) and pH 8.0 (0.94 U/mg).

### Evaluation of the catalytic activity of AfOYE1 toward activated alkenes and kinetic analysis

**Fig. 4:**
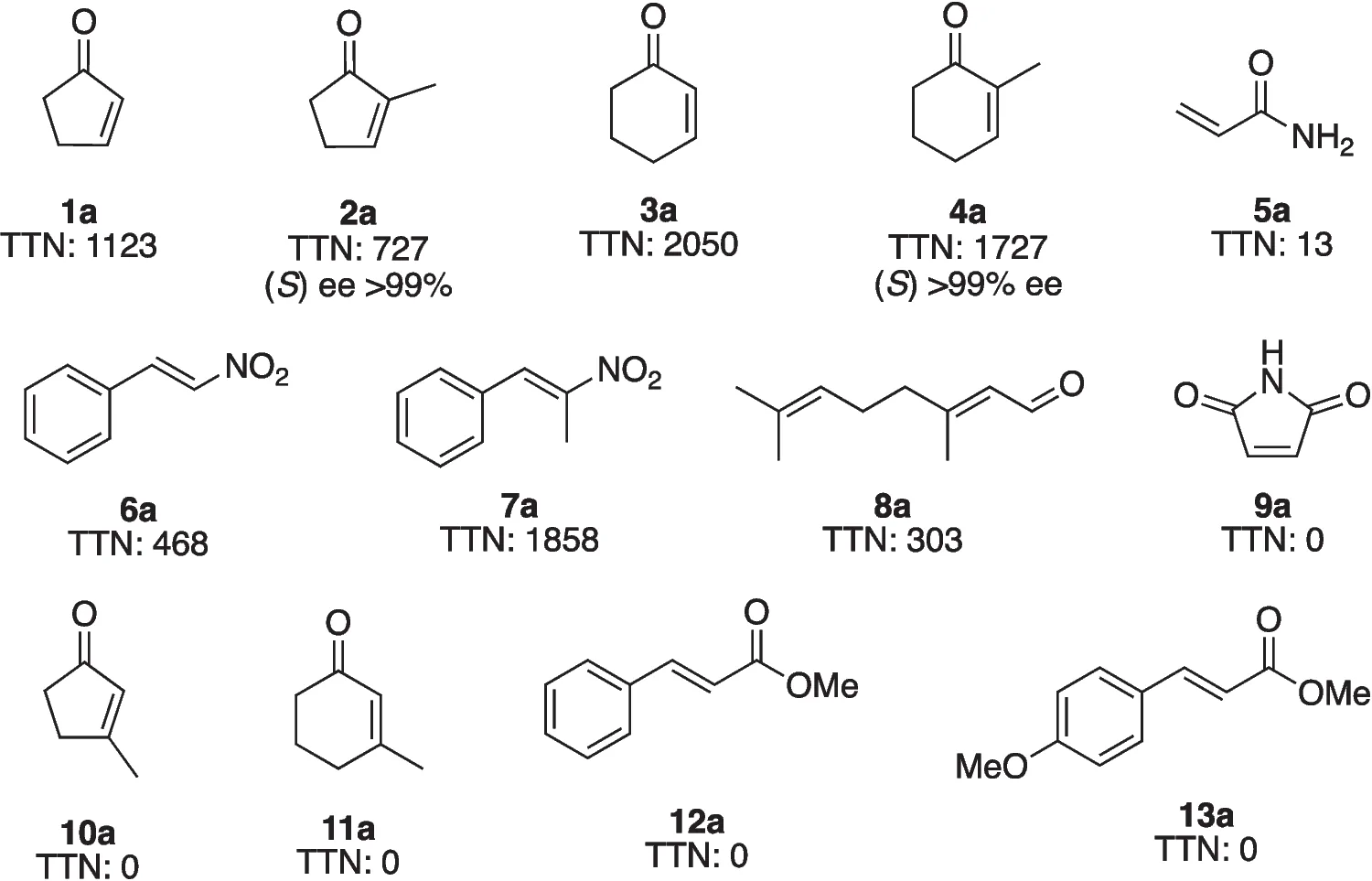
*Af*OYE1-catalyzed reduction of activated α, β-unsaturated alkenes. Reaction conditions: 5 mL PBS (100 mM, pH 7.0), 0.1 μM *Af*OYE1, 5 U glucose dehydrogenase, 50 mM glucose, 20 mM NADH, and 8 mM substrate, at 45 °C for 10 min. TTN (total turnover number): the total moles of product yield divided by the moles of *Af*OYE1

The *Af*OYE1-catalyzed bioreduction system was further applied to different kinds of activated α, β-unsaturated alkenes. The results showed that cyclic enones (**1**, **2**, **3**, and **4**), acrylamide (**5**), nitroalkenes (**6** and **7**), and α, β-unsaturated aldehydes (**8**) could be reduced by *Af*OYE1 (Fig. 4). However, cyclic imide (**9**) and α, β-unsaturated esters (**12** and **13**) could not be accepted as substrates for *Af*OYE1. *Af*OYE1 displayed the highest activity toward 2-cyclohexenone with TTN of 2050. For the *Af*OYE1-catalyzed bioreduction of cyclic enones, it had higher activity toward cyclohexenones than cyclopentenones (**3** vs. **1**, **4** vs. **2**). Similar to other OYEs in the thermophilic-like group, *Af*OYE1 was shown to have a restricted substrate spectrum (Amato and Stewart 2015).

The results showed that the substituent on the cyclic moiety had an important effect on the catalytic activity. *Af*OYE1 could catalyze the reduction of α-methyl substituted cyclopentenone (**2**) and cyclohexenone (**4**). However, it could not convert the β-methyl substituted cyclopentenone (**10**) and cyclohexenone (**11**) into the corresponding product. Moreover, the *Af*OYE1-catalyzed reduction had excellent enantioselectivities toward the prochiral alkenes. The bioreduction of 2-methylcyclopentenone (**2**) and 2-methylcyclohexenone (**4**) yielded the corresponding (*S*)-2-methylcyclopentanone ([α]^25^_D_ =  + 16.5° (c 0.4, CHCl_3_) ((*S*), [α]^25^_D_ =  + 114.9° (c 0.52, CHCl_3_) (Shimoda et al. 2004)) and (*S*)-2-methylcyclohexanone with > 99% ee.

The kinetic parameters *K*_*m*_ and *k*_cat_ of *Af*OYE1 were investigated with varying concentrations of 2-cyclopentenone (Fig. S3). The *K*_*m*_ value determined by non-linear regression was 0.061 ± 0.005 mM^−1^, which is significantly smaller than those of most of the known OYEs (Robescu et al. 2022b, 2020; Xu et al. 2014). The apparent *k*_cat_ of the *Af*OYE1 on 2-cyclopentenone was 34.12 ± 0.74 min^−1^, resulting in catalytic efficiencies of 9.32 mM^−1^ s^−1^.

### Crystal structure of AfOYE1

**Fig. 5:**
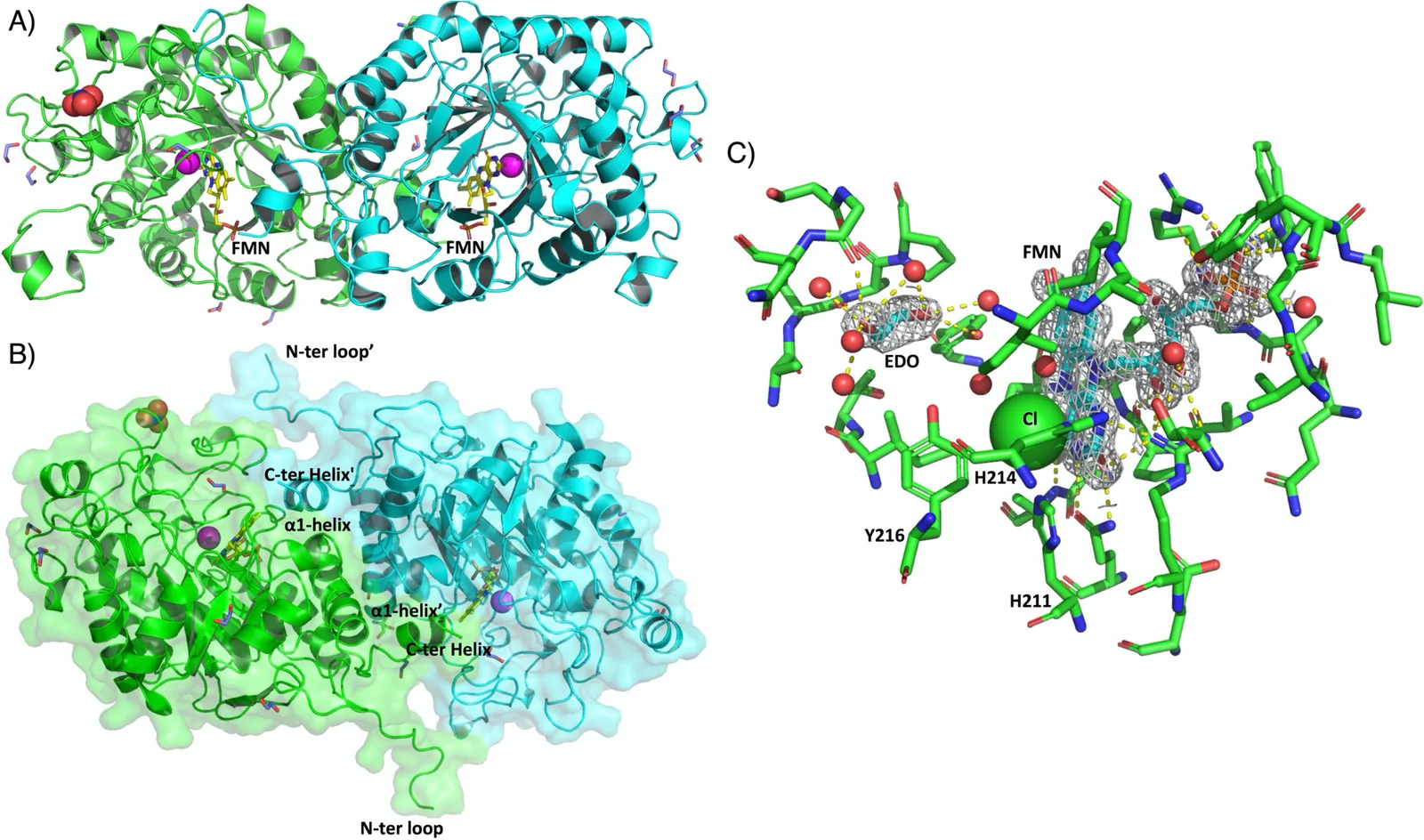
Crystal structure of *Af*OYE1. **A** Overall cartoon structure of *Af*OYE1. The two protomers of *Af*OYE1 are colored green and cyan, with FMN (yellow) and Cl (magenta) buried in each protomer’s active pocket. **B** Surface representation showing the two protein chains forming the dimeric architecture contributed deeply by the interaction of α1-helix, N-terminal loops, and C-terminal helix of each protomer. **C** Details of *Af*OYE1 catalytic cavity. The omit |*F*_*o*_|-|*F*_*c*_| map is shown in gray mesh contoured at 2σ level indicating locations of FMN and ethylene glycol (EDO). FMN and EDO bound in the active site are shown with C atoms in cyan and Cl in the green sphere together with H_2_O in the red dot. Relevant residues of the catalytic cavity are shown with green C atoms, Red O, and Blue N. Hydrogen bonds are shown as yellow dashed lines

The structure of *Af*OYE1 (PDB: 8J59) was determined to have a maximum resolution of 1.55 Å in the P1211 space group with an elongated dimer per asymmetric unit (Table S2). The overall structure of *Af*OYE1 showed an expected eight-stranded (α, β)-barrel fold of triosephosphate isomerase (TIM), which is highly conserved in the thermophilic-like OYEs (Fig. 5A). On the top of the β-barrel core of each monomer encircles one FMN cofactor, which points toward the C-terminal side and is buried within the active site cavity. Similar to other thermophilic-like OYEs, the PDBePISA analysis (https://www.ebi.ac.uk/msd-srv/prot_int/cgi-bin/piserver) of the *Af*OYE1 structure showed that the inverse dimeric architecture with an interacting surface of 492 Å^2^ is contributed by α1-helix, the peculiar long N-terminal loops, and the C-terminal helix of each protomer, which contains 7 hydrogen bonds and 6 salt bridges among the 41 interfacing residues (Fig. 5B and Table S4) (Krissinel and Henrick 2007). Meanwhile, *Af*OYE1 has a slightly negative surface but a highly positive inner surface at the entrance of the active site, which endows its ability to bind the negatively charged FMN cofactor (Fig. S4).

The DoGSiteScorer analysis (https://proteins.plus) of the structure showed that the catalytic cavity of *Af*OYE1 is quite deep, and the FMN cofactor is heavily buried at the bottom of the catalytic hole with a depth of 24.23 Å (Table S4). As shown by the difference electron density map *F*_*o*_-*F*_*c*_, the *si*-face of the FMN faced the solvent, while the *re*-side was in contact with the protein backbone tightly via the extensive hydrogen bonding and hydrophobic interactions with the side chain and main chain elements (Fig. 5C and Fig. S5) (Robescu et al. 2022b). Meanwhile, the histidine pair (His211/His214) together with the highly conserved proton donor Tyr216 interacts with a buffer-sourced chloride anion and is positioned on the *si*-face of the isoalloxazine ring of FMN. It indicated that His/His together with Tyr 216 participated in the binding and correct orientation of substrate (Fig. 5C) (Robescu et al. 2022b, 2020, 2021).

Until now, two OYE structures from filamentous fungi, including OYE4 from *B. fuckeliana* (*Bf*OYE4, PDB: 7BLF) (Robescu et al. 2022a) and OYE from *A. niger* (*An*OYE8, PDB: 7QFX) (Robescu et al. 2022b), have been reported. The superposition structures of *Af*OYE1 with *Bf*OYE4 and *An*OYE8, with an r.m.s.d. of 0.694 Å and 0.588 Å, respectively, showed that they have similar monomer folding, except the α6-helix for the residues (from Phe271 to Glu280), the loop region (from Ser316 to Gly325), and the cap subdomain region (from Ala132 to Thr182) (Fig. S6). The protruding α6-helix and the long N-terminal loops of *Af*OYE1 work dimerization function in multi-asymmetry units (Fig. S7). However, the protruding α6-helix shows random loops in other OYEs (Fig. S6). The loop region from Ser316 to Gly325 runs on the enzyme surface and reaches the active site entrance, which contributes to the size and feature of the catalytic cavity (Fig. S8A). The residues Ile318 and Ile320 in the loop have a distance to the FMN flavin group of 5.1 Å and 4.1 Å, respectively. Meanwhile, the residue Ile320 points its side chain toward the top of the catalytic cavity, which is stabilized by a hydrogen bond with Arg371 at the C-terminal α-helix (from Arg371 to Gln375) (Fig. S8B). The cap region of *Af*OYE1 with large unstructured turns is located on the top of the entrance of the active site (from Ala132 to Thr182), while the *Bf*OYE4 shows α-helices and the *Gs*OYE (PDB: 6S0G) shows β-hairpins (Robescu et al. 2020) (Fig. S8C), which may involve interacting with NADH/NADPH and adjusting the selectivity toward FMN cofactor (Adalbjornsson et al. 2010; Knaus et al. 2016; Pompeu et al. 2012; Pudney et al. 2007). The C-terminal α helix of each monomer (from Thr395 to Phe401) points to the active sites of the adjacent monomer, and the “finger” residue Trp399 interacts with the respective FMN flavin group at a distance of 3.8 Å (Fig. S8B). This may play an important role in enzyme assembling, TIM barrel catalytic domain surface stabling, and two protomers dimerization (Fig. S8B).

### Analysis of the catalytic residues of AfOYE1

**Fig. 6:**
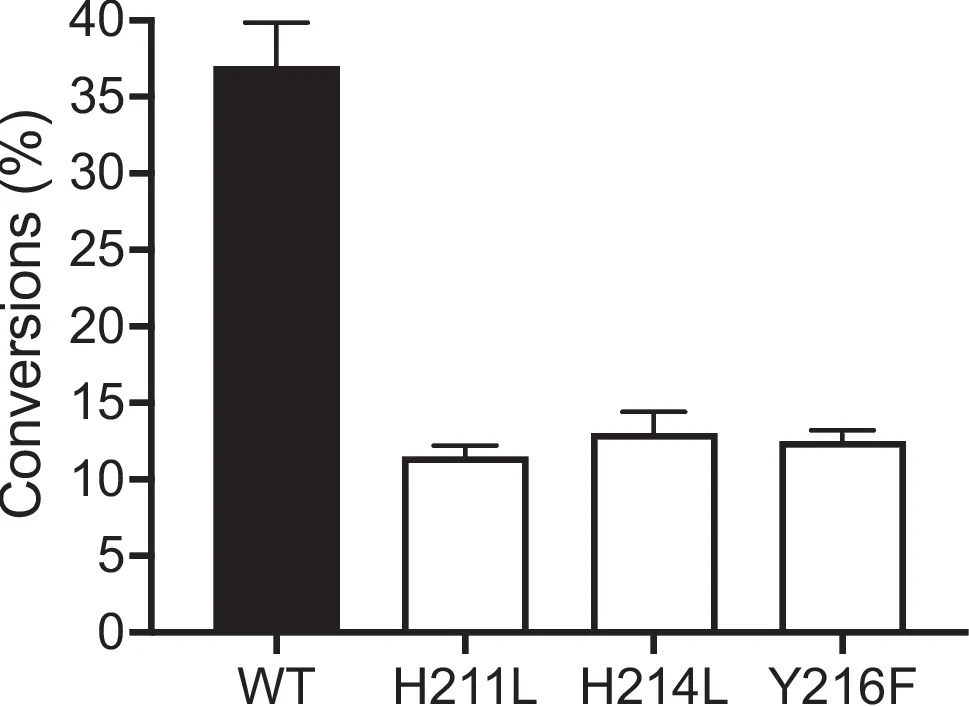
The catalytic activity of the variants and wild type *Af*OYE1 toward 2-cyclopentenone

Both the sequence alignment and structure analysis indicated that *Af*OYE1 highly conserved the catalytic residues, including His211, His214, and Tyr216. Here, the function of those residues was investigated. Both His211 and His214 were mutated into leucine, creating the variants H211L and H214L. The catalytic activity analysis showed that these two variants still conserved about 30% of catalytic activity of the wild type *Af*OYE1 (Fig. 6). Previously, the histidine pair has been considered as the binding motifs of OYE homologs, such as His164 and His167 in YqjM (Shi et al. 2020), His172 and His175 in Crs (Opperman et al. 2010), and His182 and Asn185 in “thermophilic-like” OYE homologs (Riedel et al. 2015). The variants H211L and H214L had only 30% catalytic activity of the wild type, which indicated that the histidine residues in the catalytic center were important for substrate binding. Furthermore, the tyrosine residue in the catalytic center of OYE was proposed as a proton donor of OYE, which contributed to the transfer of proton to substrate at the α-position (Kitzing et al. 2005). Therefore, Tyr216 was mutated to phenylalanine (Y216F) to investigate whether Tyr216 serves as the proton donor in *Af*OYE1. The results showed that the variant Y216F conserved 30% catalytic activity of the wild type (Fig. 6), which indicated that Tyr216 did act as a proton donor in *Af*OYE1. However, alternative proton donors might exist, because the variant Y216F was still able to reduce 2-cyclopentenone.

## Discussion

The *Af*OYE1 was previously named afvA, which was annotated as an NADPH dehydrogenase. The proposed function of afvA was to hydroxylate siderin and 7-demethyl siderin in the biosynthesis of aflavarin (Cary et al. 2015). Here, the recombinant *Af*OYE1 was shown to have the ability to reduce the activated alkenes. However, it could not catalyze the hydroxylation of the siderin analogs, such as 6-hydroxy-4-methylcoumarin, 6-methylcoumarin, and 7-methylcoumarin. These data indicated that *Af*OYE1 might not play as a hydroxylase in the biosynthesis of aflavarin. Similar to other OYEs in the thermophilic-like group, the recombinant *Af*OYE1 was shown to have a restricted substrate spectrum (Amato and Stewart 2015). It has the catalytic activity toward cyclic enones, acrylamide, nitroalkenes, and α, β-unsaturated aldehydes, but the cyclic imide and α, β-unsaturated esters could not be accepted as substrate.

Until now, over twenty OYE homologs from Class III OYEs have been identified, including *Bf*OYE4 from filamentous fungus *B. fuckeliana* and *An*OYE8 from filamentous fungus *A. niger*. Although *Bf*OYE4 and *An*OYE8 were phylogenetically clustered in the group of Class III OYEs, the thermostability of these two filamentous fungi source OYEs is similar to those of non-thermostable OYEs (Robescu et al. 2022b). This newly identified *Af*OYE1 from filamentous fungus *A. flavus* had high catalytic activity at 40–50 °C and with the optima temperature at 45 °C. Moreover, the thermal stability analysis showed that the midpoint for thermal inactivation (*T*_1/2_) of *Af*OYE1 was 60 °C. All the data indicated that *Af*OYE1 is a thermostable OYE. Therefore, the first thermostable OYE is identified from *A. flavus*, which provides a new optional thermostable OYE in asymmetric reduction of activated alkenes.

*Af*OYE1 catalyzed the bioreduction of 2-methylcyclopentenone and 2-methylcyclohexenone with excellent (*S*)-enantioselectivity, producing the corresponding (*S*)-2-methylcyclopentanone and (*S*)-2-methylcyclohexanone with > 99% ee. Until now, most of the known OYEs are (*R*)-stereoselectivity, and only 4 OYEs, including KYE1 from *Kluyveromyces lactis* (Yanto et al. 2011), YersER from *Yersinia bercovieri* (Yanto et al. 2011), *Ct*OYE from *Chroococcidiopsis thermalis* (Yanto et al. 2011), and *Gs*OYE from *Galdieria sulphuraria* (Robescu et al. 2020), were (*S*)-stereoselectivity in the reduction of 2-methylcyclohexenone to 2-methylcyclohexanone. However, *Af*OYE1 only shared 23–33% of identities with those four OYEs. The identification of a new thermostable *Af*OYE1 would provide a robust enzyme for (*S*)-enantioselective bioreductions.

The novel structure of the loop of Ser316 to Gly325 narrows the entrance of the catalytic pocket. This 10aa-long loop of *Af*OYE1 spans the entrance of the catalytic pocket and bumps close to the entrance of the catalytic pocket, resulting in a narrow entrance of the catalytic center (Fig. S9A). However, the corresponding loop in *Ca*OYE (Ser265 to Try280), *An*OYE8 (Ser308 to Phe323), *Bf*OYE4 (Ser324 to Tyr339), YqjM (His 255 to Pro262), *Ts*OYE (Ser260 to Val277), and *Gk*OYE (Ser250 to Gln265) was away from the entrance of the catalytic pocket (Fig. S9A), resulting in an open catalytic pocket (Robescu et al. 2022b, 2021). Meanwhile, similar to the residue Arg325 of *Ca*OYE and Phe368 of *Rm*ER, the “finger” residue Trp399 of *Af*OYE1, which is located in the C-terminal α helix of each monomer and points to the FMN flavin group, and together with the cap region (residues Ala132 to Thr182) may interact with NADH/NADPH and adjust the selectivity of FMN cofactor (Opperman 2017).

The functional analysis of the conserved catalytic residues His211, His214, and Tyr216 of *Af*OYE1 showed that all three residues were important to its catalytic activity. However, the mutagenesis of those three residues did not eliminate their catalytic activity. The histidine pair has been considered as the binding motifs of OYE homologs, such as His164 and His167 in YqjM (Shi et al. 2020), His172 and His175 in Crs (Opperman et al. 2010), and His182 and Asn185 in “thermophilic-like” OYE homologs (Riedel et al. 2015). The *Af*OYE1 variants H211L and H214L conserved 30% catalytic activity of the wild type, which showed that those two histidine residues played an important role in the substrate binding. Meanwhile, the variants H211L and H214L still exhibited activity, which indicated that other residues in the catalytic center of *Af*OYE1 might enable the enzyme to bind the substrate when the histidine was replaced. The replacement of Tyr216 by phenylalanine resulted in a dramatic decrease in catalytic activity, indicating Tyr216 acts as a proton donor in the *Af*OYE1 (Kitzing et al. 2005). Thus, a common catalytic mechanism can be anticipated for *Af*OYE1 and other OYEs. On the other hand, the variant Y216F could still reduce 2-cyclopentenone into 2-cyclopentanone, which suggested that the proton transfer process could be compensated. Previously, it has also been shown that the replacement of Tyr177 in CrS resulted in the variant only harboring 20% of the catalytic efficiency (Opperman et al. 2010).

In summary, a new thermophilic-like OYE *Af*OYE1 was identified from *A. flavus*. It could reduce a spectrum of activated alkenes, had an optimal temperature of 45 °C, and exhibited high reduction activity in a wide pH range (pH 5.5–8.0). Unlike most of the known OYEs, the *Af*OYE1-catalyzed asymmetric reduction had (*S*)-selective with excellent enantioselectivity, which expands the toolbox of thermostable (*S*)-selective OYEs. The crystal structure of *Af*OYE1 revealed that it had a special catalytic cavity, which may be relevant to its enantioselectivity. Our results indicate that fungi are good sources for the identification of new OYEs. We believe the newly identified *Af*OYE1 will give an alternative for asymmetric alkene hydrogenation in industrial processes.

## Supplementary Information

Below is the link to the electronic supplementary material.

Supplementary file1 (PDF 22613 KB)

## Data Availability

All data generated or analyzed during this study are included in this published article (and its supplementary information files).
