# Supplementary material for: Structural and functional characterization of a new thermophilic-like OYE from Aspergillus flavus
Source: Appl Microbiol Biotechnol. 2024 Jan 13;108:134. doi: 10.1007/s00253-023-12963-w (PMC10787880; doi:10.1007/s00253-023-12963-w)
Supplement: Supplementary file 1 [file 253_2023_12963_MOESM1_ESM.pdf]

Structural and Functional Characterization of a New Thermophilic-like OYE from *Aspergillus flavus*

Na Li[1#], Yuan Wang[1#], Yinyin Meng[2], Yangyong Lv[1], Shuaibing Zhang[1], Shan Wei[1], Pingan Ma, Yuansen Hu[1*], Hui Lin[2*]

[1.] College of Biological Engineering, Henan University of Technology, 100 Lianhua Street, Zhengzhou, 450001, Henan, China.

[2.] Henan International Joint Laboratory of Biocatalysis and Bio-based Products, College of Life Sciences, Henan Agricultural University, 95 Wenhua Road, Zhengzhou 450002, China.

[#] These authors contributed equally to the work.

Corresponding authors: Yuansen Hu (Email: hys308@126.com); Hui Lin (Email: huilin@henau.edu.cn)

Contents

1. **Figure S1.** Sequence alignment performed by Clustal Omega of the *Af*OYE1 from *Aspergillus flavus* with representative OYEs from different classes
2. **Figure S2**. SDS-PAGE analysis of the purified *Af*OYE1.
3. **Figure S3**. Steady-state kinetics of *Af*OYE1-catalyzed reduction of 2-cyclopentenone.
4. **Figure S4.** Surface charge distribution of *Af*OYE1.
5. **Figure S5.** The plot of FMN cofactor interactions in the actives site of *Af*OYE1.
6. **Figure S6.** Superposition of monomers from the crystal structures of *Af*OYE1, *Bf*OYE4, and *An*OYE8.
7. **Figure S7.** A view showing N-terminal loops and protruding α6-helix of *Af*OYE1 promote dimerization in five copes of crystallographically independent units.
8. **Figure S8.** Details of the loop region from Ser 316 to Gly 325, the entrance of the catalytic pocket, and the superposition of cap regions from the crystal structures of *Af*OYE1.
9. **Figure S9.** Surface representation of catalytic pocket among members of thermophilic-like Class III OYEs.
10. **Table S1**. Data collection and refinement statistics for *Af*OYE1.
11. **Table S2.** List of OYEs used for phylogenetic analysis and their accession numbers.
12. **Table S3**. Polymerase chain reaction primers for mutagenesis.
13. **Table S4**. Comparison of *Af*OYE1 characteristics with those of other thermophilic-like Class III OYEs.
14. The data of the products from the *Af*OYE1-catalyzed reduction.
15. Selected ^{1}H NMR spectra and GC chromatograms.
16. The sequence information of *Af*OYE1.

```
Nox           --------------------------MTTAPTPSIFEPARLG-PLTLRNRIVKAATFE--      31
YqiG          ---------------------------MNPKYKPLFEPFTFKSGVTIDNRIAVAPMTHYA      33
Chr-OYE1      -----------------------------MSTESLFTPFKYK-NLELKNRIVMAPMTRAQ      30
AfOYE1        MTQDIIDNIAAEGISYYTPAQVPPAGTQVEGSTKLFSPLTIR-GVTFPNRLFLAPLCQYS      59
YqjM          ------------------------------MARKLFTPITIK-DMTLKNRIVMSPMCMYS      29
PETNR         -----------------------------MSAEKLFTPLKVG-AVTAPNRVFMAPLTRLR      30
OYE1          ------------------MSFVKDFKPQALGDTNLFKPIKIG-NNELLHRAVIPPLTRMR      41
                                                :* *          :*

Nox           GVMPRGAVSDDL-INFHAEVARGGAAMTTVAYCAVSP--GGRVHRDTLVMDERALPGLRR      88
YqiG          SNEDGTISEAE--LDYIIPR-SKEMGMVITACANVTPD-GKAFPGQPAIHDDSNIPGLKK      89
Chr-OYE1      SD--NGVPTQQI-ADYYARRAAAEVGLILSEGTVINRPASKNMQNIPDFYGTEALNGWKN      87
AfOYE1        AK--DG-YANDWHLTHIGGIVQRGPGLAIMEATAVQKV-GRITPQDLGLYDDGHIEPLKR      115
YqjM          SHEKDG-KLTPFHMAHYISRAIGQVGLIIVEASAVNPQ-GRITDQDLGIWSDEHIEGFAK      87
PETNR         SIEPGDIPTPLM-GEYYRQRA--SAGLIISEATQISAQ-AKGYAGAPGLHSPEQIAAWKK      86
OYE1          ALHPGNIPNRDWAVEYYTQRAQRPGTMIITEGAFISPQ-AGGYDNAPGVWSEEQMVEWTK      100
              .       .           :       :    .     . .  :    .

Nox           LTDAVHAEGALAAAQIGHAGLVANTLSNK-----------------TKTLAPSTRL-SPP      130
YqiG          LAQAIQAQGAKAVVQIHHGGIECPSEL--VPQQ--------------DVVGPSDVF--DN      131
Chr-OYE1      VIDAVHHNGGKMGPQIWHVGDTRSTPD--YPL---------------------------E      118
AfOYE1        ITEFAHSQSQKIGIQLAHAGRKASAVAPWLSGNAMAVKEVG--GWPDDIVAPSAIPQEEG      173
YqjM          LTEQVKEQGSKIGIQLAHAGRKAELEGDIFA--------------------PSAIAFDEQ      127
PETNR         ITAGVHAEDGRIAVQLWHTGRISHSSI--QPGGQAPVSASALNANTRTSLRDENGNAIRV      144
OYE1          IFNAIHEKKSFVWVQLWVLGWAAFPDN--LARDGLRYDSA----SDNVFMDAEQEAKAKK      154
              :    : :      *:   *

Nox           AMGLVKGATLAELDGVVSDFERTARVAVDAGFDAIEVHLGHNYLLSSFMSPNLNKRHDRY      190
YqiG          GKQIARALKEEEVENIVKAFGEATRRAIEAGFDGVEIHGANGYLIQQFYSPKTNQRTDRW      191
Chr-OYE1      DMEKASTMTLEDIQDTIAQFAASAKSAKDLGFDVLEIHGAHGYLIDQFFWEGTNTRTDEY      178
AfOYE1        INAVPKVLTGEDIGVLKKDWAEAAKRAVRANFDAIEIHAAHGYLLHQFLSPVSNRRTDKY      233
YqjM          -SATPVEMSAEKVKETVQEFKQAAARAKEAGFDVIEIHAAHGYLIHEFLSPLSNHRTDEY      186
PETNR         DTTTPRALELDEIPGIVNDFRQAVANAREAGFDLVELHSAHGYLLHQFLSPSSNQRTDQY      204
OYE1          ANNPQHSLTKDEIKQYIKEYVQAAKNSIAAGADGVEIHSANGYLLNQFLDPHSNTRTDEY      214
                         .:       :  :.  :    . * :*:* .:.**: .*     * * *.:

Nox           GGS-VAKRAEYPRRVIEAVRVAAGSSV----AV------TAKFNMSDGVPKGLWLDQSLP      239
YqiG          GGS-DEKRLAFPLAIVDEVKKAASEHAKGAFLVGYRLSPEEPETPGLTMTET--------      242
Chr-OYE1      GGKTIKERSRFAVDVVKAIRAAVGED----FTIIIRLSQWKQQDYSVKLAHTP--EE---      229
AfOYE1        GGS-FENRVRILLEICEEVRAVIPTA----MPLLVRISATDWFEFDDNLTKEF--PESWT      286
YqjM          GGS-PENRYRFLREIIDEVKQVWD------GPLFVRVSASDYTDKG------------LD      227
PETNR         GGS-VENRARLVLEVVDAVCNEWSAD-----RIGIRVSPIGTFQNVDNGPNE------EA      252
OYE1          GGS-IENRARFTLEVVDALVEAIGHE-----KVGLRLSPYGVFNSMSGGAET------GI      262
              **.    :*       : . :                :

Nox           IAQILEADGHLDAMQLTGGSSLLNGMYFFRGEVPLAEFVASQPKLVGYGLKFYGPKIFPT      299
YqiG          ----YTLVDALGDK----------ELD---------YLHISLMDV------------NSK      267
Chr-OYE1      ---MEEWLLPLKDA----------GVD---------IFHCSQRRF------------WEP      255
AfOYE1        VAQSIRLALLLADR----------GVD---------LVDVSSGGI------------HAK      315
YqjM          IADHIGFAKWMKEQ----------GVD---------LIDCSSGAL------------VH-      255
PETNR         DALY--LIEELAKR----------GIA---------YLHMSETDL------------A--      277
OYE1          VAQYAYVAGELEKRAK-----AGKRLA---------FVHLVEPRV------------TN-      295
                        :              :            .        .

Nox           YPF----EEGFFLPFARQFRQALRMPLI-------------LLGGINRV----DTI-EHA      337
YqiG          AR---RGADP-TRTRMDLLNERVGNKVPL-----------IAVGSIHSA----DDALAVI      308
Chr-OYE1      ------EFEGSDLNFAGWAKKITGQPTITVGSVGLEGDFMAAFGGQGTEKADLTELTKRL      309
AfOYE1        SAIAIRSGPGYQVHFAQEIKKAVGEK-LL----------ISAVGGIKTG----ALAEEVV      360
YqjM          --ADINVFPGYQVSFAEKIREQA--D-MA----------TGAVGMITDG----SMAEEIL      296
PETNR         ------GGKPYSEAFRQKVRERFHG--VI----------IGA-GAYTAE----KAE-DLI      313
OYE1          -PFLTEGEGEYEGGSNDFVYSIWKG--PV----------IRA-GNFALH----PEVVREE      337
                                  .                       *

Nox           LDEGFEFVAMARALLRDPQLVNKFQAESVDQGLCIHCNKCMPTIYTGTRCVVRDALVVRE      397
YqiG          EN-GIPLVAMGREILVDPDWTVKVKEGREKQIETV--------IKGTD------KEKYHL      353
Chr-OYE1      ERGDFDLVAVGRALLQDPEWAKKVKEQNTEALLDF-------SAESLG------VL----      352
AfOYE1        QSGIDA-VQAGRWFQQNPGLVRAFANELGVKVRMATQ--IDWSFEGRG------KKAKKS      411
YqjM          QNGRADLIFIGRELLRDPFFARTAAKQLNTEIPAPVQ--YERGW----------------      338
PETNR         GKGLIDAVAFGRDYIANPDLVARLQKKAELNPQRP------ESFYGGG------AEGYTD      361
OYE1          VKDKRTLIGYGRFFISNPDLVDRLEKGLPLNKYDR------DTFYQMS------AHGYID      385
                      :  .*    :*  .

Nox           APRLGQ-------------      403
YqiG          PEPLWQAIVNTPGWVPYKD      372
Chr-OYE1      Y------------------      353
AfOYE1        --SL---------------      413
YqjM          -------------------      338
PETNR         YPSL---------------      365
OYE1          YPTYEE--ALKLGWDKK--      400
```

Figure S1. Sequence alignment performed by Clustal Omega of the *Af*OYE1 from *Aspergillus flavus* with representative OYEs from different classes: PETNR (Class I), OYE1 (Class II), YqjM (Class III), *Chr*-OYE1 (Class IV), Nox (Class V), and YqiG (Class VI). The catalytic residues are highlighted in blue, and the fingerprint motifs are highlighted in purple.

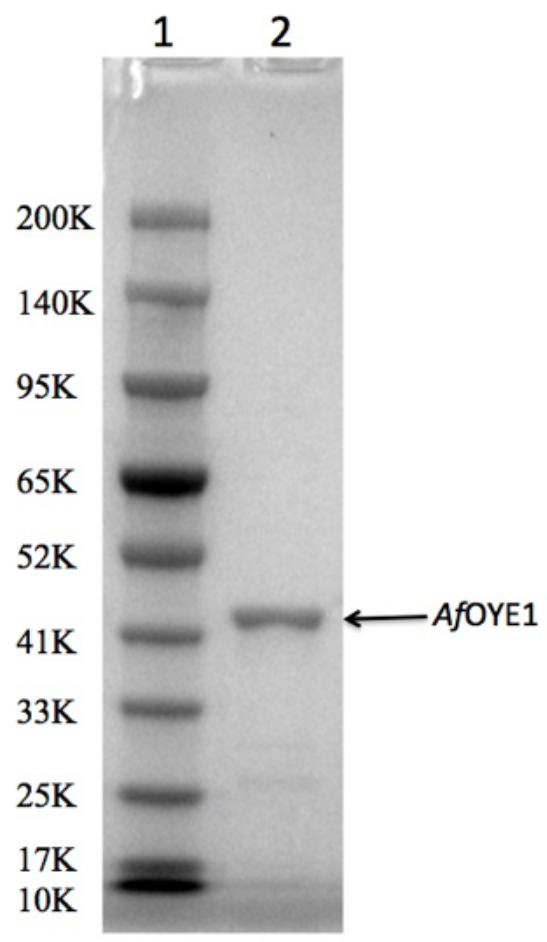

Figure S2. SDS-PAGE analysis of the purified AfOYE1. Lane 1: Protein ladder; Lane 2: The purified AfOYE1. The molecular weight of the purified AfOYE1 was at 43.4 KDa ranging between 41 KDa to 52 KDa. 10 uL of purified AfOYE1 was loaded on a 4-12% polyacrylamide gradient gel, and stained with Coomassie Blue.

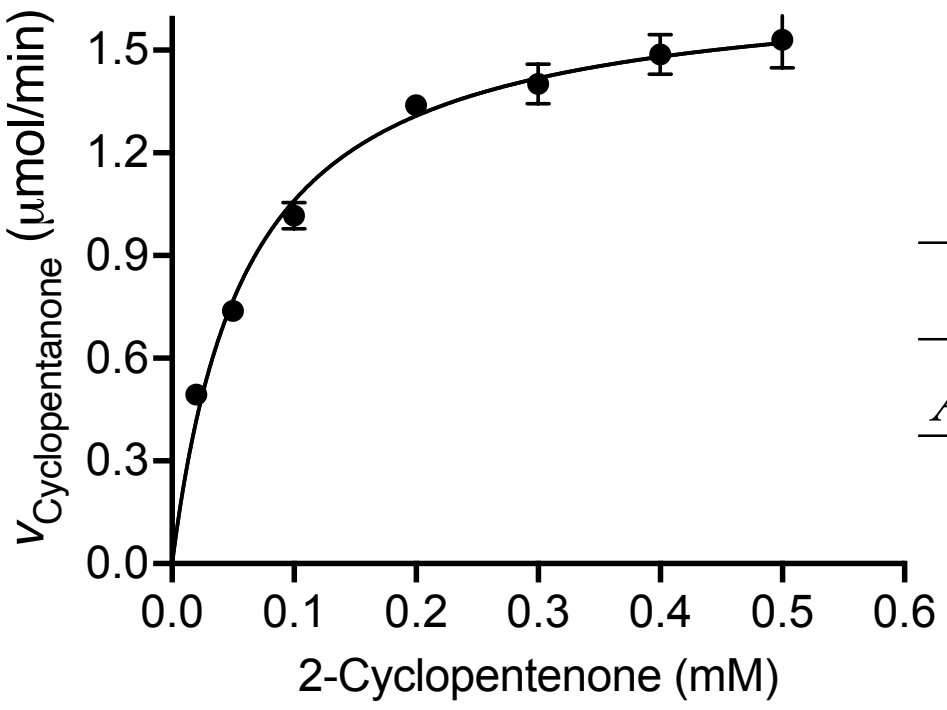

	K_m (mM^{-1})	k_{cat} (min^{-1})	k_{cat}/K_m ($mM^{-1}s^{-1}$)
*Af*OYE1	0.061 ± 0.005	34.12 ± 0.74	9.32

Figure S3. Steady-state kinetics of *Af*OYE1-catalyzed reduction of 2-cyclopentenone. Steady-state kinetics were measured in triplicates with varying concentrations of 2-cyclopentenone and fitting the data to the Michaelis-Menten equation. Errors limits are standard deviations.

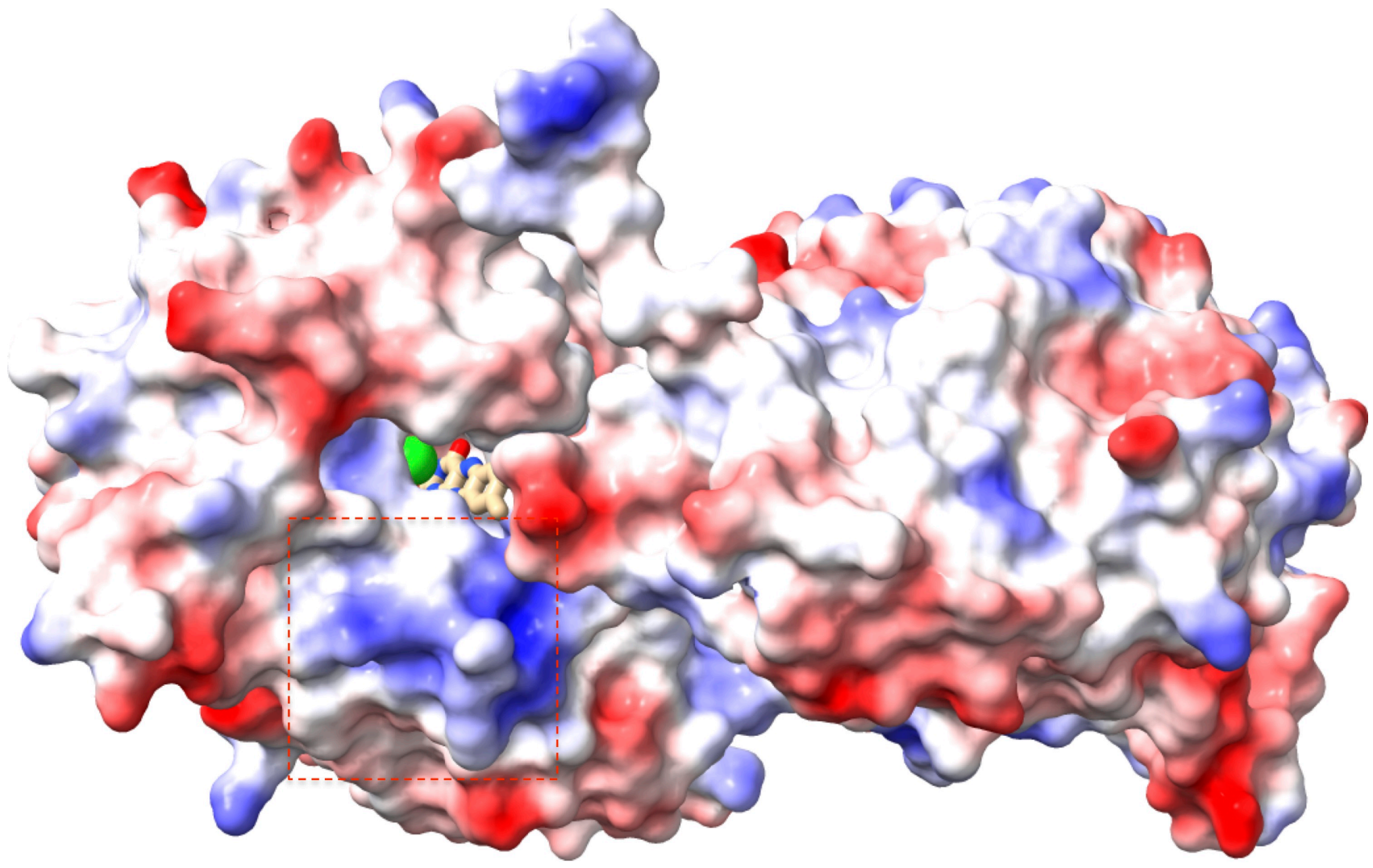

Figure S4. Surface charge distribution of *Af*OYE1, calculated by ChimeraX APBS software. Density with red potentially means negative, blue means positive, and white means neutral. Loop from S316 to G325 of *Af*OYE1 labeled as the red dashed rectangle is brimming with the positive surface.

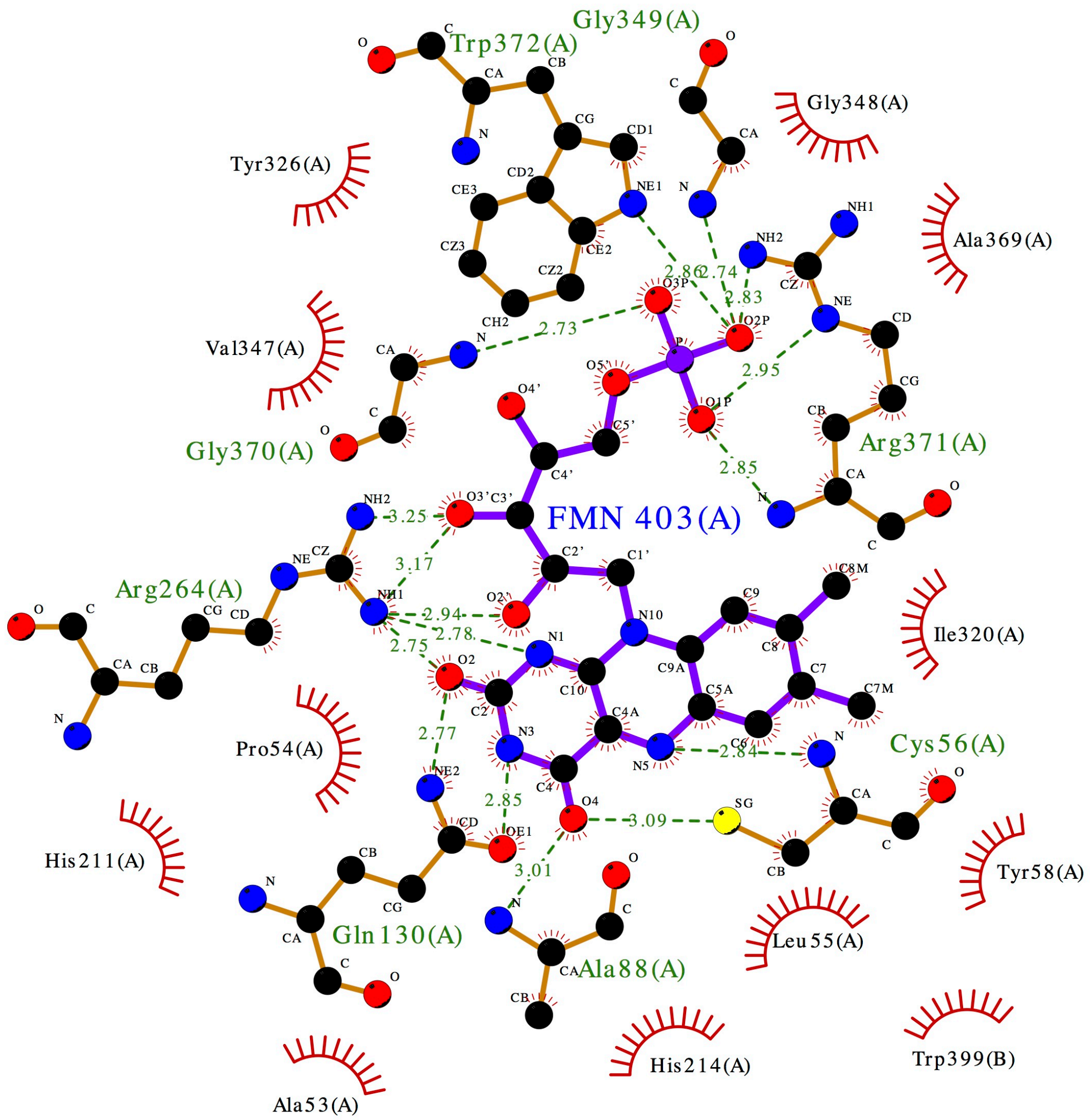

Figure S5. The plot of FMN cofactor interactions in the actives site of *Af*OYE1. The interactions have been calculated and visualized by LigPlus software. The thin green dotted lines illustrate hydrogen bonds.

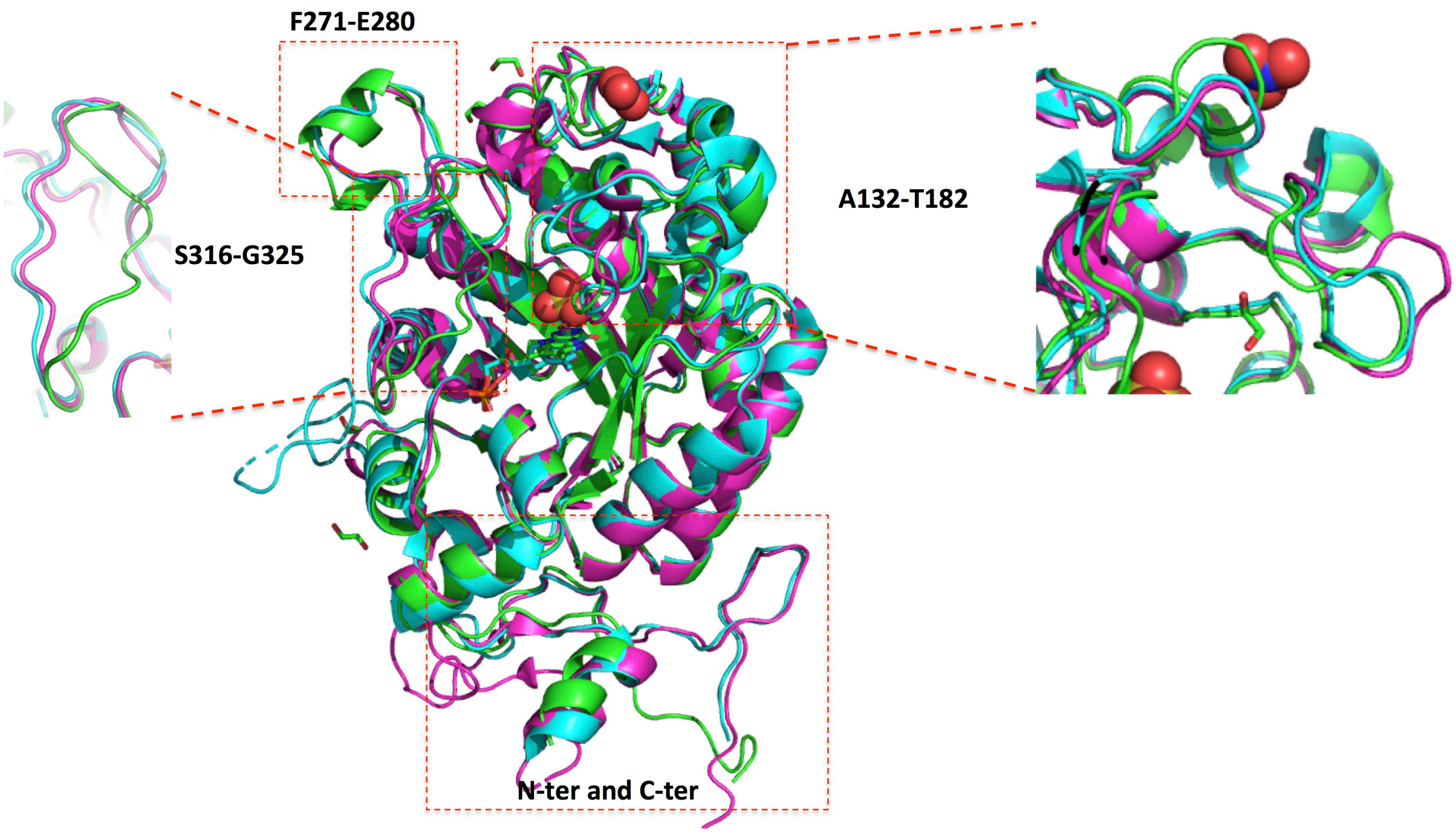

Figure S6. Superposition of monomers from the crystal structures of *Af*OYE1 (green), *Bf*OYE4 (cyan), and *An*OYE8 (magenta). Red dashed rectangle labeled as F271-E280 means α6-helix for the residues from Phe271 to Glu280 of *Af*OYE1, S316-G325 means the loop region from Ser316 to Gly325 of *Af*OYE1, A132-T182 means the cap subdomain region from Ala132 to Thr182, and N-ter and C-ter mean N terminal and C-terminal domains of *Af*OYE1.

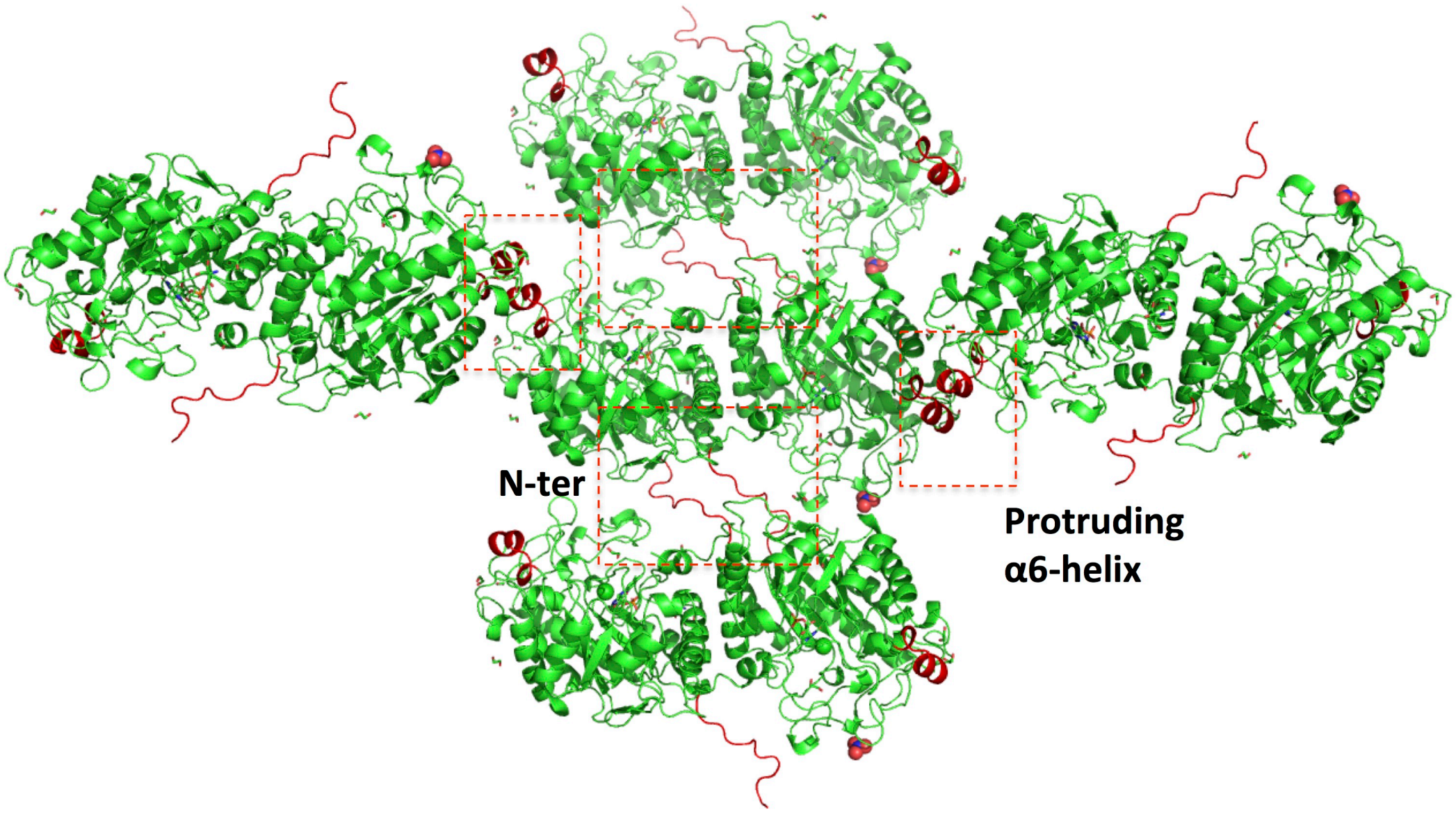

Figure S7. A view showing N-terminal loops (red) and protruding α6-helix (red color, residues F271 to E280) of *Af*OYE1 promote dimerization in five copes of crystallographically independent units.

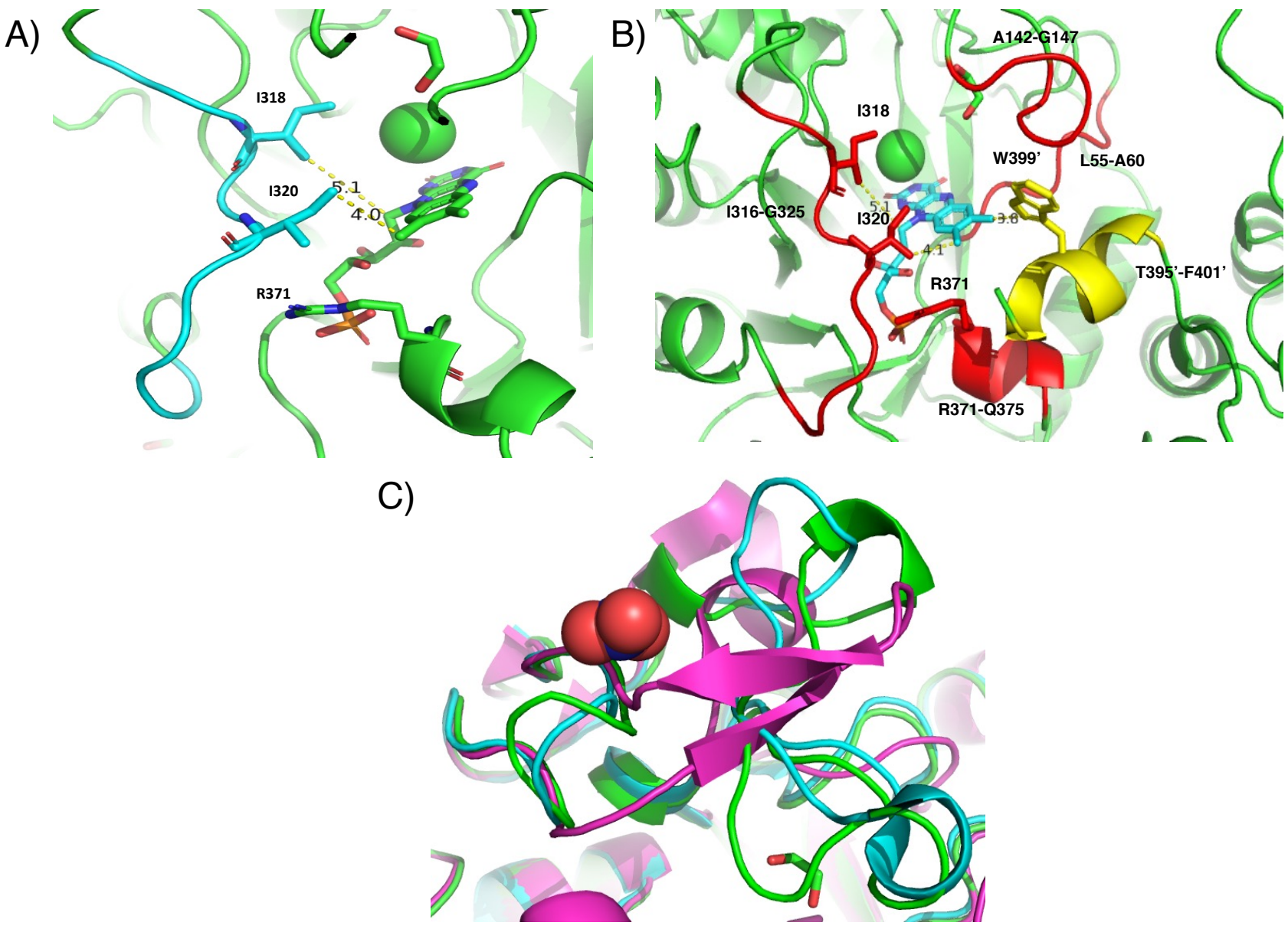

Figure S8. Details of the loop region from Ser 316 to Gly 325, the entrance of the catalytic pocket, and the superposition of cap regions from the crystal structures of *Af*OYE1. (A) The loop region from Ser 316 to Gly 325 (cyan) reaches the active site entrance of AfOYE1 (green) and is close to FMN (green). (B) C-terminal α helix (red), residues T395 to F401 (labeled as T395'-F401'), from adjacent protomer pointing to FMN together with three loops and one α-helices (red) (labeled as L55-A60, A142-G147, I316-G325, and R371-Q375) contribute to the narrow entrance of catalytic pocket. (C) Superposition of cap regions from the crystal structures of *Af*OYE1 (green), *Ca*OYE (cyan), and *GS*OYE (magenta).

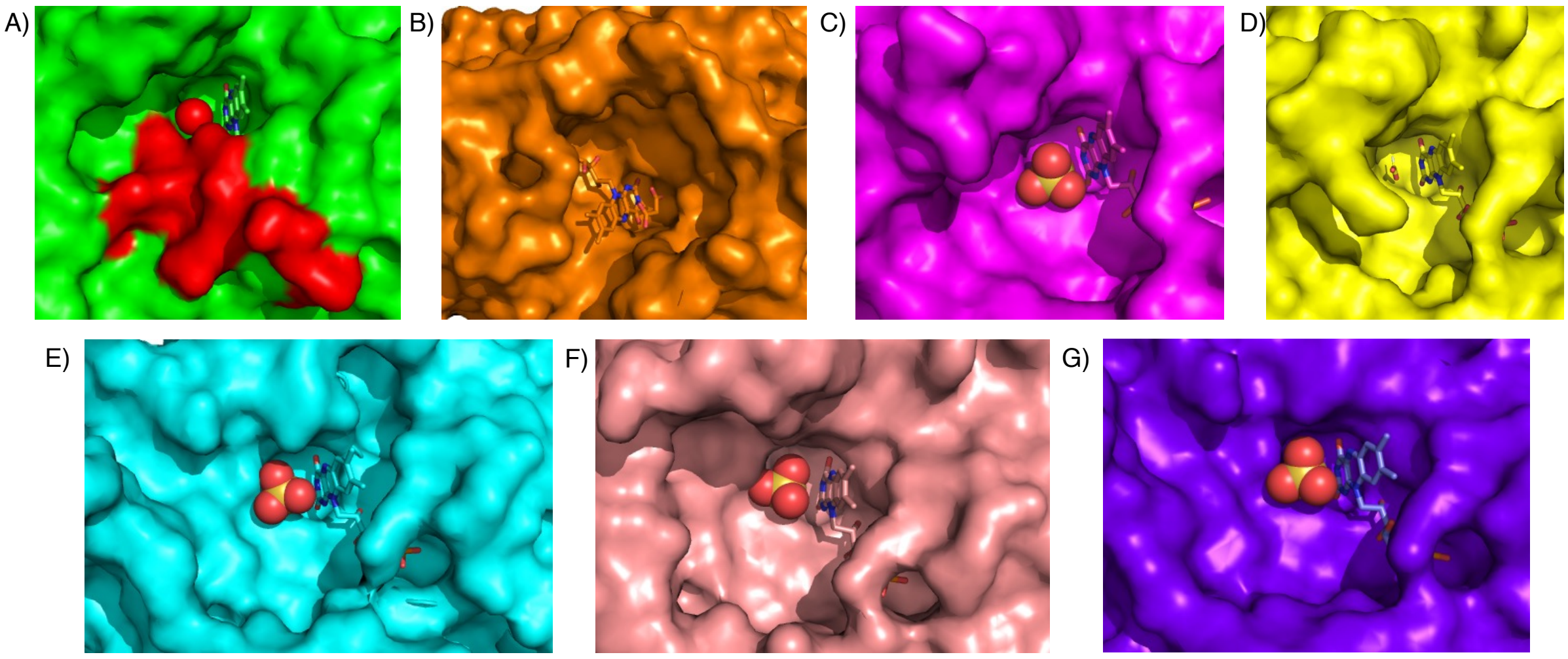

Figure S9. Surface representation of catalytic pocket among members of thermophilic-like Class III OYEs: (A) *Af*OYE1, (B) *Ca*OYE, (C) *An*OYE8, (D) *Bf*OYE4, YqjM (cyans), (F) *Ts*OYE, and (G) *Gk*OYE. Residues 316 to 325 in *Af*OYE1 close to the entrance of the catalytic pocket show red.

Table S1. Data collection and refinement statistics for *Af* OYE1.

**Data collection**	
Wavelength (Å)	0.9793
Resolution range (Å)	42.86 - 1.55 (1.605 - 1.55)
Space group	P 1 21 1
Unite cell	
a, b, c (Å)	47.47, 171.42, 50.48
α, β, γ (°)	90, 117.60, 90
Total reflections	667572 (53857)
Unique reflections	102393 (9786)
Multiplicity	6.5 (5.5)
Completeness (%)	99.28 (95.09)
I/*σ* (*I*)	25.71 (7.66)
R-merge (%)	0.05 (0.17)
R-meas (%)	0.05 (0.19)
R-pim (%)	0.02 (0.080)
CC1/2	0.99 (0.98)
**Refinment**	
Reflections used in refinement	102356 (9786)
Reflections/#R-free	4940 (467)
R-work/R-free (%)	0.1443 (0.1622) / 0.1676 (0.1954)
# of non-hydrogen	6997
Protein	5965
Ligands	112
Solvent	920
Protein residues	774
r. m. s. deviation	
Bond lengths (Å)	0.007
Bond angle (°)	0.95
Ramachandran plot	
Favored	96.88
Allowed	3.12
Outliers	0.00
Average B factor	11.12
Protein	9.78
Ligands	12.37
Solvent	19.66

Table S2. List of OYEs used for phylogenetic analysis and their accession numbers.

Enzyme	Class	Accession ID	Source
AcaryoER1	I	ABW29811	*Acaryochloris marina* MBIC11017
AcaryoER3	I	ABW32756	*Acaryochloris marina* MBIC11017
Achr-OYE4	I	AFK73188.1	*Achromobacter* sp. JA81
Chr-OYE2	I	ALE60337	*Chryseobacterium* sp. CA49
CtOYE	I	RUT11138.1	*Chroococcidiopsis thermalis*
CyanothER1	I	ACK64210	*Rippkaea orientalis* PCC 8801
CyanothER2	I	ACK65723	*Rippkaea orientalis* PCC 8801
GluER	I	AAW60280	*Gluconobacter oxydans* 621H
*Gs*OYE	I	XP_005703492	*Galdieria sulphuraria*
MR	I	AAC43569.1	*Pseudomonas putida*
NCR	I	AAV90509	*Zymomonas mobilis*
NemA	I	BAA13186	*Escherichia coli*
NerA	I	CAA74280	*Agrobacterium tumefaciens*
NospuncER1	I	ACC84535	*Nostoc punctiforme* PCC 73102
NostocER1	I	BAB73564	*Nostoc* sp. PCC 7120
LyngbyaER1	I	EAW37813	*Lyngbya* sp. PCC 8106
PETNR	I	AAB38683.1	*Enterobacter cloacae*
SYE1	I	AAN55488	*Shewanella oneidensis* MR-1
SYE3	I	AAN57126	*Shewanella oneidensis* MR-1
SynER	I	ABB56505	*Synechococcus elongatus* PCC 7942
XenB	I	AAF02539.1	*Pseudomonas fluorescens*
XenB2	I	AGS77941.1	*Pseudomonas putida*
YersER	I	WP_032896199	*Yersinia bercovieri*
*Ar*OYE1	II	AHL17019	*Ascochyta rabiei*
*Ar*OYE2	II	AHL17020	*Ascochyta rabiei*
*At*OPR1	II	NP_177794.1	*Arabidopsis thaliana*
AtOPR3	II	NP_001077884.1	*Arabidopsis thaliana*
ClER	II	EEQ40235	*Clavispora lusitaniae* ATCC 42720
*Cr*OYE2	II	XP_001699408.1	*Chlamydomonas reinhardtii*
CYE	II	BAD24850	*Kluyveromyces marxianus*
HYE1	II	KAG7904883.1	*Ogataea polymorpha*
HYE2	II	AAN09953	*Ogataea polymorpha*
KYE1	II	AAA98815.1	*Kluyveromyces lactis*

*Le*OPR1	II	NP_001234781.1	*Solanum lycopersicum*
*Le*OPR2	II	NP_001233868	*Solanum lycopersicum*
LeOPR3	II	NP_001233873.1	*Solanum lycopersicum*
EBP1	II	AAA18013	*Candida albicans*
*Mg*ER	II	EDK41665	*Meyerozyma guilliermondii* ATCC 6260
OYE1	II	Q02899	*Saccharomyces pastorianus*
OYE2	II	Q03558	*Saccharomyces cerevisiae* S288C
OYE2.6	II	ABN66026	*Scheffersomyces stipitis* CBS 6054
OYE3	II	P41816	*Saccharomyces cerevisiae* S288C
Achr-OYE3	III	AFK73187.1	*Achromobacter* sp. JA81
*Af*OYE1	III	XP_041144250.1	*Aspergillus flavus* NRRL3357
AnabaenaER3	III	ABA25236.1	*Trichormus variabilis* ATCC 29413
*An*OYE8	III	XP_001399273.1	*Aspergillus niger*
*Bf*OYE	III	XP_001554780.1	*Botrytis cinerea* B05.10
*Ca*OYE	III	WP_015941499.1	*Chloroflexus aggregans*
Chr-OYE3	III	AFK73187.1	*Achromobacter* sp. JA81
*Cr*OYE1	III	XP_042928859.1	*Chlamydomonas reinhardtii*
*Dr*ER	III	AAF11740	*Deinococcus radiodurans* R1
FOYE-1	III	KRH78075	*Ferrovum* sp. JA12
*Geo*ER	III	BAO37313	*Geobacillus* sp. #30
*Gk*OYE	III	BAD76617	*Geobacillus kaustophilus* HTA426
GloeoER	III	BAC91769	*Gloeobacter violaceus* PCC 7421
OYERo2	III	ALL54975	*Rhodococcus opacus* 1CP
PfvC	III	AFF18622.1	*Arthrobacter* sp. JBH1
RhrER	III	AMD82542.1	*Rhodococcus rhodochrous* ATCC 17895
*Rm*ER	III	ABF11721	*Cupriavidus metallidurans* CH34
TOYE	III	RUT11138.1	*Thermoanaerobacter pseudethanolicus* ATCC
*Ts*OYE	III	CAP16804	*Thermus scotoductus* SA-01
XenA	III	AAF02538	*Pseudomonas putida*
YqjM	III	BAA12619	*Bacillus subtilis*
Chr-OYE1	IV	ALE60336.1	*Chryseobacterium* sp. CA49
Ppo-ER1	IV	AHC19521	*Paenibacillus polymyxa* CR1
Ppo-ER2	IV	AIW41616	*Paenibacillus polymyxa* CR1
Ppo-ER3	IV	QBR53093	*Paenibacillus polymyxa*
Rer-ER7	IV	QBR53095.1	*Rhodococcus erythropolis*
*Ang*OYE11	V	XP_001390672.2	*Aspergillus niger* CBS 513.88

*Ang*OYE12	V	XP_001395504.2	*Aspergillus niger* CBS 513.88
*Ar*OYE3	V	AHL17021	*Ascochyta rabiei*
*Ar*OYE6	V	AHL17024	*Ascochyta rabiei*
*Bf*OYE6	V	XP_001547575.1	*Botrytis cinerea* B05.10
FbOYE	V	TAL24079.1	*Frankiales bacterium*
MoOYE4	V	XP_003720070.1	*Pyricularia oryzae* 70-15
Nox	V	WP_029256809.1	*Rhodococcus erythropolis*
PcOYE8	V	XP_002560444.1	*Penicillium rubens Wisconsin* 54-1255
BcOYE	VI	WP_098362597.1	*Bacillus cereus*
LacER	VI	ADK19581	*Lacticaseibacillus paracasei*
Lla-ER	VI	WP_046781786.1	*Lactococcus cremoris*
*Mf*OYE	VI	WP_039343246.1	*Burkholderia*
POYE	VI	WP_119715197.1	*Propionibacterium ruminifibrarum*
YqiG	VI	QBR53092	*Bacillus subtilis*

Table S3. Polymerase chain reaction primers for mutagenesis.

	Oligonucleotides
H211L-F	5'-TTC GAT GCC ATC GAG ATC CTG GCG GCG CAT GGT TAT CTG-3'
H211L-R	5'-CAG ATA ACC ATG CGC CGC CAG GAT CTC GAT GGC ATC GAA-3'
H214L-F	5'-ATC GAG ATC CAT GCG GCG CTG GGT TAT CTG CTG CAC CAG-3'
H214L-R	5'-CTG GTG CAG CAG ATA ACC CAG CGC CGC ATG GAT CTC GAT-3'
Y216F-F	5'-ATC CAT GCG GCG CAT GGT TTT CTG CTG CAC CAG TTT CTG-3'
Y216F-R	5'-CAG AAA CTG GTG CAG CAG AAA ACC ATG CGC CGC ATG GAT-3'

Table S4. Comparison of *Af*OYE1 characteristics with those of other thermophilic-like Class III OYEs. In the crystal, oligomerization data were checked from crystallographic structures; Interface area, numbers of interacting residues, and interactions involving the dimerization surface were calculated by PISA software; Catalytic pocket volume, surface, and depth were calculated by Proteins. Plus/DoGSiteScorer software. Hb: Hydrogen bonds. Sb: Salt bridges.

Protein (PDB ID)	In crystal	Interface (Å^2)	Interacting residues	Interactions (Hb, Sb)	Pocket Volume (Å^3)	Pocket Surface (Å^2)	Pocket Depth (Å)
*An*OYE8 (7QFX)	tetramer	2796	71	(18, 4)	703.38	864.99	16.97
*Ca*OYE (7O0T)	tetramer	1451	34	(16, 8)	758.08	748.47	22.74
*Bf*OYE4 (7BLF)	dimer	2121	53	(12, 3)	829.18	838.56	26.51
YqjM (1Z41)	dimer	660	31	(4, 3)	818.95	849.48	23.74
*Ts*ER (3HF3)	tetramer	1262	33	(17, 5)	653.90	690.12	20.21
*Gk*OYE (3GR7)	dimer	1199	30	(16, 0)	877.28	818.83	19.34
*Af*OYE1 (8J59)	dimer	492	41	(7, 6)	1203.07	1126.53	24.23

14. The data of the products from the *Af*OYE1-catalyzed bioreduction

Cyclopentanone (**1b**)

Colorless oil; ^{1}HNMR ($CDCl_3$): δ = 2.13-2.17 (m, 4H), 1.93-1.97 (m, 4H) ppm.

(*S*)-2-methylcyclopentanone (**2b**)

Colorless oil; $[\alpha]^{25}_{D}$ = +16.5º (c 0.4, $CHCl_3$), the optical rotation indicates its configuration to be (*S*) (lit. (Shimoda et al. 2004), $[\alpha]^{25}_{D}$ = +114.9º (c 0.52, $CHCl_3$); retention time: t_R(*S*) 12.31 min (Chiral GC with CHIRASIL-DEX CB column); ^{1}HNMR ($CDCl_3$): δ = 2.19-2.33 (m, 2H), 2.05-2.15 (m, 2H), 1.95-2.03 (m, 1H), 1.74-1.82 (m, 1H), 1.42-1.53 (m, 1H), 1.08 (d, J = 6.96 Hz, 3H) ppm.

Cyclohexanone (**3b**)

Colorless oil; ^{1}HNMR ($CDCl_3$): δ = 2.31-2.34 (m, 4H), 1.82-1.88 (m, 4H), 1.69-1.74 (m, 2H) ppm.

(*S*)-2-methylcyclohexanone (**4b**)

Colorless oil; retention time: t_R(*S*) 7.26 min (Chiral GC with CHIRASIL-DEX CB column); ^{1}HNMR ($CDCl_3$): δ = 2.29-2.35 (m, 2H), 2.18-2.26 (m, 1H), 1.98-2.04 (m, 2H), 1.76-1.80 (m, 1H), 1.55-1.64 (m, 2H), 1.29-1.35 (m, 1H), 0.96 (d, J = 6.60 Hz, 3H) ppm.

Propionamide (**5b**)

Colorless oil; ^{1}HNMR ($CDCl_3$): δ = 2.26 (q, J = 7.86 Hz, 2H), 0.96 (t, J = 7.86 Hz, 3H) ppm.

Phenylnitroethane (**6b**)

Colorless oil; ^{1}HNMR ($CDCl_3$): δ = 7.30-7.37 (m, 3H), 7.22-7.24 (m, 2H), 4.64 (t, J = 7.34 Hz, 2H), 3.35 (t, J = 7.34 Hz, 2H) ppm.

1-Phenyl-2-nitropropane (**7b**)

Colorless oil; ^{1}HNMR ($CDCl_3$): δ = 7.29-7.36 (m, 3H), 7.18-7.20 (m, 2H), 4.78-4.83 (m, 1H), 3.36 (dd, J = 7.52 Hz, J = 14.00 Hz, 1H), 3.04 (dd, J = 6.80 Hz, J = 14.00 Hz, 1H), 1.57 (d, J = 6.66 Hz, 3H) ppm.

Citronellal (**8b**)

Colorless oil; ^{1}HNMR ($CDCl_3$): δ = 5.06-5.10 (m, 1H), 2.37-2.43 (m, 1H), 2.19-2.26 (m, 1H), 1.96-2.09 (m, 3H), 1.68 (s, 3H), 1.60 (s, 3H), 1.24-1.39 (m, 2H), 0.97 (d, J = 6.66 Hz, 3H) ppm.

15. Selected ^{1}H NMR spectra and GC chromatograms

Cyclopentanone (**1b**)

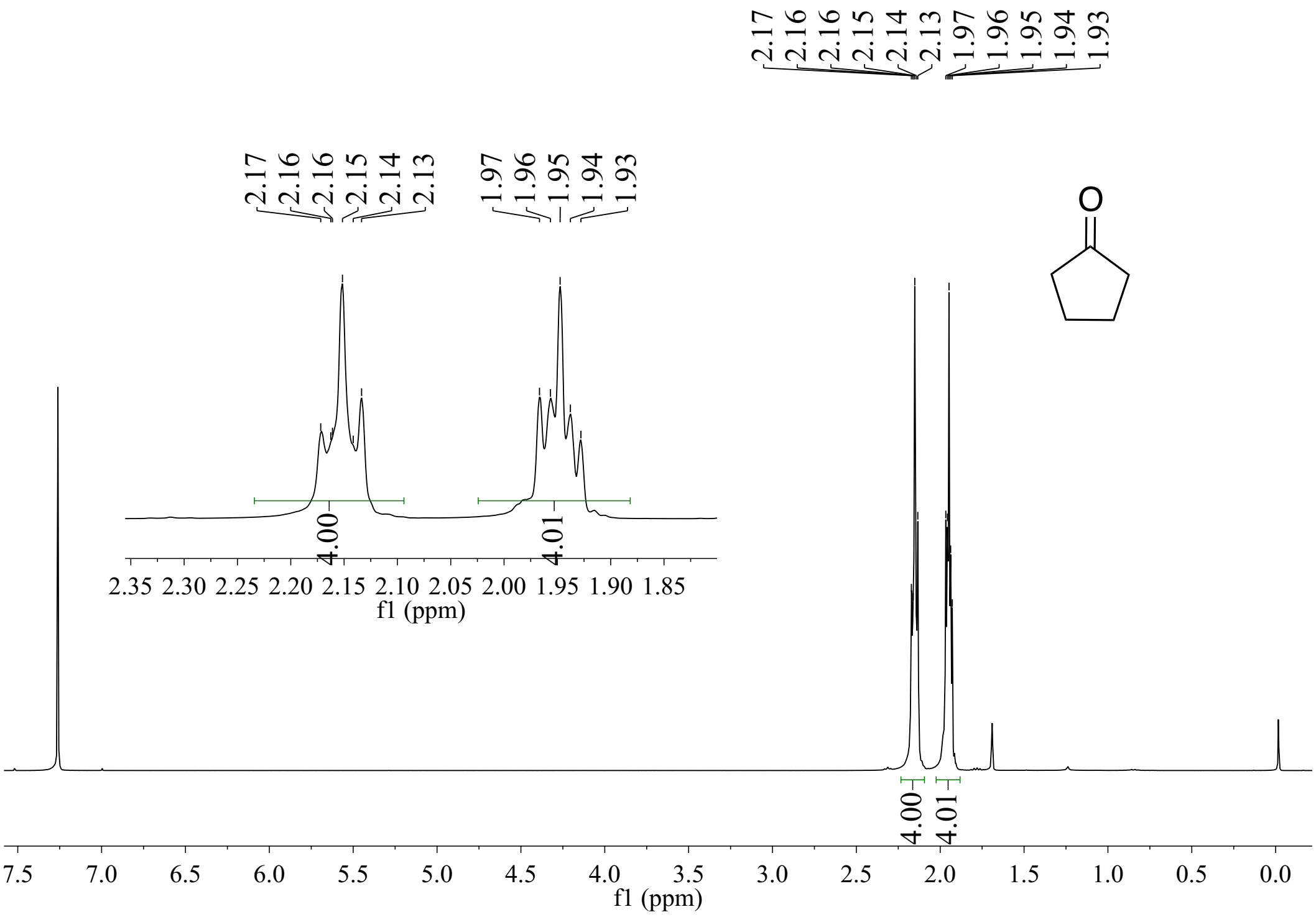

(*S*)-2-methylcyclopentanone (**2b**)

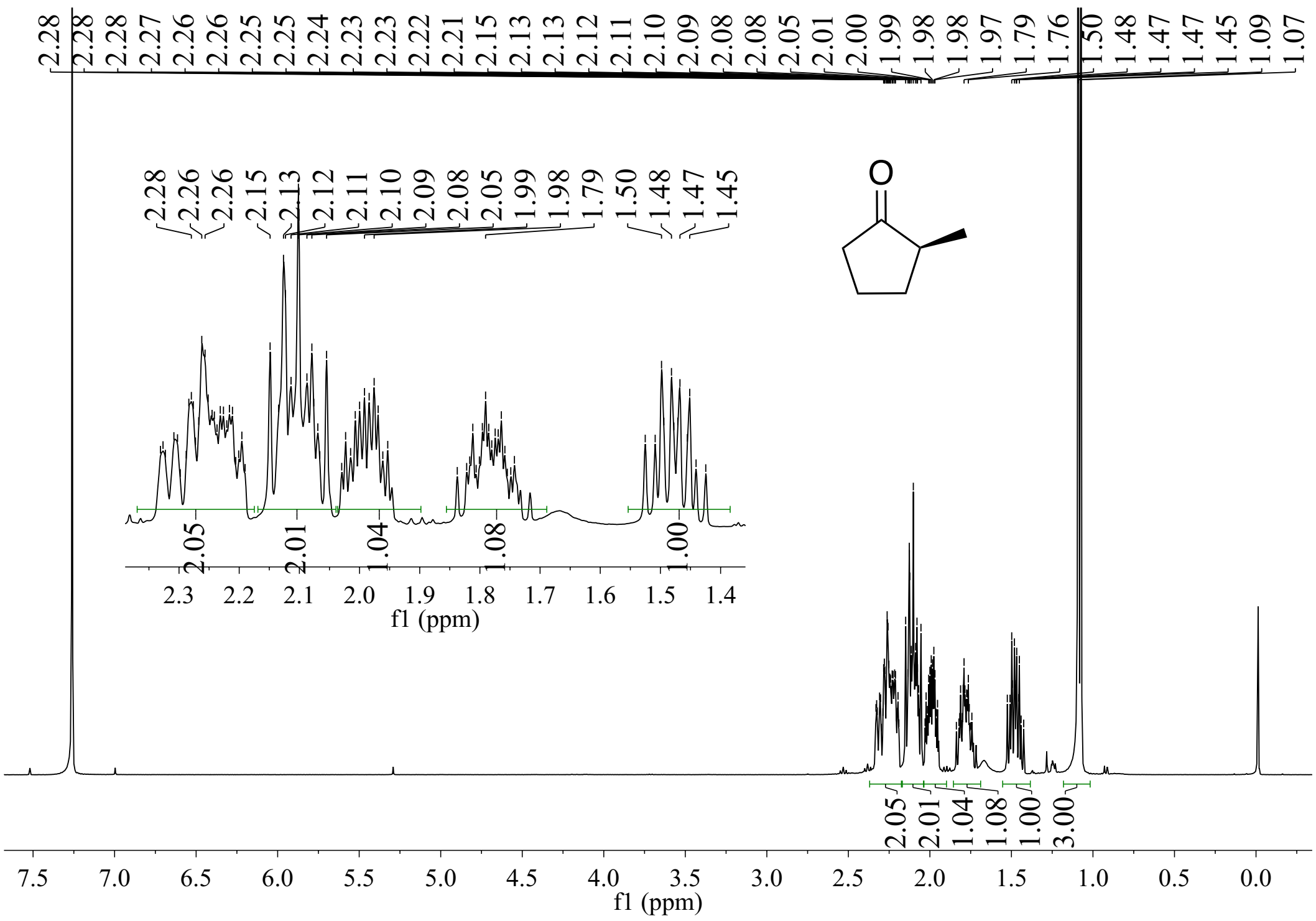

GC chromatogram of Racemic 2-methylcyclopentanone and (*S*)- 2-methylcyclopentanone (**2b**) from *Af*OYE1-catalyzed bioreduction

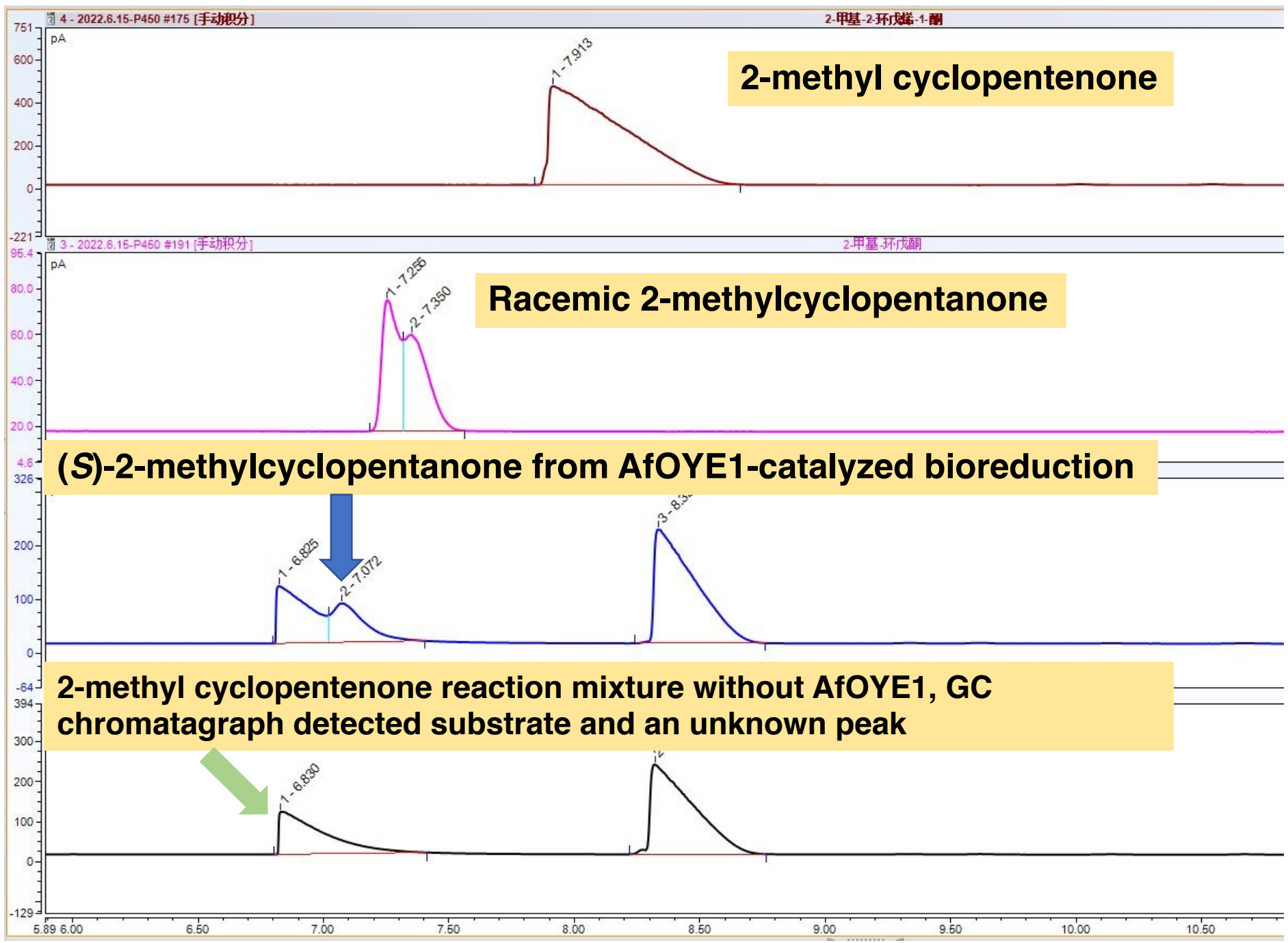

Cyclohexanone (**3b**)

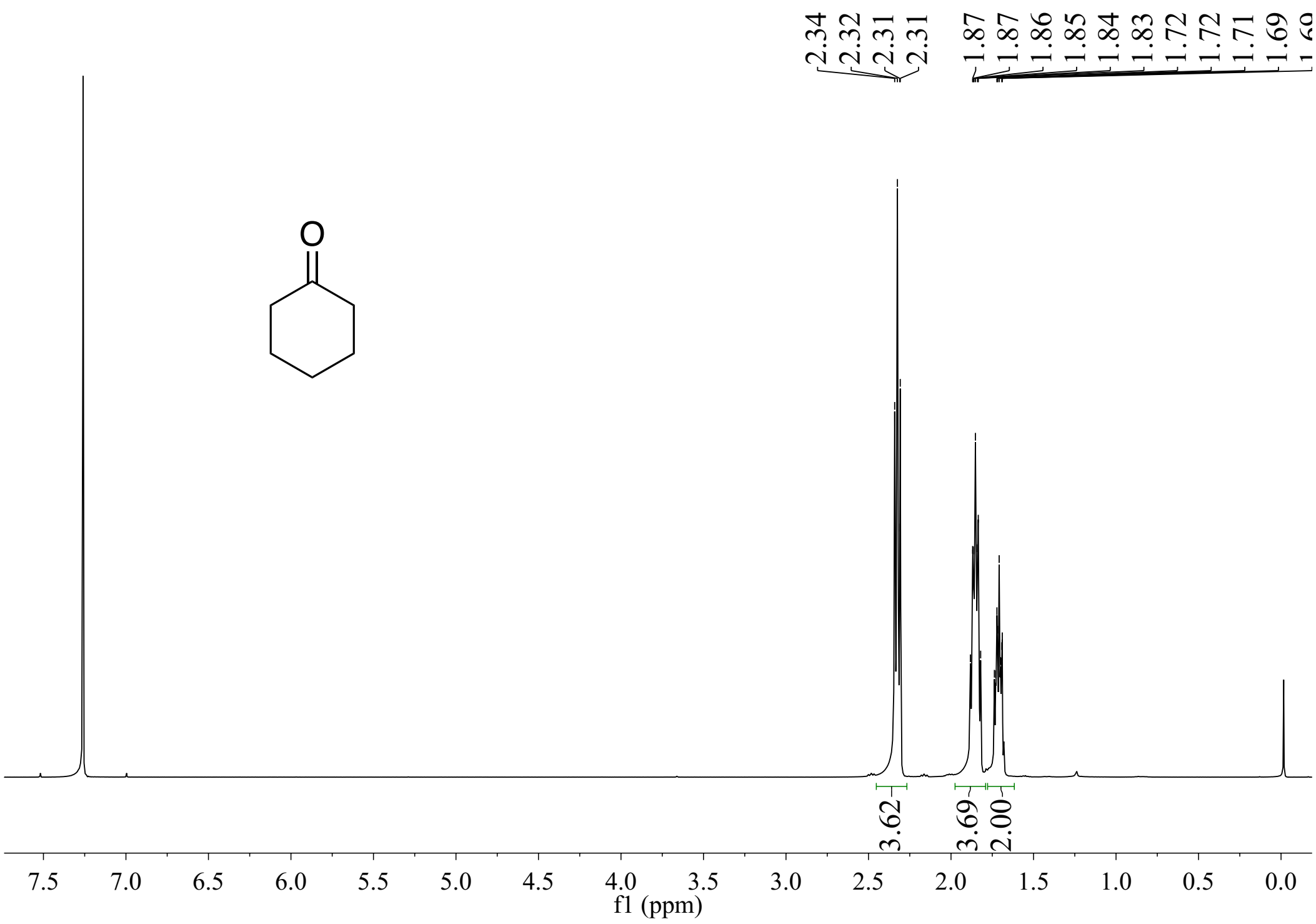

(*S*)-2-methylcyclohexanone (**4b**)

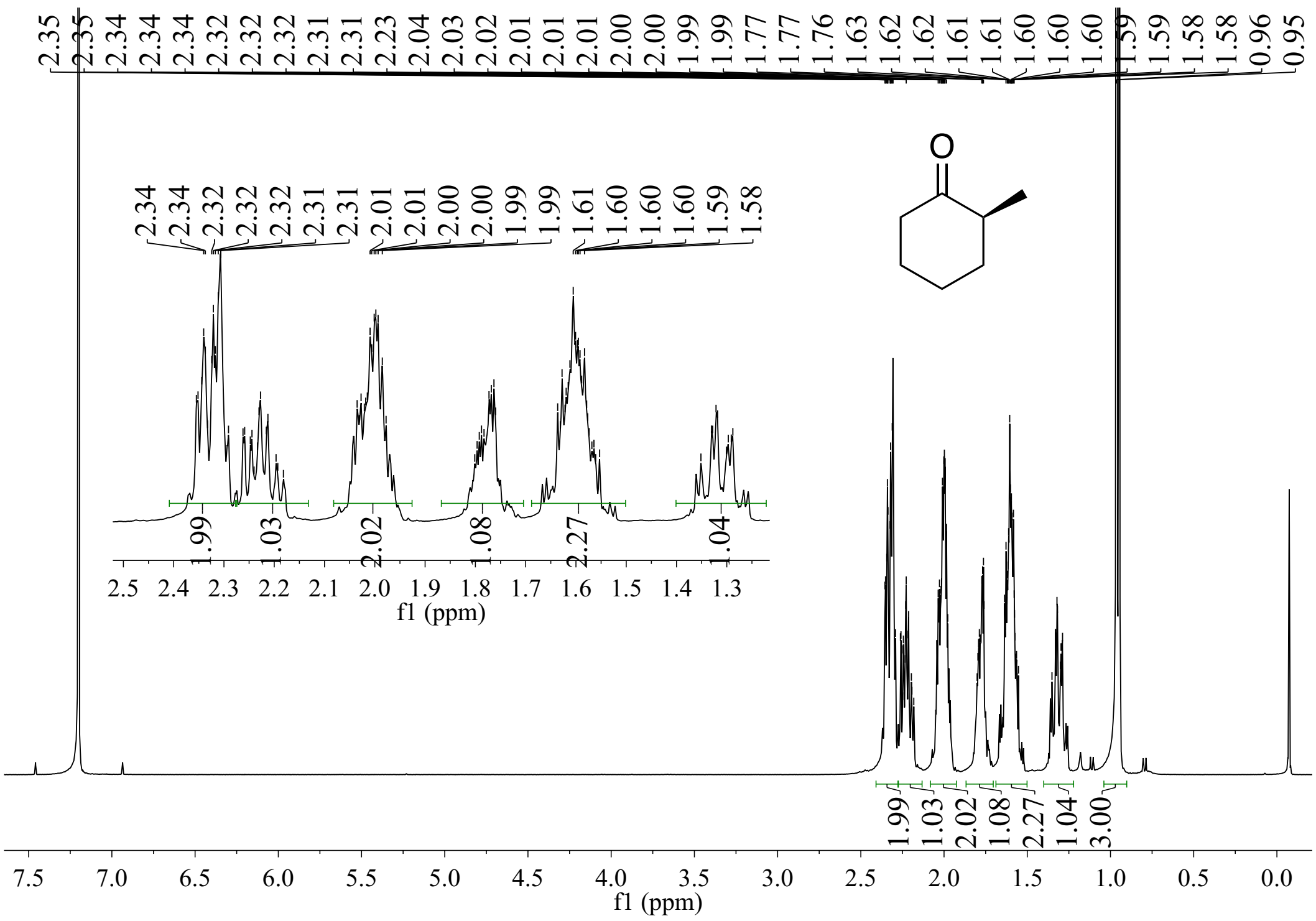

GC chromatogram of Racemic 2-methylcyclohexanone and (*S*)-2-methylcyclohexanone (**4b**) from *Af*OYE1-catalyzed bioreduction

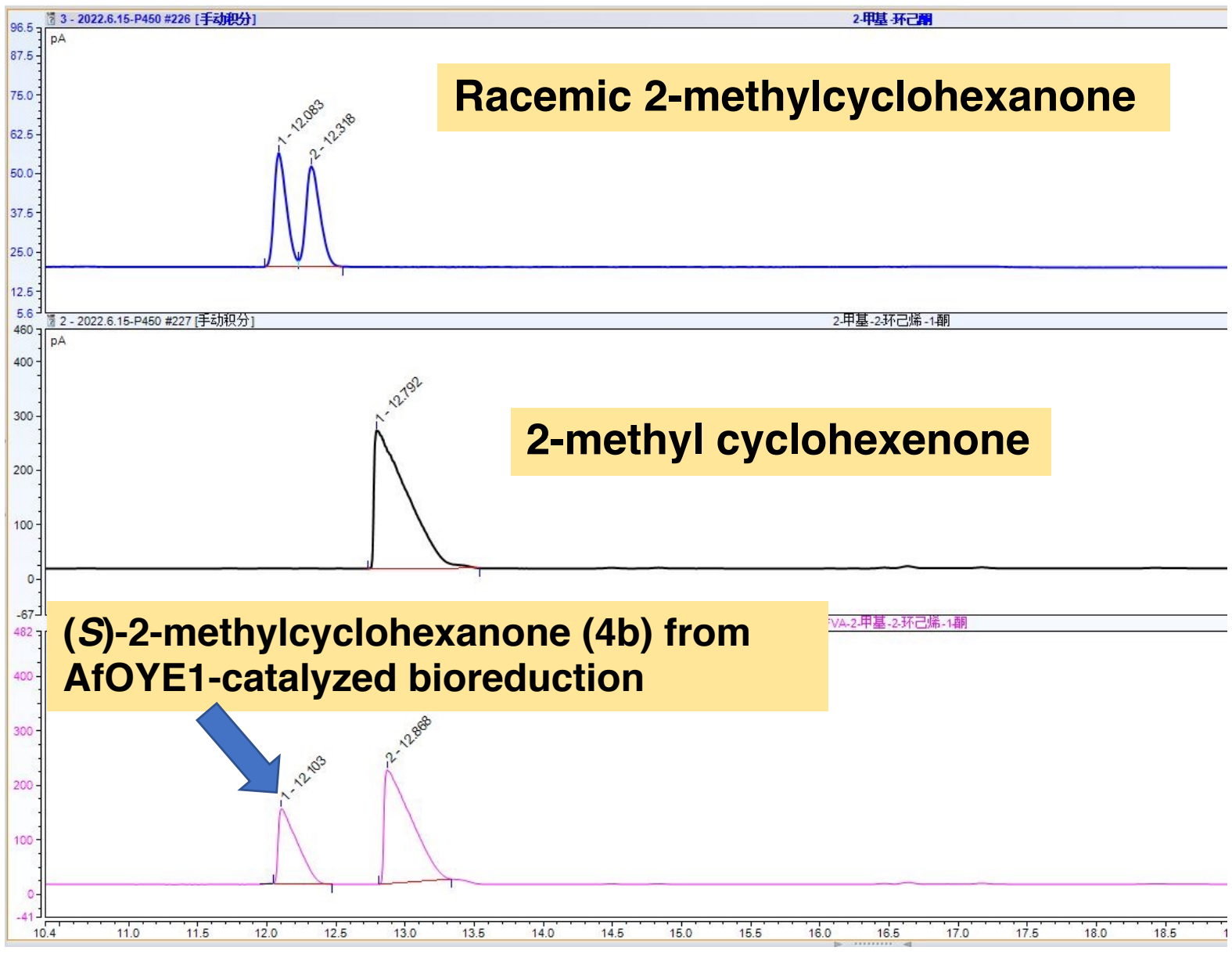

Propionamide (**5b**)

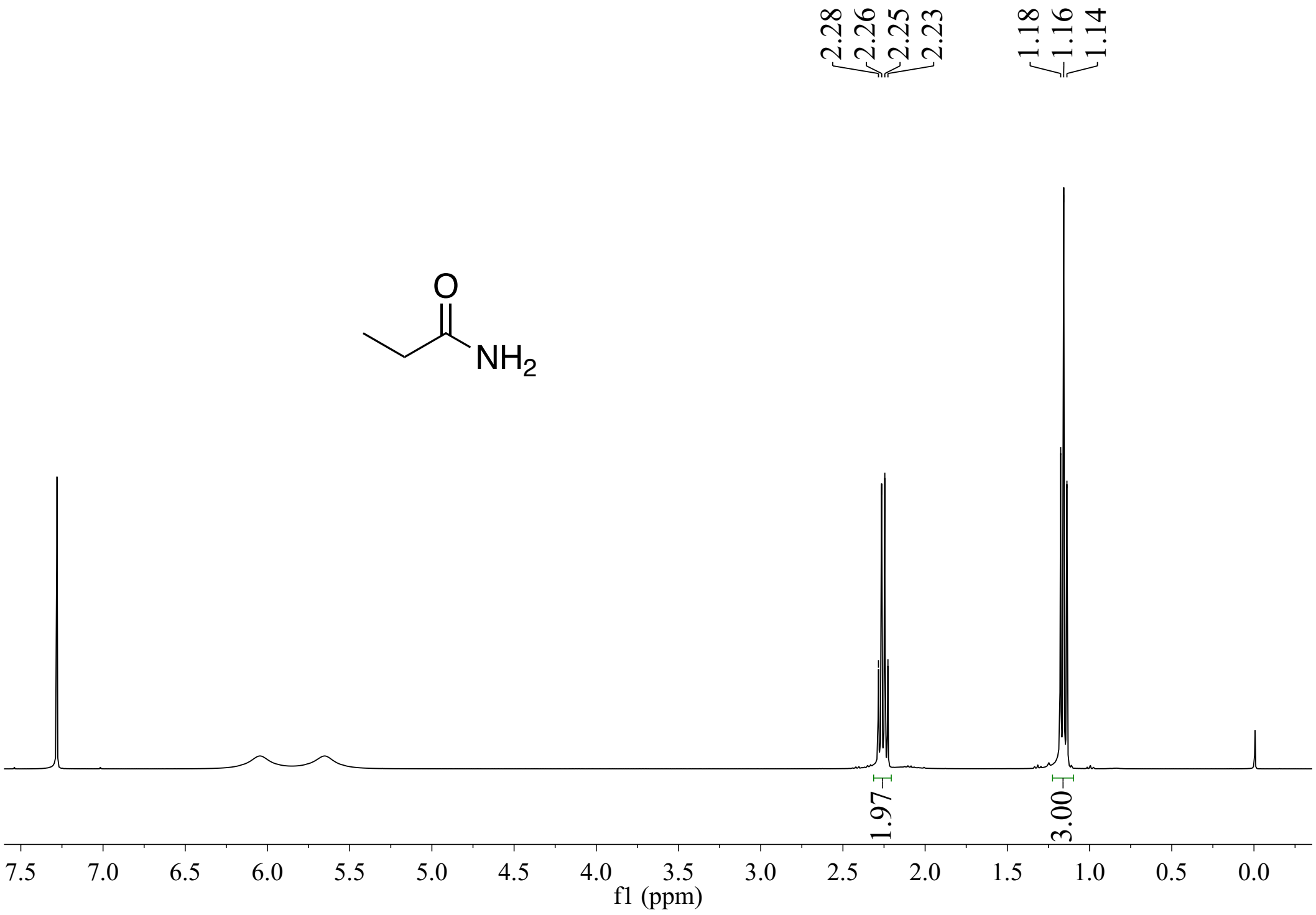

Phenylnitroethane (**6b**)

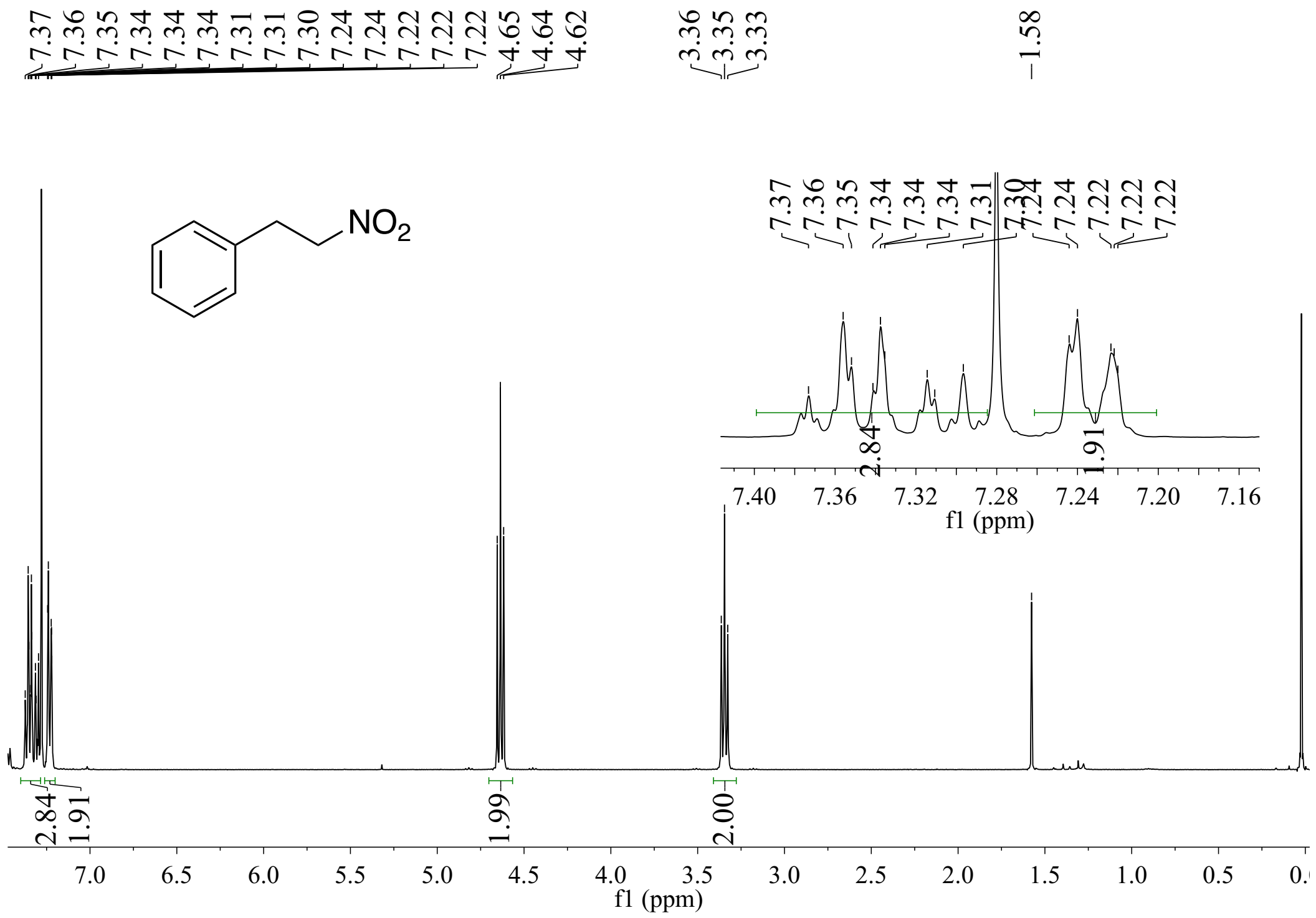

1-Phenyl-2-nitropropane (**7b**)

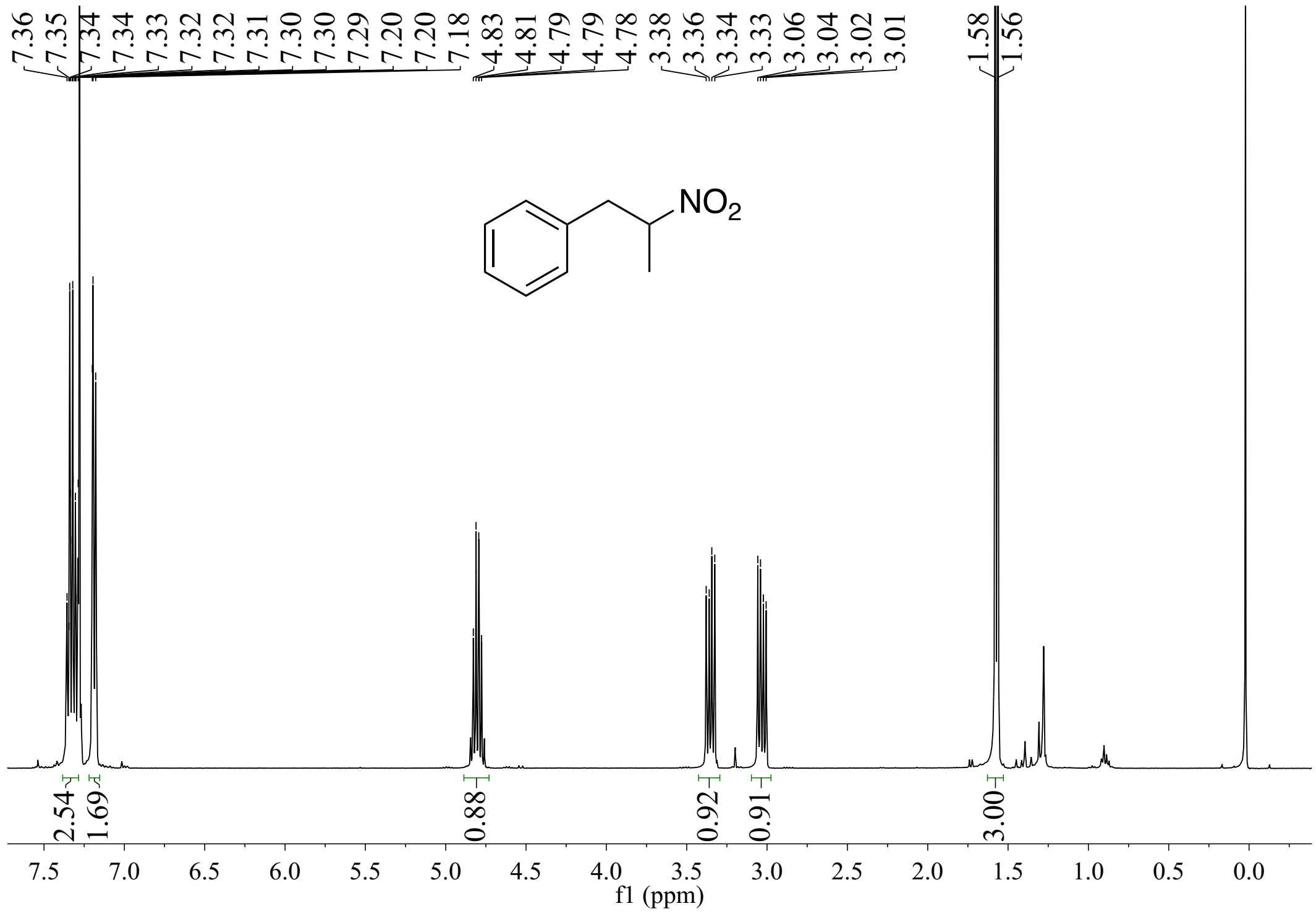

Citronellal (**8b**)

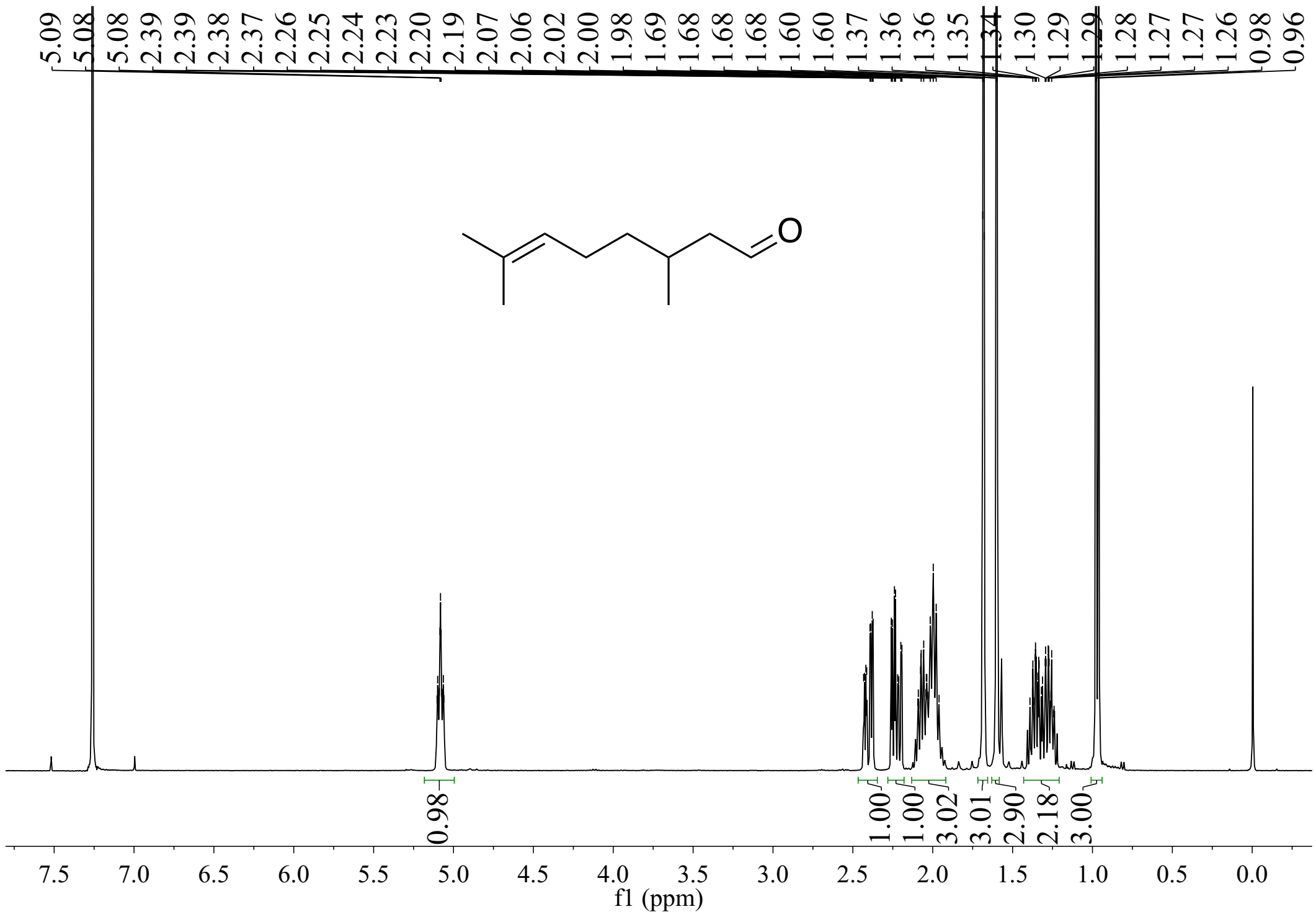

16. The sequence information of *Af*OYE1.

The amino acid sequence of afvA (UniProt: B8N8Q9, *Af*OYE1) with N-terminal His6-tag followed by a tobacco etch virus (TEV) protease cleavage site.

MGSSHHHHHHENLYFQGISYYTPAQVPPAGTQVEGSTKLFSPLTIRGVTFPNRLFLAP
LCQYSAKDGYANDWHLTHIGGIVQRGPGLAIMEATAVQKVGRITPQDLGLYDDGHI
EPLKRITEFAHSQSQKIGIQLAHAGRKASAVAPWLSGNAMAVKEVGGWPDDIVAPS
AIPQEEGINAVPKVLTGEDIGVLKKDWAEAAKRAVRANFDAIEIHAAHGYLLHQFLS
PVSNRRTDKYGGSFENRVRILLEICEEVRAVIPTAMPLLVRISATDWFEFDDNLTKEFP
ESWTVAQSIRLALLLADRGVDLVDVSSGGIHAKSAIAIRSGPGYQVHFAQEIKKAVG
EKLLISAVGGIKTGALAEEVVQSGIDAVQAGRWFQQNPGLVRAFANELGVKVRMAT
QIDWSFEGRGKKAKKSSL

The codon-optimized nucleotide sequence of *afvA*.

ATGGGCAGTAGTCATCACCACCATCACCACGAGAATCTGTACTTCCAAGGCATCA
GTTACTATACCCCAGCGCAAGTTCCGCCGGCCGGTACGCAAGTTGAAGGTAGCA
CCAAGCTGTTCAGCCCGCTGACGATTCGCGGCGTGACGTTTCCGAATCGTCTGTT
TCTGGCCCCACTGTGCCAGTACAGTGCGAAAGATGGCTATGCGAACGACTGGCA
TCTGACCCACATTGGTGGCATTGTTCAGCGTGGCCCGGGTCTGGCCATCATGGAA
GCGACCGCCGTTCAAAAAGTTGGCCGTATCACGCCGCAAGATCTCGGTCTGTATG
ATGACGGCCACATCGAGCCACTGAAGCGCATCACCGAGTTCGCCCATAGTCAGA
GCCAGAAGATCGGTATCCAGCTGGCGCACGCGGGTCGTAAAGCCAGTGCCGTTG
CGCCGTGGCTGAGTGGTAATGCGATGGCCGTGAAAGAGGTTGGTGGCTGGCCGG
ATGACATCGTGGCGCCAAGCGCCATCCCGCAAGAAGAGGGCATCAATGCCGTGC
CAAAAGTGCTGACGGGTGAGGACATCGGCGTGCTGAAAAAAGACTGGGCGGAA
GCGGCGAAACGTGCGGTTCGTGCCAACTTCGATGCCATCGAGATCCATGCGGCG
CATGGTTATCTGCTGCACCAGTTTCTGAGTCCGGTTAGCAACCGTCGCACGGACA
AATACGGCGGCAGCTTTGAAAACCGTGTGCGCATTCTGCTGGAGATCTGCGAAG
AAGTTCGCGCGGTGATTCCAACCGCCATGCCGCTCCTCGTTCGCATCAGCGCCAC
GGACTGGTTCGAGTTCGACGACAATCTGACGAAGGAATTCCCGGAGAGCTGGAC
CGTGGCGCAGAGTATCCGTCTGGCGCTGCTGCTGGCGGATCGTGGCGTTGATCTG
GTGGACGTGAGCAGCGGTGGCATTCATGCGAAAAGTGCCATCGCCATCCGTAGT
GGCCCGGGCTACCAAGTTCACTTCGCGCAAGAAATCAAAAAGGCGGTGGGTGAA
AAGCTGCTGATCAGTGCCGTTGGCGGCATTAAAACCGGTGCGCTGGCCGAAGAA
GTTGTTCAGAGTGGCATTGACGCCGTTCAAGCCGGCCGTTGGTTTCAGCAGAACC
CGGGTCTGGTTCGCGCGTTCGCCAACGAGCTGGGTGTGAAGGTTCGTATGGCCAC
GCAGATCGACTGGAGCTTCGAATAA

Reference:

Shimoda K, Kubota N, Hamada H, Kaji M, Hirata T (2004) Asymmetric reduction of enones with *Synechococcus* sp. PCC 7942. Tetrahedron: Asymmetry 15:1677-1679
