# Supplementary material for: Structural and functional characterization of a new thermophilic-like OYE from Aspergillus flavus
Source: Appl Microbiol Biotechnol. 2024 Jan 13;108(1):134. doi: 10.1007/s00253-023-12963-w (PMC10787880; doi:10.1007/s00253-023-12963-w)
Supplement: Supplementary file 1 — Supplementary file1 (PDF 22613 KB) [file 253_2023_12963_MOESM1_ESM.pdf]

Structural and Functional Characterization of a New Thermophilic-like OYE from *Aspergillus flavus*

Na Li[1#], Yuan Wang[1#], Yinyin Meng[2], Yangyong Lv[1], Shuaibing Zhang[1], Shan Wei[1], Pingan Ma, Yuansen Hu[1*], Hui Lin[2*]

[1.] College of Biological Engineering, Henan University of Technology, 100 Lianhua Street, Zhengzhou, 450001, Henan, China.

[2.] Henan International Joint Laboratory of Biocatalysis and Bio-based Products, College of Life Sciences, Henan Agricultural University, 95 Wenhua Road, Zhengzhou 450002, China.

[#] These authors contributed equally to the work.

Corresponding authors: Yuansen Hu (Email: hys308@126.com); Hui Lin (Email: huilin@henau.edu.cn)

## Contents

1. **Figure S1.** Sequence alignment performed by Clustal Omega of the *Af*OYE1 from *Aspergillus flavus* with representative OYEs from different classes
2. **Figure S2**. SDS-PAGE analysis of the purified *Af*OYE1.
3. **Figure S3**. Steady-state kinetics of *Af*OYE1-catalyzed reduction of 2-cyclopentenone.
4. **Figure S4.** Surface charge distribution of *Af*OYE1.
5. **Figure S5.** The plot of FMN cofactor interactions in the actives site of *Af*OYE1.
6. **Figure S6.** Superposition of monomers from the crystal structures of *Af*OYE1, *Bf*OYE4, and *An*OYE8.
7. **Figure S7.** A view showing N-terminal loops and protruding α6-helix of *Af*OYE1 promote dimerization in five copes of crystallographically independent units.
8. **Figure S8.** Details of the loop region from Ser 316 to Gly 325, the entrance of the catalytic pocket, and the superposition of cap regions from the crystal structures of *Af*OYE1.
9. **Figure S9.** Surface representation of catalytic pocket among members of thermophilic-like Class III OYEs.
10. **Table S1**. Data collection and refinement statistics for *Af*OYE1.
11. **Table S2.** List of OYEs used for phylogenetic analysis and their accession numbers.
12. **Table S3**. Polymerase chain reaction primers for mutagenesis.
13. **Table S4**. Comparison of *Af*OYE1 characteristics with those of other thermophilic-like Class III OYEs.
14. The data of the products from the *Af*OYE1-catalyzed reduction.
15. Selected $^1$H NMR spectra and GC chromatograms.
16. The sequence information of *Af*OYE1.

```
Nox             --------------------------MTTAPTPSIFEPARLG-PLTLRNRIVKAATFE--      31
YqiG            ---------------------------MNPKYKPLFEPFTFKSGVTIDNRIAVAPMTHYA      33
Chr-OYE1        ------------------------------MSTESLFTPFKYK-NLELKNRIVMAPMTRAQ      30
AfOYE1          MTQDIIDNIAAEGISYYTPAQVPPAGTQVEGSTKLFSPLTIR-GVTFPNRLFLAPLCQYS      59
YqjM            ------------------------------MARKLFTPITIK-DMTLKNRIVMSPMCMYS      29
PETNR           -----------------------------MSAEKLFTPLKVG-AVTAPNRVFMAPLTRLR      30
OYE1            -----------------MSFVKDFKPQALGDTNLFKPIKIG-NNELLHRAVIPPLTRMR      41
                                                  :* *          :*

Nox             GVMPRGAVSDDL-INFHAEVARGGAAMTTVAYCAVSP--GGRVHRDTLVMDERALPGLRR      88
YqiG            SNEDGTISEAE--LDYIIPR-SKEMGMVITACANVTPD-GKAFPGQPAIHDDSNIPGLKK      89
Chr-OYE1        SD--NGVPTQQI-ADYYARRAAAEVGLILSEGTVINRPASKNMQNIPDFYGTEALNGWKN      87
AfOYE1          AK--DG-YANDWHLTHIGGIVQRGPGLAIMEATAVQKV-GRITPQDLGLYDDGHIEPLKR      115
YqjM            SHEKDG-KLTPFHMAHYISRAIGQVGLIIVEASAVNPQ-GRITDQDLGIWSDEHIEGFAK      87
PETNR           SIEPGDIPTPLM-GEYYRQRA--SAGLIISEATQISAQ-AKGYAGAPGLHSPEQIAAWKK      86
OYE1            ALHPGNIPNRDWAVEYYTQRAQRPGTMIITEGAFISPQ-AGGYDNAPGVWSEEQMVEWTK      100
                .              .          :       :    .        . .   :    .

Nox             LTDAVHAEGALAAAQIGHAGLVANTLSNK-----------------TKTLAPSTRL-SPP      130
YqiG            LAQAIQAQGAKAVVQIHHGGIECPSEL--VPQQ--------------DVVGPSDVF--DN      131
Chr-OYE1        VIDAVHHNGGKMGPQIWHVGDTRSTPD--YPL---------------------------E      118
AfOYE1          ITEFAHSQSQKIGIQLAHAGRKASAVAPWLSGNAMAVKEVG--GWPDDIVAPSAIPQEEG      173
YqjM            LTEQVKEQGSKIGIQLAHAGRKAELEGDIFA--------------------PSAIAFDEQ      127
PETNR           ITAGVHAEDGRIAVQLWHTGRISHSSI--QPGGQAPVSASALNANTRTSLRDENGNAIRV      144
OYE1            IFNAIHEKKSFVWVQLWVLGWAAFPDN--LARDGLRYDSA----SDNVFMDAEQEAKAKK      154
                :    : :      *:   *

Nox             AMGLVKGATLAELDGVVSDFERTARVAVDAGFDAIEVHLGHNYLLSSFMSPNLNKRHDRY      190
YqiG            GKQIARALKEEEVENIVKAFGEATRRAIEAGFDGVEIHGANGYLIQQFYSPKTNQRTDRW      191
Chr-OYE1        DMEKASTMTLEDIQDTIAQFAASAKSAKDLGFDVLEIHGAHGYLIDQFFWEGTNTRTDEY      178
AfOYE1          INAVPKVLTGEDIGVLKKDWAEAAKRAVRANFDAIEIHAAHGYLLHQFLSPVSNRRTDKY      233
YqjM            -SATPVEMSAEKVKETVQEFKQAAARAKEAGFDVIEIHAAHGYLIHEFLSPLSNHRTDEY      186
PETNR           DTTTPRALELDEIPGIVNDFRQAVANAREAGFDLVELHSAHGYLLHQFLSPSSNQRTDQY      204
OYE1            ANNPQHSLTKDEIKQYIKEYVQAAKNSIAAGADGVEIHSANGYLLNQFLDPHSNTRTDEY      214
                           .:      :  :.  :   . * :*:* .:.**: .*     * * *.:

Nox             GGS-VAKRAEYPRRVIEAVRVAAGSSV----AV------TAKFNMSDGVPKGLWLDQSLP      239
YqiG            GGS-DEKRLAFPLAIVDEVKKAASEHAKGAFLVGYRLSPEEPETPGLTMTET--------      242
Chr-OYE1        GGKTIKERSRFAVDVVKAIRAAVGED----FTIIIRLSQWKQQDYSVKLAHTP--EE---      229
AfOYE1          GGS-FENRVRILLEICEEVRAVIPTA----MPLLVRISATDWFEFDDNLTKEF--PESWT      286
YqjM            GGS-PENRYRFLREIIDEVKQVWD------GPLFVRVSASDYTDKG------------LD      227
PETNR           GGS-VENRARLVLEVVDAVCNEWSAD-----RIGIRVSPIGTFQNVDNGPNE------EA      252
OYE1            GGS-IENRARFTLEVVDALVEAIGHE-----KVGLRLSPYGVFNSMSGGAET------GI      262
                **.   :*      : . :             :

Nox             IAQILEADGHLDAMQLTGGSSLLNGMYFFRGEVPLAEFVASQPKLVGYGLKFYGPKIFPT      299
YqiG            ----YTLVDALGDK----------ELD---------YLHISLMDV------------NSK      267
Chr-OYE1        ---MEEWLLPLKDA----------GVD---------IFHCSQRRF------------WEP      255
AfOYE1          VAQSIRLALLLADR----------GVD---------LVDVSSGGI------------HAK      315
YqjM            IADHIGFAKWMKEQ----------GVD---------LIDCSSGAL------------VH-      255
PETNR           DALY--LIEELAKR----------GIA---------YLHMSETDL------------A--      277
OYE1            VAQYAYVAGELEKRAK-----AGKRLA---------FVHLVEPRV------------TN-      295
                          :              :           .      .

Nox             YPF----EEGFFLPFARQFRQALRMPLI-------------LLGGINRV----DTI-EHA      337
YqiG            AR---RGADP-TRTRMDLLNERVGNKVPL-----------IAVGSIHSA----DDALAVI      308
Chr-OYE1        ------EFEGSDLNFAGWAKKITGQPTITVGSVGLEGDFMAAFGGQGTEKADLTELTKRL      309
AfOYE1          SAIAIRSGPGYQVHFAQEIKKAVGEK-LL----------ISAVGGIKTG----ALAEEVV      360
YqjM            --ADINVFPGYQVSFAEKIREQA--D-MA----------TGAVGMITDG----SMAEEIL      296
PETNR           ------GGKPYSEAFRQKVRERFHG--VI----------IGA-GAYTAE----KAE-DLI      313
OYE1            -PFLTEGEGEYEGGSNDFVYSIWKG--PV----------IRA-GNFALH----PEVVREE      337
                                    .                      *

Nox             LDEGFEFVAMARALLRDPQLVNKFQAESVDQGLCIHCNKCMPTIYTGTRCVVRDALVVRE      397
YqiG            EN-GIPLVAMGREILVDPDWTVKVKEGREKQIETV--------IKGTD------KEKYHL      353
Chr-OYE1        ERGDFDLVAVGRALLQDPEWAKKVKEQNTEALLDF-------SAESLG------VL----      352
AfOYE1          QSGIDA-VQAGRWFQQNPGLVRAFANELGVKVRMATQ--IDWSFEGRG------KKAKKS      411
YqjM            QNGRADLIFIGRELLRDPFFARTAAKQLNTEIPAPVQ--YERGW----------------      338
PETNR           GKGLIDAVAFGRDYIANPDLVARLQKKAELNPQRP------ESFYGGG------AEGYTD      361
OYE1            VKDKRTLIGYGRFFISNPDLVDRLEKGLPLNKYDR------DTFYQMS------AHGYID      385
                       :  .*    :*  .

Nox             APRLGQ------------      403
YqiG            PEPLWQAIVNTPGWVPYKD     372
Chr-OYE1        Y-----------------      353
AfOYE1          --SL--------------      413
YqjM            ------------------      338
PETNR           YPSL--------------      365
OYE1            YPTYEE--ALKLGWDKK--     400
```

**Figure S1**. Sequence alignment performed by Clustal Omega of the *Af*OYE1 from *Aspergillus flavus* with representative OYEs from different classes: PETNR (Class I), OYE1 (Class II), YqjM (Class III), *Chr*-OYE1 (Class IV), Nox (Class V), and YqiG (Class VI). The catalytic residues are highlighted in blue, and the fingerprint motifs are highlighted in purple.

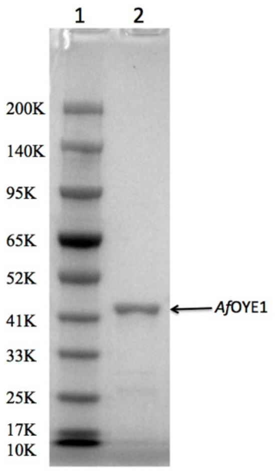


**Figure S2.** SDS-PAGE analysis of the purified AfOYE1. Lane 1: Protein ladder; Lane 2: The purified AfOYE1. The molecular weight of the purified AfOYE1 was at 43.4 KDa ranging between 41 KDa to 52 KDa. 10 uL of purified AfOYE1 was loaded on a 4-12% polyacrylamide gradient gel, and stained with Coomassie Blue.

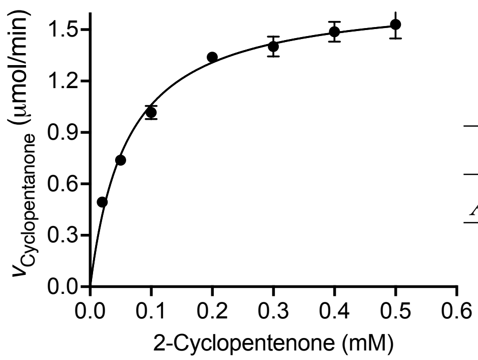


| | $K_m$ ($mM^{-1}$) | $k_{cat}$ ($min^{-1}$) | $k_{cat}/K_m$ ($mM^{-1}s^{-1}$) |
|---|---|---|---|
| *Af*OYE1 | 0.061 ± 0.005 | 34.12 ± 0.74 | 9.32 |

**Figure S3**. Steady-state kinetics of *Af*OYE1-catalyzed reduction of 2-cyclopentenone. Steady-state kinetics were measured in triplicates with varying concentrations of 2-cyclopentenone and fitting the data to the Michaelis-Menten equation. Errors limits are standard deviations.

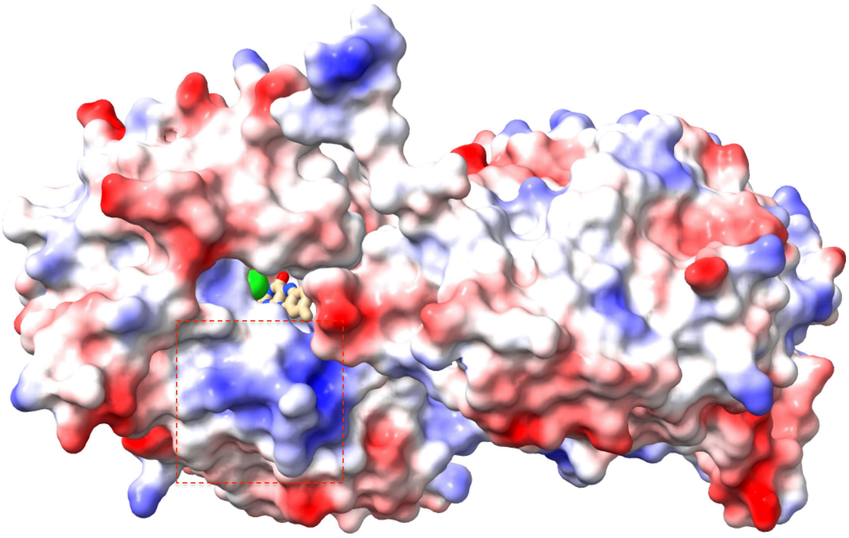

**Figure S4.** Surface charge distribution of *Af*OYE1, calculated by ChimeraX APBS software. Density with red potentially means negative, blue means positive, and white means neutral. Loop from S316 to G325 of *Af*OYE1 labeled as the red dashed rectangle is brimming with the positive surface.

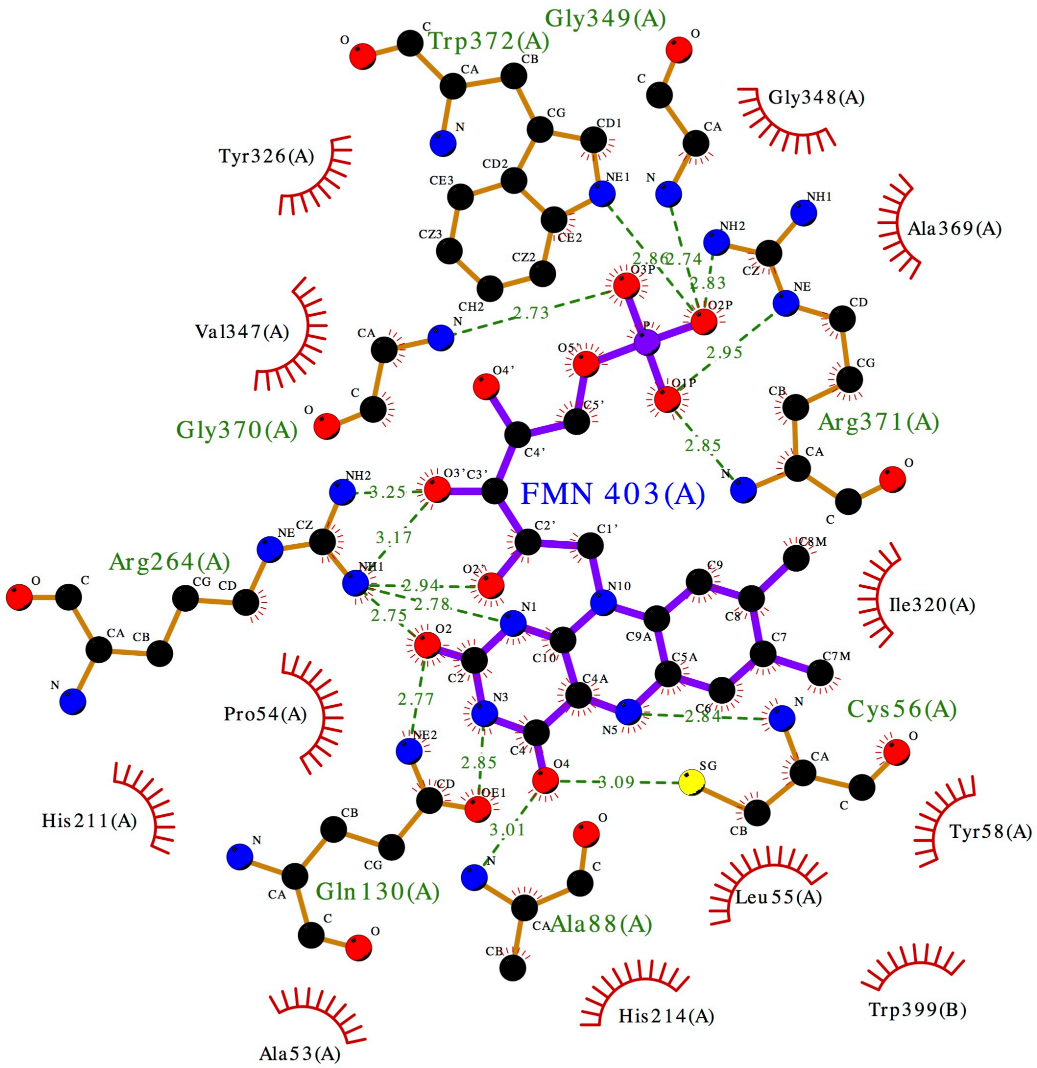


**Figure S5.** The plot of FMN cofactor interactions in the actives site of *Af*OYE1. The interactions have been calculated and visualized by LigPlus software. The thin green dotted lines illustrate hydrogen bonds.

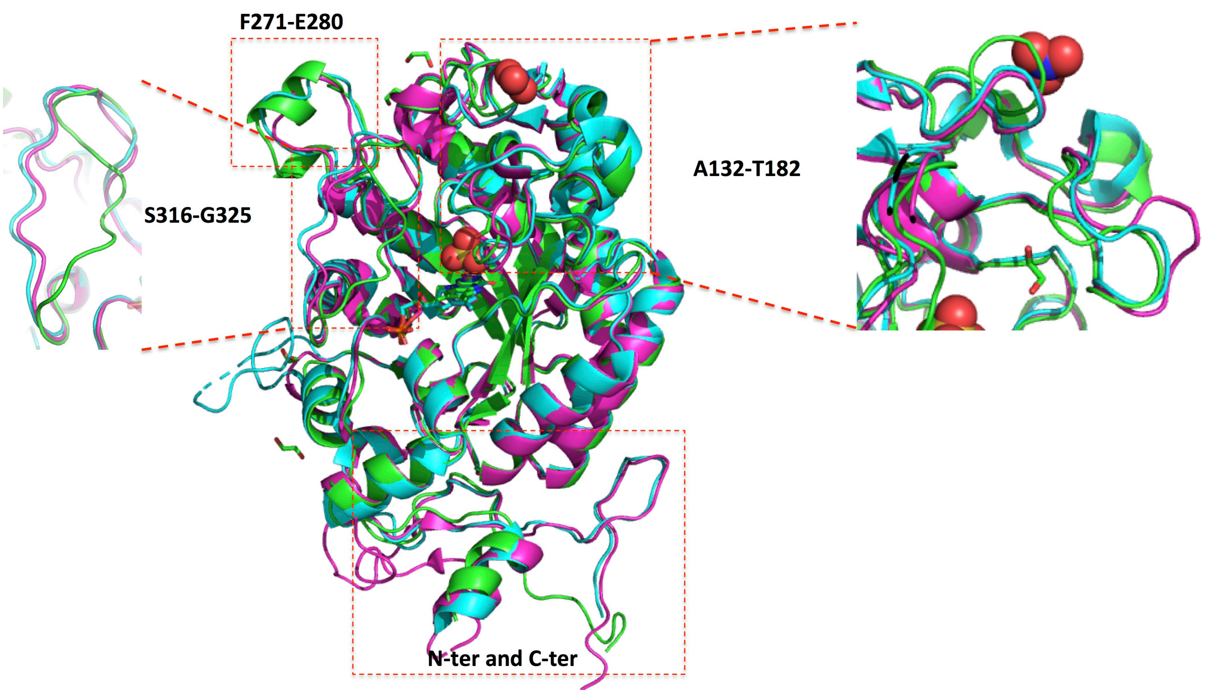


**Figure S6.** Superposition of monomers from the crystal structures of *Af*OYE1 (green), *Bf*OYE4 (cyan), and *An*OYE8 (magenta). Red dashed rectangle labeled as F271-E280 means α6-helix for the residues from Phe271 to Glu280 of *Af*OYE1, S316-G325 means the loop region from Ser316 to Gly325 of *Af*OYE1, A132-T182 means the cap subdomain region from Ala132 to Thr182, and N-ter and C-ter mean N terminal and C-terminal domains of *Af*OYE1.

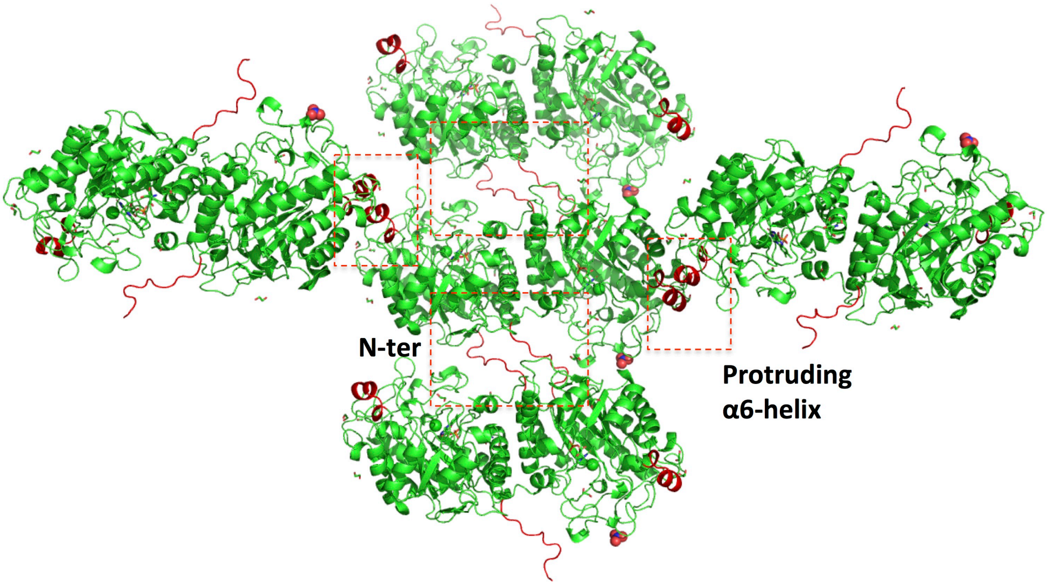


**Figure S7.** A view showing N-terminal loops (red) and protruding α6-helix (red color, residues F271 to E280) of *Af*OYE1 promote dimerization in five copes of crystallographically independent units.

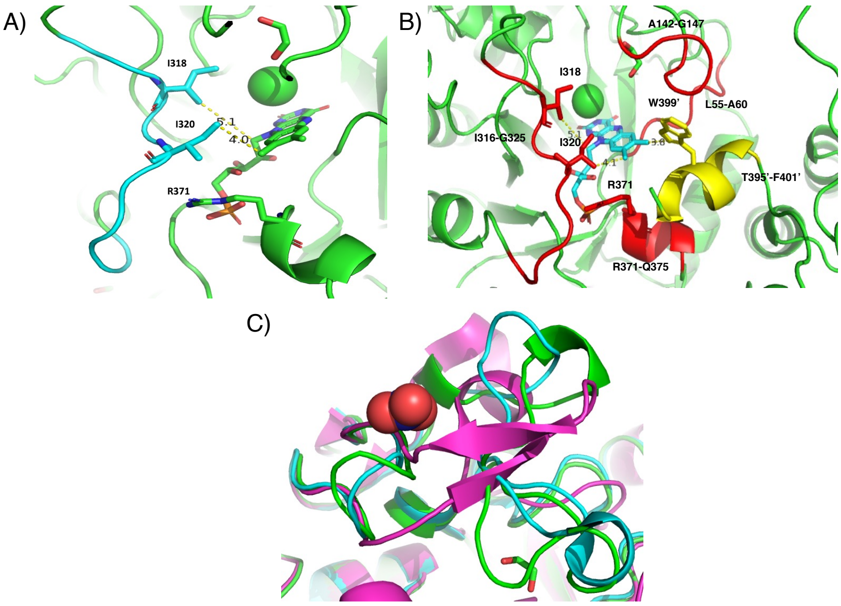


**Figure S8.** Details of the loop region from Ser 316 to Gly 325, the entrance of the catalytic pocket, and the superposition of cap regions from the crystal structures of *Af*OYE1. (A) The loop region from Ser 316 to Gly 325 (cyan) reaches the active site entrance of AfOYE1 (green) and is close to FMN (green). (B) C-terminal α helix (red), residues T395 to F401 (labeled as T395'-F401'), from adjacent protomer pointing to FMN together with three loops and one α-helices (red) (labeled as L55-A60, A142-G147, I316-G325, and R371-Q375) contribute to the narrow entrance of catalytic pocket. (C) Superposition of cap regions from the crystal structures of *Af*OYE1 (green), *Ca*OYE (cyan), and *GS*OYE (magenta).

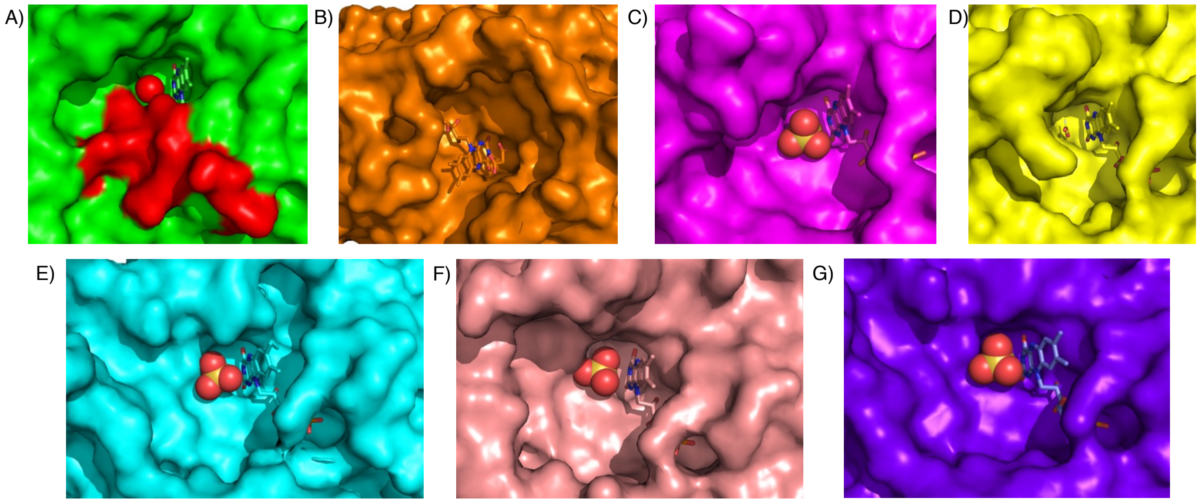


**Figure S9.** Surface representation of catalytic pocket among members of thermophilic-like Class III OYEs: (A) *Af*OYE1, (B) *Ca*OYE, (C) *An*OYE8, (D) *Bf*OYE4, YqjM (cyans), (F) *Ts*OYE, and (G) *Gk*OYE. Residues 316 to 325 in *Af*OYE1 close to the entrance of the catalytic pocket show red.

**Table S1**. Data collection and refinement statistics for *Af* OYE1.

| | |
|---|---|
| **Data collection** | |
| Wavelength (Å) | 0.9793 |
| Resolution range (Å) | 42.86 - 1.55 (1.605 - 1.55) |
| Space group | P 1 21 1 |
| Unite cell | |
| a, b, c (Å) | 47.47, 171.42, 50.48 |
| α, β, γ (°) | 90, 117.60, 90 |
| Total reflections | 667572 (53857) |
| Unique reflections | 102393 (9786) |
| Multiplicity | 6.5 (5.5) |
| Completeness (%) | 99.28 (95.09) |
| $I/\sigma$ ($I$) | 25.71 (7.66) |
| R-merge (%) | 0.05 (0.17) |
| R-meas (%) | 0.05 (0.19) |
| R-pim (%) | 0.02 (0.080) |
| CC1/2 | 0.99 (0.98) |
| **Refinment** | |
| Reflections used in refinement | 102356 (9786) |
| Reflections/#R-free | 4940 (467) |
| R-work/R-free (%) | 0.1443 (0.1622) / 0.1676 (0.1954) |
| # of non-hydrogen | 6997 |
| Protein | 5965 |
| Ligands | 112 |
| Solvent | 920 |
| Protein residues | 774 |
| r. m. s. deviation | |
| Bond lengths (Å) | 0.007 |
| Bond angle (°) | 0.95 |
| Ramachandran plot | |
| Favored | 96.88 |
| Allowed | 3.12 |
| Outliers | 0.00 |
| Average B factor | 11.12 |
| Protein | 9.78 |
| Ligands | 12.37 |
| Solvent | 19.66 |

**Table S2.** List of OYEs used for phylogenetic analysis and their accession numbers.

| Enzyme | Class | Accession ID | Source |
|---|---|---|---|
| AcaryoER1 | I | ABW29811 | *Acaryochloris marina* MBIC11017 |
| AcaryoER3 | I | ABW32756 | *Acaryochloris marina* MBIC11017 |
| Achr-OYE4 | I | AFK73188.1 | *Achromobacter* sp. JA81 |
| *Chr*-OYE2 | I | ALE60337 | *Chryseobacterium* sp. CA49 |
| CtOYE | I | RUT11138.1 | *Chroococcidiopsis thermalis* |
| CyanothER1 | I | ACK64210 | *Rippkaea orientalis* PCC 8801 |
| CyanothER2 | I | ACK65723 | *Rippkaea orientalis* PCC 8801 |
| GluER | I | AAW60280 | *Gluconobacter oxydans* 621H |
| *Gs*OYE | I | XP_005703492 | *Galdieria sulphuraria* |
| MR | I | AAC43569.1 | *Pseudomonas putida* |
| NCR | I | AAV90509 | *Zymomonas mobilis* |
| NemA | I | BAA13186 | *Escherichia coli* |
| NerA | I | CAA74280 | *Agrobacterium tumefaciens* |
| NospuncER1 | I | ACC84535 | *Nostoc punctiforme* PCC 73102 |
| NostocER1 | I | BAB73564 | *Nostoc* sp. PCC 7120 |
| LyngbyaER1 | I | EAW37813 | *Lyngbya* sp. PCC 8106 |
| PETNR | I | AAB38683.1 | *Enterobacter cloacae* |
| SYE1 | I | AAN55488 | *Shewanella oneidensis* MR-1 |
| SYE3 | I | AAN57126 | *Shewanella oneidensis* MR-1 |
| SynER | I | ABB56505 | *Synechococcus elongatus* PCC 7942 |
| XenB | I | AAF02539.1 | *Pseudomonas fluorescens* |
| XenB2 | I | AGS77941.1 | *Pseudomonas putida* |
| YersER | I | WP_032896199 | *Yersinia bercovieri* |
| *Ar*OYE1 | II | AHL17019 | *Ascochyta rabiei* |
| *Ar*OYE2 | II | AHL17020 | *Ascochyta rabiei* |
| *At*OPR1 | II | NP_177794.1 | *Arabidopsis thaliana* |
| AtOPR3 | II | NP_001077884.1 | *Arabidopsis thaliana* |
| ClER | II | EEQ40235 | *Clavispora lusitaniae* ATCC 42720 |
| *Cr*OYE2 | II | XP_001699408.1 | *Chlamydomonas reinhardtii* |
| CYE | II | BAD24850 | *Kluyveromyces marxianus* |
| HYE1 | II | KAG7904883.1 | *Ogataea polymorpha* |
| HYE2 | II | AAN09953 | *Ogataea polymorpha* |
| KYE1 | II | AAA98815.1 | *Kluyveromyces lactis* |

| | | | |
|---|---|---|---|
| *Le*OPR1 | II | NP_001234781.1 | *Solanum lycopersicum* |
| *Le*OPR2 | II | NP_001233868 | *Solanum lycopersicum* |
| LeOPR3 | II | NP_001233873.1 | *Solanum lycopersicum* |
| EBP1 | II | AAA18013 | *Candida albicans* |
| *Mg*ER | II | EDK41665 | *Meyerozyma guilliermondii* ATCC 6260 |
| OYE1 | II | Q02899 | *Saccharomyces pastorianus* |
| OYE2 | II | Q03558 | *Saccharomyces cerevisiae* S288C |
| OYE2.6 | II | ABN66026 | *Scheffersomyces stipitis* CBS 6054 |
| OYE3 | II | P41816 | *Saccharomyces cerevisiae* S288C |
| Achr-OYE3 | III | AFK73187.1 | *Achromobacter* sp. JA81 |
| *Af*OYE1 | III | XP_041144250.1 | *Aspergillus flavus* NRRL3357 |
| AnabaenaER3 | III | ABA25236.1 | *Trichormus variabilis* ATCC 29413 |
| *An*OYE8 | III | XP_001399273.1 | *Aspergillus niger* |
| *Bf*OYE | III | XP_001554780.1 | *Botrytis cinerea* B05.10 |
| *Ca*OYE | III | WP_015941499.1 | *Chloroflexus aggregans* |
| *Chr*-OYE3 | III | AFK73187.1 | *Achromobacter* sp. JA81 |
| *Cr*OYE1 | III | XP_042928859.1 | *Chlamydomonas reinhardtii* |
| *Dr*ER | III | AAF11740 | *Deinococcus radiodurans* R1 |
| FOYE-1 | III | KRH78075 | *Ferrovum* sp. JA12 |
| *Geo*ER | III | BAO37313 | *Geobacillus* sp. #30 |
| *Gk*OYE | III | BAD76617 | *Geobacillus kaustophilus* HTA426 |
| GloeoER | III | BAC91769 | *Gloeobacter violaceus* PCC 7421 |
| OYERo2 | III | ALL54975 | *Rhodococcus opacus* 1CP |
| PfvC | III | AFF18622.1 | *Arthrobacter* sp. JBH1 |
| RhrER | III | AMD82542.1 | *Rhodococcus rhodochrous* ATCC 17895 |
| *Rm*ER | III | ABF11721 | *Cupriavidus metallidurans* CH34 |
| TOYE | III | RUT11138.1 | *Thermoanaerobacter pseudethanolicus* ATCC |
| *Ts*OYE | III | CAP16804 | *Thermus scotoductus* SA-01 |
| XenA | III | AAF02538 | *Pseudomonas putida* |
| YqjM | III | BAA12619 | *Bacillus subtilis* |
| *Chr*-OYE1 | IV | ALE60336.1 | *Chryseobacterium* sp. CA49 |
| Ppo-ER1 | IV | AHC19521 | *Paenibacillus polymyxa* CR1 |
| Ppo-ER2 | IV | AIW41616 | *Paenibacillus polymyxa* CR1 |
| Ppo-ER3 | IV | QBR53093 | *Paenibacillus polymyxa* |
| Rer-ER7 | IV | QBR53095.1 | *Rhodococcus erythropolis* |
| *Ang*OYE11 | V | XP_001390672.2 | *Aspergillus niger* CBS 513.88 |

| | | | |
|---|---|---|---|
| *Ang*OYE12 | V | XP_001395504.2 | *Aspergillus niger* CBS 513.88 |
| *Ar*OYE3 | V | AHL17021 | *Ascochyta rabiei* |
| *Ar*OYE6 | V | AHL17024 | *Ascochyta rabiei* |
| *Bf*OYE6 | V | XP_001547575.1 | *Botrytis cinerea* B05.10 |
| FbOYE | V | TAL24079.1 | *Frankiales bacterium* |
| MoOYE4 | V | XP_003720070.1 | *Pyricularia oryzae* 70-15 |
| Nox | V | WP_029256809.1 | *Rhodococcus erythropolis* |
| PcOYE8 | V | XP_002560444.1 | *Penicillium rubens Wisconsin* 54-1255 |
| BcOYE | VI | WP_098362597.1 | *Bacillus cereus* |
| LacER | VI | ADK19581 | *Lacticaseibacillus paracasei* |
| Lla-ER | VI | WP_046781786.1 | *Lactococcus cremoris* |
| *Mf*OYE | VI | WP_039343246.1 | *Burkholderia* |
| POYE | VI | WP_119715197.1 | *Propionibacterium ruminifibrarum* |
| YqiG | VI | QBR53092 | *Bacillus subtilis* |

**Table S3**. Polymerase chain reaction primers for mutagenesis.

| | Oligonucleotides |
|---|---|
| H211L-F | 5’-TTC GAT GCC ATC GAG ATC CTG GCG GCG CAT GGT TAT CTG-3’ |
| H211L-R | 5’-CAG ATA ACC ATG CGC CGC CAG GAT CTC GAT GGC ATC GAA-3’ |
| H214L-F | 5’-ATC GAG ATC CAT GCG GCG CTG GGT TAT CTG CTG CAC CAG-3’ |
| H214L-R | 5’-CTG GTG CAG CAG ATA ACC CAG CGC CGC ATG GAT CTC GAT-3’ |
| Y216F-F | 5’-ATC CAT GCG GCG CAT GGT TTT CTG CTG CAC CAG TTT CTG-3’ |
| Y216F-R | 5’-CAG AAA CTG GTG CAG CAG AAA ACC ATG CGC CGC ATG GAT-3’ |

**Table S4**. Comparison of *Af*OYE1 characteristics with those of other thermophilic-like Class III OYEs. In the crystal, oligomerization data were checked from crystallographic structures; Interface area, numbers of interacting residues, and interactions involving the dimerization surface were calculated by PISA software; Catalytic pocket volume, surface, and depth were calculated by Proteins. Plus/DoGSiteScorer software. Hb: Hydrogen bonds. Sb: Salt bridges.

| Protein (PDB ID) | In crystal | Interface (Å$^2$) | Interacting residues | Interactions (Hb, Sb) | Pocket Volume (Å$^3$) | Pocket Surface (Å$^2$) | Pocket Depth (Å) |
|---|---|---|---|---|---|---|---|
| *An*OYE8 (7QFX) | tetramer | 2796 | 71 | (18, 4) | 703.38 | 864.99 | 16.97 |
| *Ca*OYE (7O0T) | tetramer | 1451 | 34 | (16, 8) | 758.08 | 748.47 | 22.74 |
| *Bf*OYE4 (7BLF) | dimer | 2121 | 53 | (12, 3) | 829.18 | 838.56 | 26.51 |
| YqjM (1Z41) | dimer | 660 | 31 | (4, 3) | 818.95 | 849.48 | 23.74 |
| *Ts*ER (3HF3) | tetramer | 1262 | 33 | (17, 5) | 653.90 | 690.12 | 20.21 |
| *Gk*OYE (3GR7) | dimer | 1199 | 30 | (16, 0) | 877.28 | 818.83 | 19.34 |
| *Af*OYE1 (8J59) | dimer | 492 | 41 | (7, 6) | 1203.07 | 1126.53 | 24.23 |

**14. The data of the products from the *Af*OYE1-catalyzed bioreduction**

Cyclopentanone (**1b**)

Colorless oil; $^1$HNMR ($CDCl_3$): δ = 2.13-2.17 (m, 4H), 1.93-1.97 (m, 4H) ppm.

(*S*)-2-methylcyclopentanone (**2b**)

Colorless oil; $[\alpha]^{25}_D$ = +16.5° (c 0.4, $CHCl_3$), the optical rotation indicates its configuration to be (*S*) (lit. (Shimoda et al. 2004), $[\alpha]^{25}_D$ = +114.9° (c 0.52, $CHCl_3$); retention time: $t_R$(*S*) 12.31 min (Chiral GC with CHIRASIL-DEX CB column); $^1$HNMR ($CDCl_3$): δ = 2.19-2.33 (m, 2H), 2.05-2.15 (m, 2H), 1.95-2.03 (m, 1H), 1.74-1.82 (m, 1H), 1.42-1.53 (m, 1H), 1.08 (d, J = 6.96 Hz, 3H) ppm.

Cyclohexanone (**3b**)

Colorless oil; $^1$HNMR ($CDCl_3$): δ = 2.31-2.34 (m, 4H), 1.82-1.88 (m, 4H), 1.69-1.74 (m, 2H) ppm.

(*S*)-2-methylcyclohexanone (**4b**)

Colorless oil; retention time: $t_R$(*S*) 7.26 min (Chiral GC with CHIRASIL-DEX CB column); $^1$HNMR ($CDCl_3$): δ = 2.29-2.35 (m, 2H), 2.18-2.26 (m, 1H), 1.98-2.04 (m, 2H), 1.76-1.80 (m, 1H), 1.55-1.64 (m, 2H), 1.29-1.35 (m, 1H), 0.96 (d, J = 6.60 Hz, 3H) ppm.

Propionamide (**5b**)

Colorless oil; $^1$HNMR ($CDCl_3$): δ = 2.26 (q, J = 7.86 Hz, 2H), 0.96 (t, J = 7.86 Hz, 3H) ppm.

Phenylnitroethane (**6b**)

Colorless oil; $^1$HNMR ($CDCl_3$): δ = 7.30-7.37 (m, 3H), 7.22-7.24 (m, 2H), 4.64 (t, J = 7.34 Hz, 2H), 3.35 (t, J = 7.34 Hz, 2H) ppm.

1-Phenyl-2-nitropropane (**7b**)

Colorless oil; $^1$HNMR ($CDCl_3$): δ = 7.29-7.36 (m, 3H), 7.18-7.20 (m, 2H), 4.78-4.83 (m, 1H), 3.36 (dd, J = 7.52 Hz, J = 14.00 Hz, 1H), 3.04 (dd, J = 6.80 Hz, J = 14.00 Hz, 1H), 1.57 (d, J = 6.66 Hz, 3H) ppm.

Citronellal (**8b**)

Colorless oil; $^{1}$HNMR ($CDCl_3$): δ = 5.06-5.10 (m, 1H), 2.37-2.43 (m, 1H), 2.19-2.26 (m, 1H), 1.96-2.09 (m, 3H), 1.68 (s, 3H), 1.60 (s, 3H), 1.24-1.39 (m, 2H), 0.97 (d, J = 6.66 Hz, 3H) ppm.

**15. Selected $^1$H NMR spectra and GC chromatograms**

Cyclopentanone (**1b**)

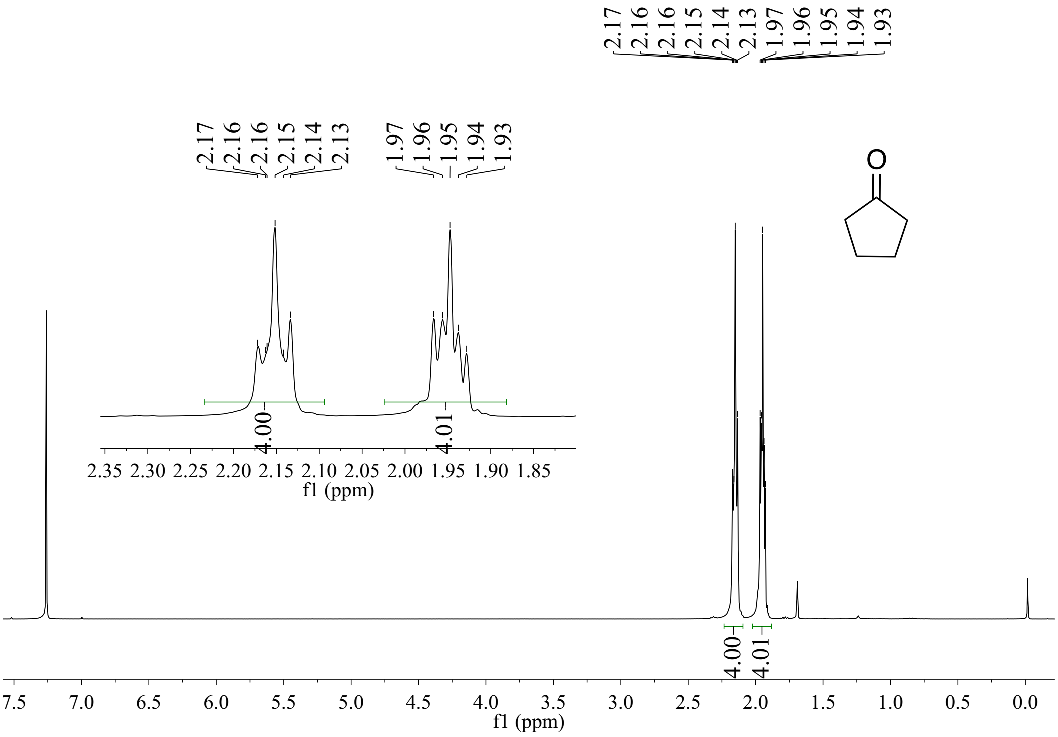

(*S*)-2-methylcyclopentanone (**2b**)

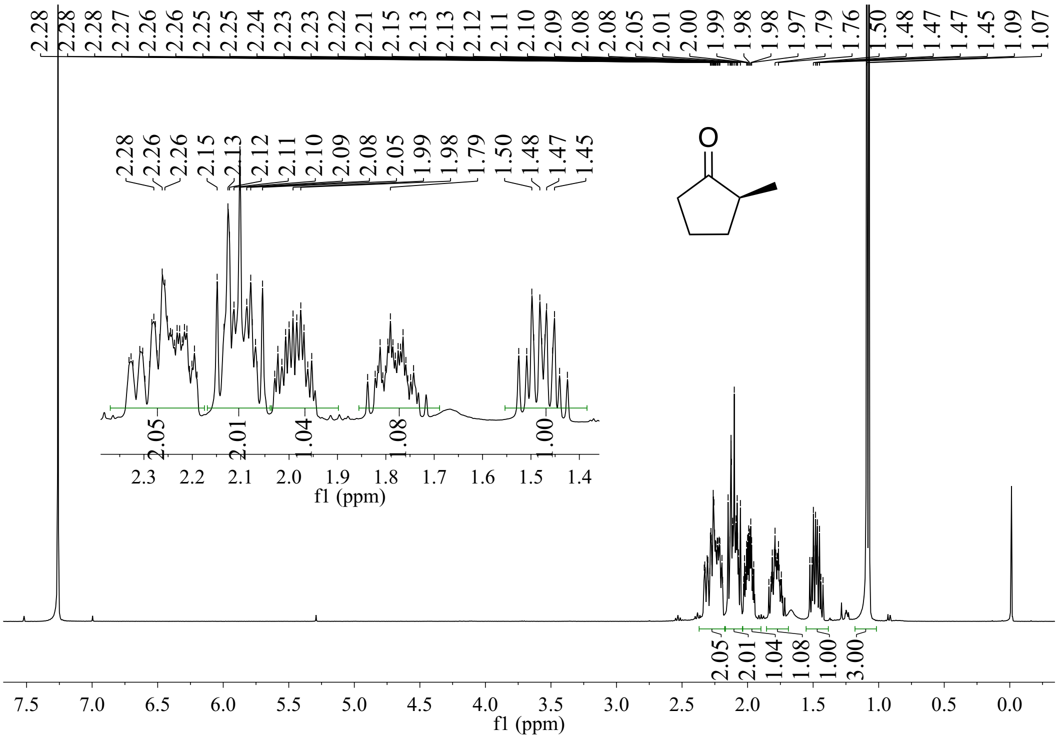

GC chromatogram of Racemic 2-methylcyclopentanone and (*S*)- 2-methylcyclopentanone (**2b**) from *Af*OYE1-catalyzed bioreduction

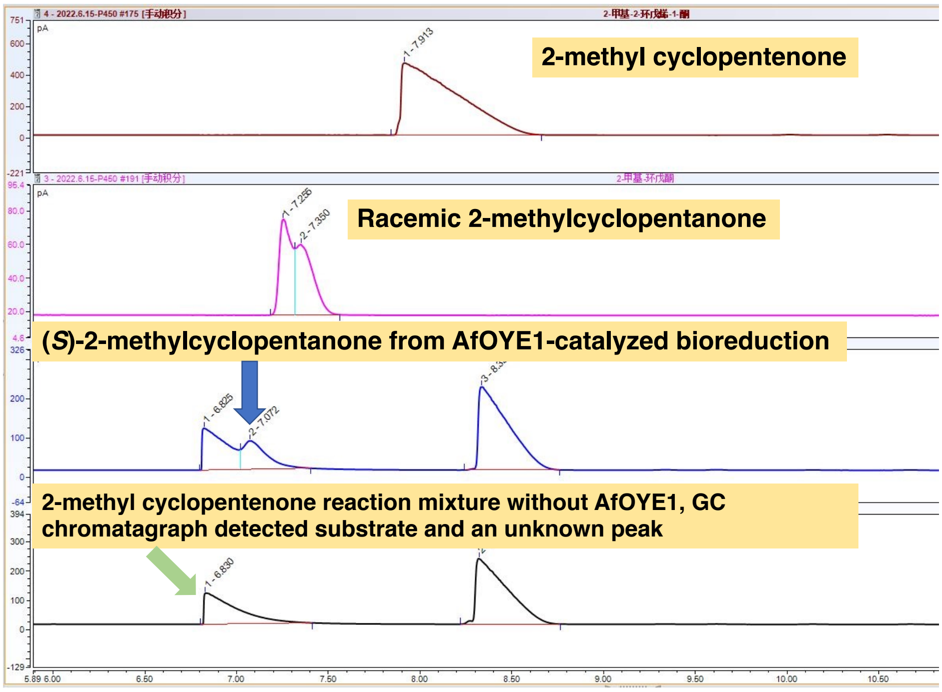


Cyclohexanone (**3b**)

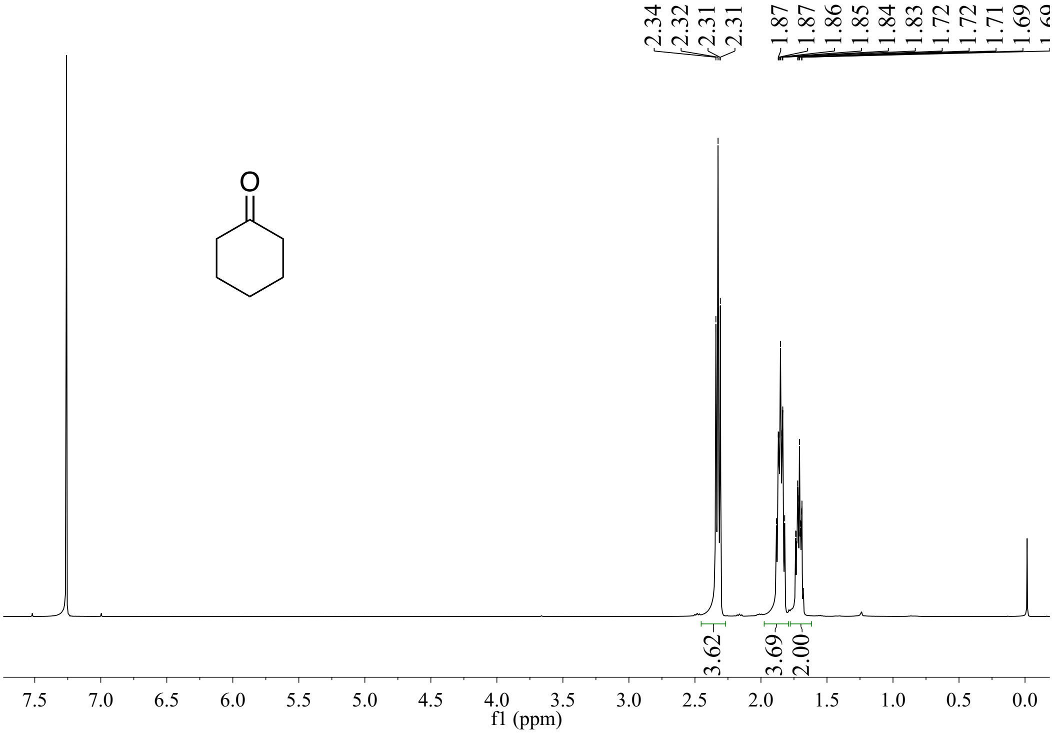

(*S*)-2-methylcyclohexanone (**4b**)

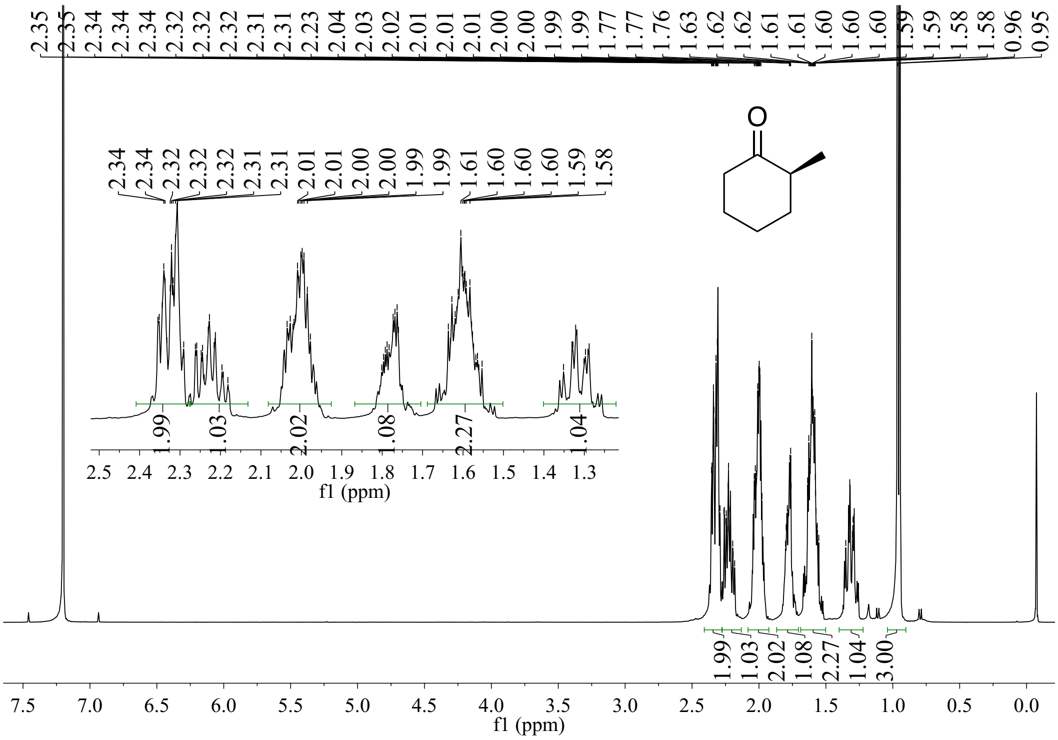


GC chromatogram of Racemic 2-methylcyclohexanone and (*S*)-2-methylcyclohexanone (**4b**) from *Af*OYE1-catalyzed bioreduction

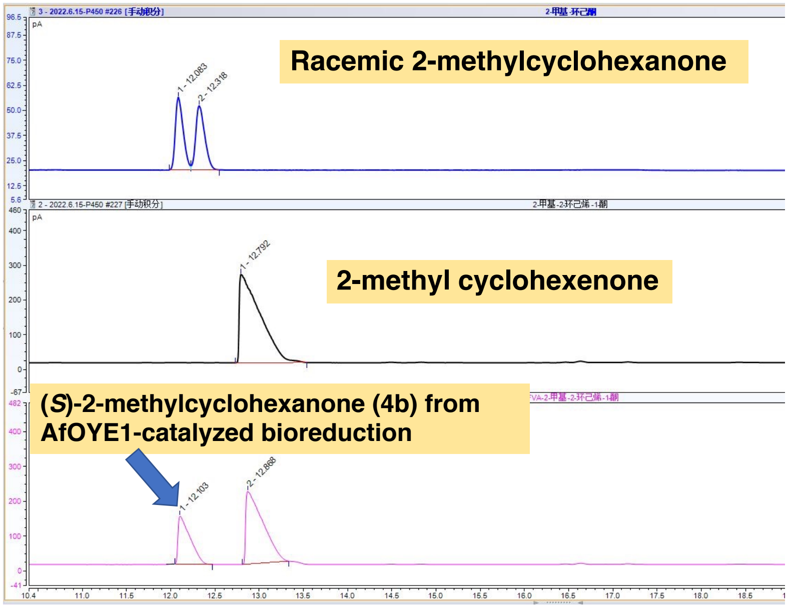

Propionamide (**5b**)

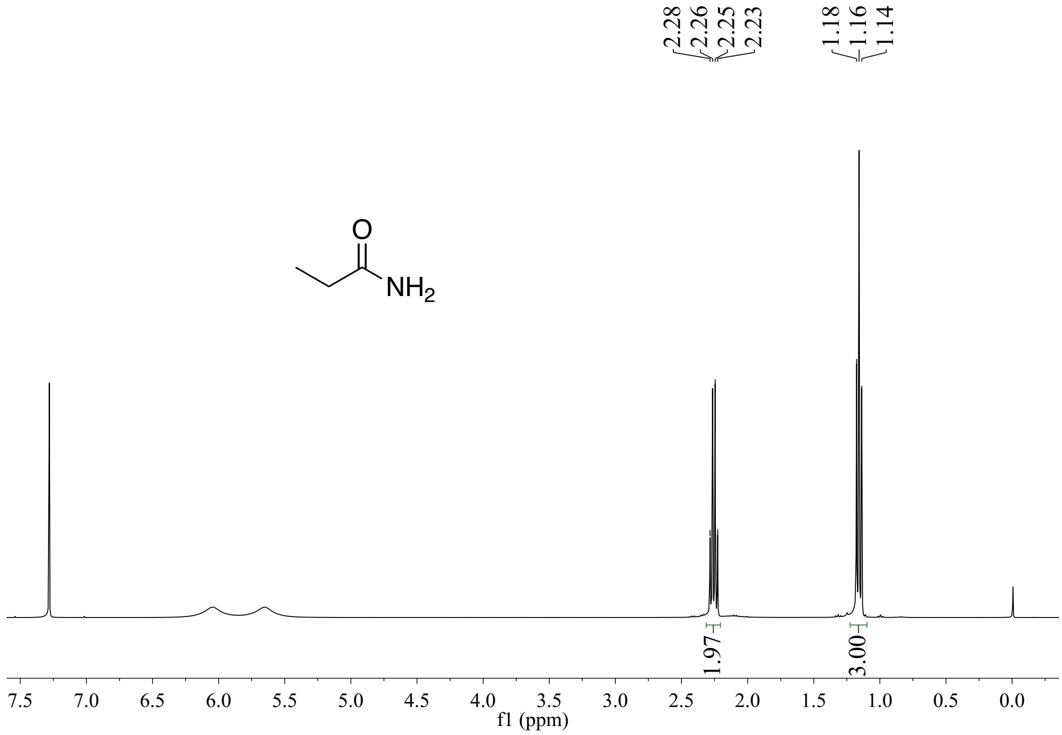


Phenylnitroethane (**6b**)

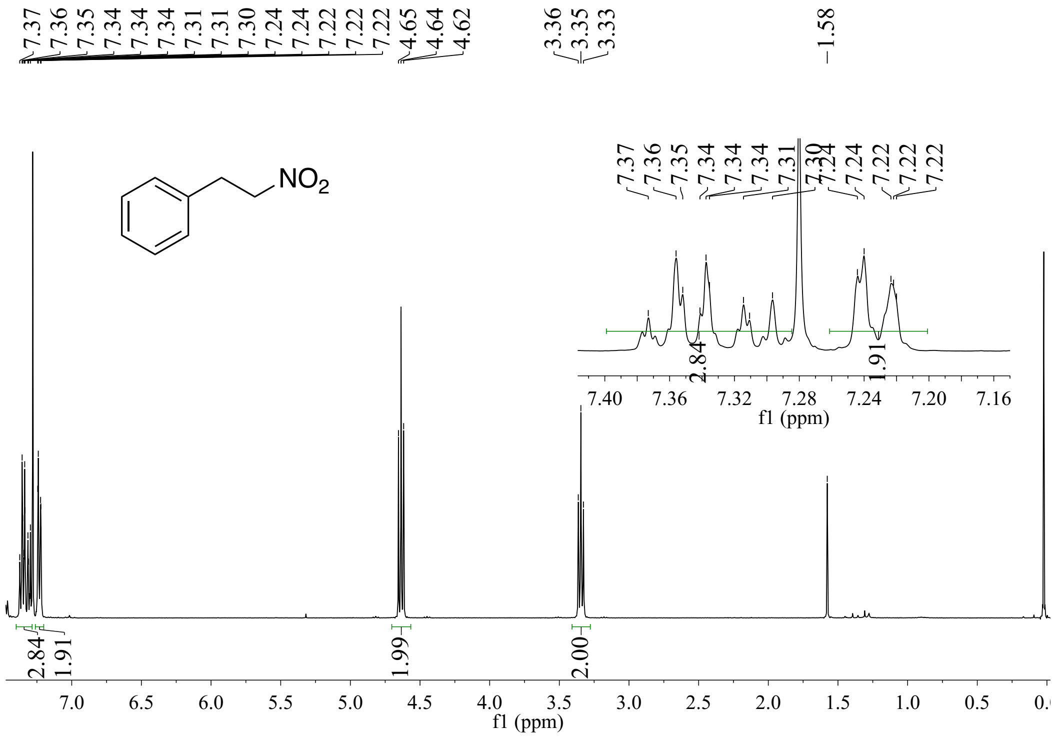

1-Phenyl-2-nitropropane (**7b**)

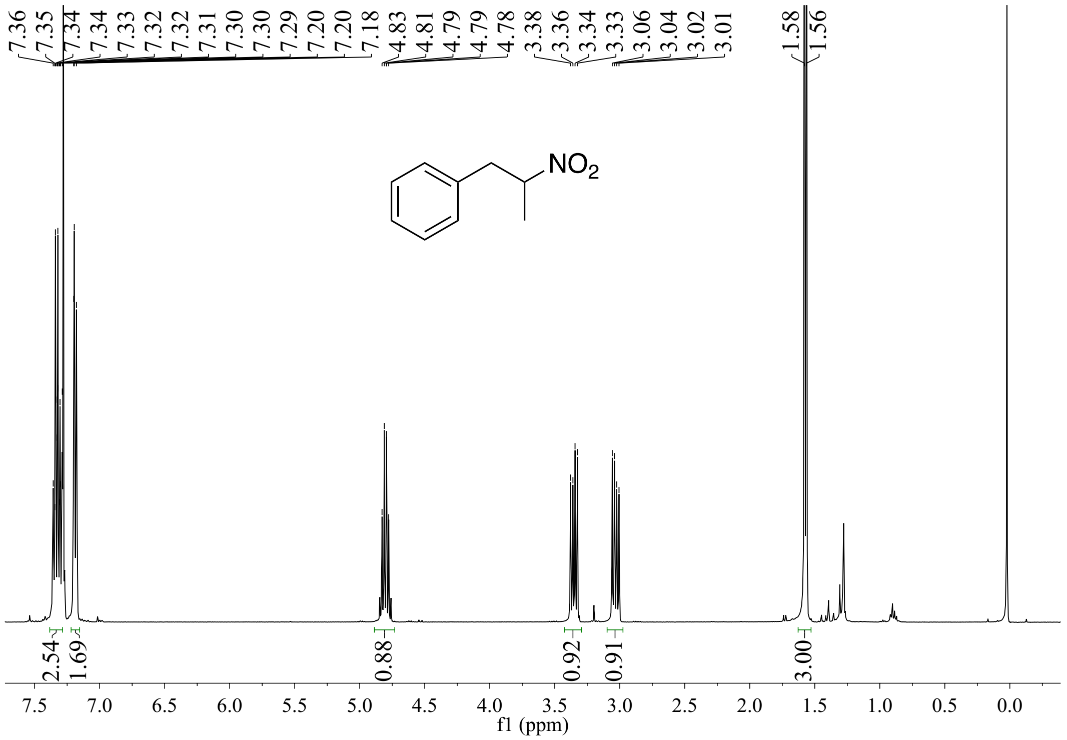


Citronellal (**8b**)

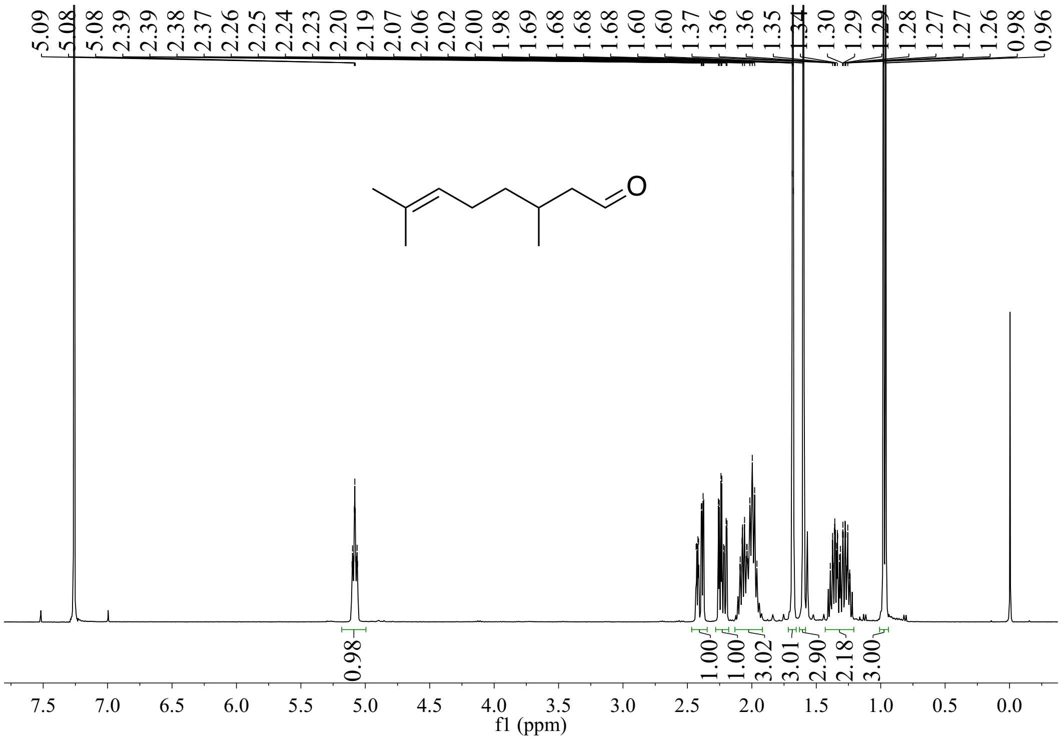

## 16. The sequence information of *Af*OYE1.

The amino acid sequence of afvA (UniProt: B8N8Q9, *Af*OYE1) with N-terminal His6-tag followed by a tobacco etch virus (TEV) protease cleavage site.

MGSSHHHHHHENLYFQGISYYTPAQVPPAGTQVEGSTKLFSPLTIRGVTFPNRLFLAP
LCQYSAKDGYANDWHLTHIGGIVQRGPGLAIMEATAVQKVGRITPQDLGLYDDGHI
EPLKRITEFAHSQSQKIGIQLAHAGRKASAVAPWLSGNAMAVKEVGGWPDDIVAPS
AIPQEEGINAVPKVLTGEDIGVLKKDWAEAAKRAVRANFDAIEIHAAHGYLLHQFLS
PVSNRRTDKYGGSFENRVRILLEICEEVRAVIPTAMPLLVRISATDWFEFDDNLTKEFP
ESWTVAQSIRLALLLADRGVDLVDVSSGGIHAKSAIAIRSGPGYQVHFAQEIKKAVG
EKLLISAVGGIKTGALAEEVVQSGIDAVQAGRWFQQNPGLVRAFANELGVKVRMAT
QIDWSFEGRGKKAKKSSL

The codon-optimized nucleotide sequence of *afvA*.

ATGGGCAGTAGTCATCACCACCATCACCACGAGAATCTGTACTTCCAAGGCATCA
GTTACTATACCCCAGCGCAAGTTCCGCCGGCCGGTACGCAAGTTGAAGGTAGCA
CCAAGCTGTTCAGCCCGCTGACGATTCGCGGCGTGACGTTTCCGAATCGTCTGTT
TCTGGCCCCACTGTGCCAGTACAGTGCGAAAGATGGCTATGCGAACGACTGGCA
TCTGACCCACATTGGTGGCATTGTTCAGCGTGGCCCGGGTCTGGCCATCATGGAA
GCGACCGCCGTTCAAAAAGTTGGCCGTATCACGCCGCAAGATCTCGGTCTGTATG
ATGACGGCCACATCGAGCCACTGAAGCGCATCACCGAGTTCGCCCATAGTCAGA
GCCAGAAGATCGGTATCCAGCTGGCGCACGCGGGTCGTAAAGCCAGTGCCGTTG
CGCCGTGGCTGAGTGGTAATGCGATGGCCGTGAAAGAGGTTGGTGGCTGGCCGG
ATGACATCGTGGCGCCAAGCGCCATCCCGCAAGAAGAGGGCATCAATGCCGTGC
CAAAAGTGCTGACGGGTGAGGACATCGGCGTGCTGAAAAAAGACTGGGCGGAA
GCGGCGAAACGTGCGGTTCGTGCCAACTTCGATGCCATCGAGATCCATGCGGCG
CATGGTTATCTGCTGCACCAGTTTCTGAGTCCGGTTAGCAACCGTCGCACGGACA
AATACGGCGGCAGCTTTGAAAACCGTGTGCGCATTCTGCTGGAGATCTGCGAAG
AAGTTCGCGCGGTGATTCCAACCGCCATGCCGCTCCTCGTTCGCATCAGCGCCAC
GGACTGGTTCGAGTTCGACGACAATCTGACGAAGGAATTCCCGGAGAGCTGGAC
CGTGGCGCAGAGTATCCGTCTGGCGCTGCTGCTGGCGGATCGTGGCGTTGATCTG
GTGGACGTGAGCAGCGGTGGCATTCATGCGAAAAGTGCCATCGCCATCCGTAGT
GGCCCGGGCTACCAAGTTCACTTCGCGCAAGAAATCAAAAAGGCGGTGGGTGAA
AAGCTGCTGATCAGTGCCGTTGGCGGCATTAAAACCGGTGCGCTGGCCGAAGAA
GTTGTTCAGAGTGGCATTGACGCCGTTCAAGCCGGCCGTTGGTTTCAGCAGAACC
CGGGTCTGGTTCGCGCGTTCGCCAACGAGCTGGGTGTGAAGGTTCGTATGGCCAC
GCAGATCGACTGGAGCTTCGAATAA

Reference:

Shimoda K, Kubota N, Hamada H, Kaji M, Hirata T (2004) Asymmetric reduction of enones with *Synechococcus* sp. PCC 7942. Tetrahedron: Asymmetry 15:1677-1679
